# Gastrointestinal Angiodysplasia Resolution After Transcatheter Aortic Valve Implantation

**DOI:** 10.1001/jamanetworkopen.2024.42324

**Published:** 2024-10-30

**Authors:** Lia C. M. J. Goltstein, Maxim J. P. Rooijakkers, Naomi D. E. Thierens, Selene C. M. Schoormans, Antonius E. van Herwaarden, Hanneke Beaumont, Charles Houdeville, Marlijn P. A. Hoeks, Erwin-Jan M. van Geenen, Sanna R. Rijpma, Xavier Dray, Niels van Royen, Joost P. H. Drenth

**Affiliations:** 1 Department of Gastroenterology and Hepatology, Radboud University Medical Center, Nijmegen, the Netherlands; 2 Department of Cardiology, Radboud University Medical Center, Nijmegen, the Netherlands; 3 Department of Laboratory Medicine, Laboratory of Hematology, Radboud University Medical Center, Nijmegen, the Netherlands; 4 Department of Laboratory Medicine, Radboud University Medical Center, Nijmegen, the Netherlands; 5 Department of Gastroenterology and Hepatology, Amsterdam University Medical Center, Amsterdam, the Netherlands; 6 Sorbonne University, Center for Digestive Endoscopy, Hôpital Saint-Antoine, AP-HP, Paris, France; 7 Équipes Traitement de l’information et Systèmes, ETIS UMR 8051, CY Paris Cergy University, France; 8 Department of Hematology, Radboud University Medical Center, Nijmegen, the Netherlands

## Abstract

**Importance:**

Heyde syndrome is the cooccurrence of aortic stenosis and gastrointestinal bleeding secondary to vascular lesions, including angiodysplasias. Several studies have demonstrated cessation of gastrointestinal bleeding after transcatheter aortic valve implantation (TAVI), but the etiology and effects on vascular lesions are largely unknown.

**Objective:**

To examine the associations of TAVI with gastrointestinal vascular lesions and identify factors associated with recovery among patients with iron deficiency anemia and severe aortic stenosis.

**Design, Setting, and Participants:**

In this prospective, single-center cohort study, patients with iron deficiency anemia on the TAVI waiting list from September 2020 to February 2022 were assessed by capsule endoscopy. Those with vascular lesions were reassessed 6 months after TAVI. Endoscopic images were anonymized and evaluated by 2 independent researchers. Data were analyzed from September 2022 to August 2024.

**Exposure:**

TAVI.

**Main Outcomes and Measures:**

The primary outcome was the mean difference in the number of vascular lesions before vs after TAVI.

**Results:**

A total of 24 patients (mean [SD] age, 77.4 [7.1] years; 18 [75.0%] male) underwent capsule endoscopy, and vascular lesions were present in 18 patients (75.0%). TAVI was performed in 15 of 18 patients with vascular lesions, of whom 11 agreed to a second capsule endoscopy. The mean (SD) number of vascular lesions across the gastrointestinal tract decreased from 6.4 (5.6) lesions before TAVI to 2.0 (2.1) lesions 6 months after TAVI (*P* = .04). The number of vascular lesions decreased in 9 of 11 patients (81.8%), including 6 patients (54.5%) who no longer had typical angiodysplasias. Resolution of angiodysplasias was less frequent in patients who had multiple valvular heart disease before TAVI (0 of 3 patients) vs those without multiple valvular heart disease (6 of 8 patients [75.0%]) and in patients with significant paravalvular leakage after TAVI (2 of 5 patients [40.0%]) vs those without significant leakage (4 of 6 patients [66.7%]).

**Conclusions and Relevance:**

In this cohort study of 24 patients with iron deficiency anemia and severe aortic stenosis, angiodysplasias were present in 75.0% of patients. TAVI was associated with reduced size and number of angiodysplasias in these patients. These findings suggest that TAVI not only improves aortic stenosis but may also reduce gastrointestinal bleeding by resolving vascular lesions, offering a dual benefit for patients with Heyde syndrome.

## Key Points

**Question:**

What are the associations of transcatheter aortic valve implantation (TAVI) with gastrointestinal vascular lesions, including angiodysplasias, in patients with Heyde syndrome?

**Findings:**

In this prospective cohort study including 24 patients with severe aortic stenosis and iron-deficiency anemia, 18 patients (75.0%) were diagnosed with gastrointestinal vascular lesions, known as Heyde syndrome. The mean number of vascular lesions significantly decreased from 6.4 to 2.0 lesions per patient 6 months after TAVI.

**Meaning:**

These findings suggest that angiodysplasia-related bleeding was the likely cause of iron deficiency anemia in patients with aortic stenosis, and TAVI was an effective treatment.

## Teaser

This cohort study examines the association of transcatheter aortic valve implantation (TAVI) with gastrointestinal vascular lesions among patients with aortic stenosis and gastrointestinal bleeding secondary to vascular lesions.

## Introduction

Severe aortic stenosis (AS) is accompanied by anemia in approximately 60% of patients.^1,2^ Management of anemia improves clinical outcomes in patients with AS, as these individuals face higher risks of bleeding complications and cardiovascular morbidity.^1,3^ Gastrointestinal bleeding may contribute to the high prevalence of anemia in patients with AS, a correlation recognized since 1958.^4^ Initially considered idiopathic, bleeding was later linked to angiodysplasias with the emergence of small bowel assessment at the beginning of the 21st century.^5,6^ Angiodysplasias are vascular malformations consisting of thin-walled, dilated capillaries throughout the gastrointestinal tract.^6^ The co-occurrence of AS and angiodysplasia-related bleeding became known as Heyde syndrome.^7^ It has been suggested that excessive proteolysis of von Willebrand factor (VWF) due to high shear stress around the stenotic valve is responsible for the bleeding diathesis and angiogenesis-dependent formation of angiodysplasias in Heyde syndrome.^8,9^

Aortic valve replacement is the definitive treatment for AS.^10^ Transcatheter aortic valve implantation (TAVI) has gained traction over surgical valve replacement due to its noninvasive approach.^10^ Multiple studies have found that TAVI not only treats AS but also halts gastrointestinal bleeding in patients with Heyde syndrome.^11,12,13,14^ However, the etiology of angiodysplasias after TAVI remains largely unknown due to a lack of prospective studies on their dynamics.^14^ Such studies could inform clinical decision-making in selecting patients for this procedure. Since angiodysplasias are generally located in the small bowel, they are out of reach for conventional endoscopic studies.^15^ Capsule endoscopy is needed to obtain a comprehensive overview of the gastrointestinal tract.^16^ With this technique, patients swallow a small camera that captures more than 50 000 images as it passes through the intestines.^17^ A 2023 study by Yashige et al^18^ used capsule endoscopy to examine vascular lesion dynamics after TAVI, reporting a significant reduction.^18^ However, the Heyde syndrome prevalence in the study by Yashige et al^18^ far exceeded previous reports, and a causal relationship with VWF was not confirmed. To this end, we designed a prospective cohort study to further evaluate the association of TAVI with the presence and size of vascular lesions in the gastrointestinal tract.

## Methods

The cohort study was approved as an amendment to the APPOSE trial in August 2020 by the medical research ethics committee and the institutional review board of the Radboud University Medical Center. The APPOSE trial is registered in the ClinicalTrials.gov database (identifier: NCT04281771). Written informed consent was obtained from each patient. This study is reported following the Strengthening the Reporting of Observational Studies in Epidemiology (STROBE) reporting guideline.

### Study Population and Design

The study was designed as a prospective single-center study (eFigure 1 in Supplement 1). The study population was consecutive patients with severe AS who were wait-listed for TAVI in the Radboud University Medical Center, had iron deficiency anemia, and consented to capsule endoscopy. Iron deficiency anemia was defined as a hemoglobin less than 13.7 g/dL (to convert to grams per liter, multiply by 10) for men or less than 12.2 g/dL for women and a ferritin less than 100 ng/mL (to convert to micrograms per liter, multiply by 1). This higher ferritin cutoff, compared with the usual 30 ng/mL, is recommended for older adults with heart failure.^19,20^

All patients on the TAVI wait-list were evaluated by a multidisciplinary heart team and deemed eligible. Patients underwent preselection examinations, including laboratory analyses to screen for anemia. Patients with moderate to severe iron deficiency anemia (hemoglobin level <11 g/dL) who had not undergone bidirectional endoscopy in the past 5 years were referred to a gastroenterologist for upper and lower endoscopy. Patients who had an alternative explanation for anemia (eg, gastric ulcer) at a recent endoscopy (<5 years) were excluded from the study. Additionally, patients previously diagnosed with angiodysplasias were included only if they had not received endoscopic treatment or antiangiogenic agents (eg, octreotide) within 6 months before or after undergoing TAVI.^21,22^ Patients who could not undergo capsule endoscopy due to contraindications (eg, luminal gastrointestinal tract obstruction) were also excluded from the study.^23^

### Capsule Endoscopy

Patients underwent baseline capsule endoscopy within 3 months of TAVI using Pillcam Small Bowel 3 capsules (Medtronic). Images were assessed per standard procedures in our center.^17^ Patients with vascular lesions at baseline endoscopy were asked to undergo a follow-up capsule endoscopy approximately 6 months after TAVI. All images were assessed by 2 independent, experienced readers (H.B. and X.D.) for the presence of vascular lesions. The readers were masked to the source and timing of the images (baseline or follow-up). An advanced artificial intelligence solution (AXARO beta version, Augmented Endoscopy) with a high sensitivity and specificity for diagnosing angiodysplasias (97% and 99%, respectively) assisted readers by preselecting images.^24^ Vascular lesions identified by the readers were divided into lesions with an intermediate to high risk of bleeding (P1 and P2 lesions), and lesions with no or a low bleeding potential (P0 lesions) according to the Saurin classification.^25^ Relevant vascular lesions (P1 and P2) were further classified into diminutive angiectasias (P1) and typical angiodysplasias (P2), following 2 consensus statements on the semantic description and clinical relevance of vascular lesions.^26,27^ Active bleeding was also considered to be important in this context (P2) (eTable 1 in Supplement 1). A consensus meeting was held to review all relevant vascular lesions that were only reported once or classified differently by both readers. A third reader reviewed (N.D.E.T.) if consensus was not reached.

### Laboratory Analyses

Blood was drawn from an antecubital vein at 4 time points: during the first capsule endoscopy appointment (T1), within 72 hours after TAVI (T2), at the 3-month follow-up appointment (T3), and during the second capsule endoscopy appointment, approximately 6 months after TAVI (T4). One tube was used for routine blood chemistry, and 2 citrated tubes were stored for additional assays. Blood samples were processed within 60 minutes. Centrifugation was performed at 2800 to 3500 rotations per minute for 10 minutes at 20 °C. Plasma was transferred to a clean container and stored at −80 °C. Additional assays on stored samples from patients with complete follow-up included angiogenesis growth factors (vascular endothelial growth factor [VEGF] and angiopoietin-2) and VWF analyses (VWF antigen [VWF:Ag], VWF ristocetin cofactor [VWF:RCoF], and multimeric pattern). The level of VWF:Ag reflects the quantity of VWF, while the level of VWF:RCoF evaluates VWF activity. The multimeric pattern was determined through protein gel electrophoresis, since larger VWF multimers are more hemostatically active.^28^ A semiautomated VWF multimer assay was used to perform densitometry analyses in a standardized manner.^29,30^ Low–molecular weight multimers were bands 1 to 3; median–molecular weight multimers, bands 4 to 7; and high–molecular weight multimers (HMWM), bands greater than 7.^31^ The relative proportion of VWF-HMWM was determined by dividing the area under the curve of HMWM by the total AUC.^31^ VWF-HMWM proportions were normalized to the mean VWF-HMWM proportion of healthy individuals. The coefficient of variation for the VWF-HMWM proportion was determined to be 9.5%, based on 97 repeated measurements of pooled plasma from healthy individuals. The mean VWF-HMWM proportion for these pooled samples was 51.3%.Assays and software used for analyses are detailed in eTable 2 in Supplement 1.

### Primary and Secondary Outcomes

Our primary outcome measure was the mean difference in the number of relevant (P1 and P2) vascular lesions before and 6 months after TAVI. Vascular lesions were diagnosed through capsule endoscopy.

Secondary outcome measures included the proportion of patients with fewer vascular lesions and those without typical angiodysplasias after TAVI. We also assessed the number of patients who required transfusion units (including red blood cell transfusions per packed cells and intravenous iron infusions per 500 mg) in the 6 months before and after TAVI. Additionally, we evaluated the mean difference in hemoglobin levels, angiogenesis growth factors, and VWF analyses, as well as the number of patients with acquired von Willebrand disease (AVWD), defined by a loss of large multimers resulting in a normalized VWF-HMWM ratio less than 80% compared with healthy individuals or a VWF:RCoF to VWF:Ag ratio less than 0.7, before and after TAVI.^18,32,33^ Finally, we explored the association between factors previously associated with Heyde syndrome and recovery of angiodysplasias after TAVI. These factors include multiple valvular heart disease before TAVI (defined as the combination of moderate to severe regurgitant and/or stenotic lesions on ≥2 heart valves), paravalvular leakage after TAVI (defined as mild to severe), and AVWD after TAVI.^11,34,35^

### Statistical Analysis

The sample size determination was informed by a previous meta-analysis about Heyde syndrome, reporting a pooled gastrointestinal bleeding cessation rate of 72.8% (95% CI, 62.2%-81.3%) after aortic valve replacement. With an assumed effect size of Cohen *d* = 0.8 and a drop-out rate of 10%, approximately 18 patients with Heyde syndrome would be required to detect a difference in the mean number of relevant vascular lesions with 80% power (2-sided test, α = .05).^36^

In the primary and secondary analyses, differences before and after TAVI were determined using the paired samples *t* test for normally distributed data (mean with SD) or the Wilcoxon signed-rank test for nonnormally distributed data (median with IQR). The Shapiro-Wilk test for small sample sizes was used to assess the distribution of differences between paired observations.^37^ The interobserver agreement in detecting relevant vascular lesions was assessed with the Cohen κ test. A 2-tailed *P* ≤ .05 was considered significant in all statistical analyses. Statistical analyses were performed with the SPSS statistical software package version 29.0 (IBM). Data were analyzed from September 2022 to August 2024.

## Results

### Study Population

**Figure 1.**
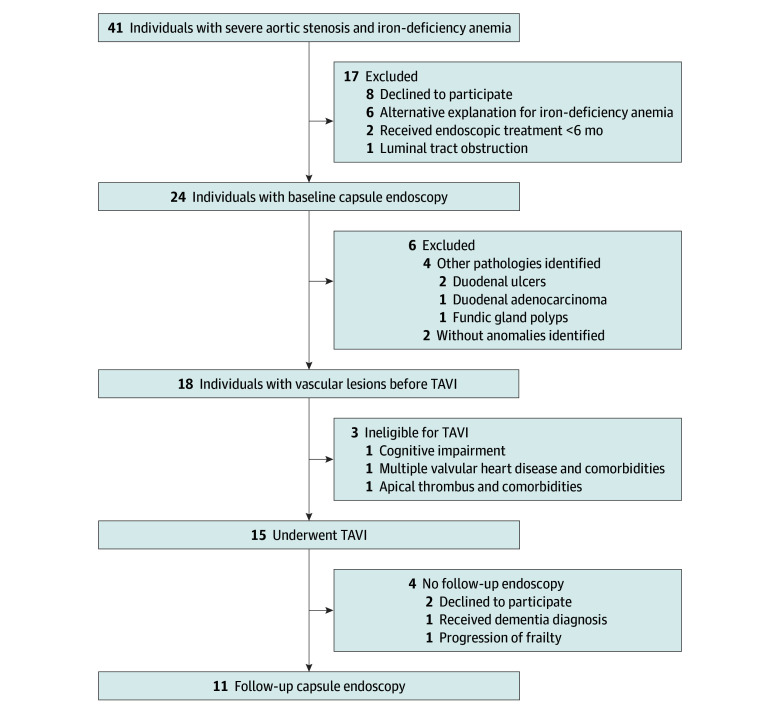
Participant Flowchart TAVI indicates transcatheter aortic valve implantation.

From September 2020 to February 2022, 41 patients met the inclusion criteria. Eight declined participation and 9 were excluded (Figure 1). A total of 24 patients (mean [SD] age, 77.4 [7.1] years; 18 [75.0%] male) agreed to participate. In 5 patients, capsule endoscopy was performed simultaneously with bidirectional endoscopy, and 4 patients had previously been diagnosed with angiodysplasias. At baseline capsule endoscopy, 18 patients (75.0%) had relevant vascular lesions, indicating Heyde syndrome. Most of these patients had multiple lesions (12 patients [66.7%]), including typical angiodysplasias (12 patients [66.7%]), diminutive angiectasias (10 patients [55.6%]), and active bleedings (5 patients [27.8%]). Lesions were primarily located in the small bowel (14 patients [77.8%]), followed by the colon (9 patients [50.0%]) and stomach (4 patients [22.2%]). Table 1 displays the clinical characteristics, echocardiographic parameters, and laboratory results. Compared with patients without Heyde syndrome, patients with Heyde syndrome were more likely to have chronic kidney failure (7 patients [38.9%] vs 0 patients) and coagulopathies (4 patients [22.2%] vs 0 patients). Coagulopathies included thrombocytopenia (2 patients) and thrombocytopathy secondary to myelodysplastic syndrome (2 patients). Aortic valve echocardiographic parameters were comparable between groups, but multiple valvular heart disease was more common in patients with Heyde syndrome (5 patients [27.8%] vs 0 patients).^34^ Among patients with Heyde syndrome, 9 required a median (IQR) of 5.0 (2.0-7.5) transfusion units in the 6 months prior to study inclusion. Other patients were not transfusion dependent. Patients with Heyde syndrome had lower hemoglobin, angiogenesis growth factor, and normalized VWF-HMWM levels. The proportion of patients with AVWD was comparable between patients with and without Heyde syndrome.

**Table 1.** Patient Baseline Characteristics (N = 24)

Characteristic	Patients, No. (%)	
	Heyde syndrome (n = 18)	No Heyde syndrome (n = 6)^a^
Age, mean (SD), y	76.8 (7.7)	79.2 (4.7)
Sex		
Female	5 (27.8)	1 (16.7)
Male	13 (72.2)	5 (83.3)
Comorbidities		
Any	14 (77.8)	6 (100)
Coronary artery disease	9 (50.0)	5 (83.3)
Chronic renal failure	7 (38.9)	0
Coagulopathies^b^	4 (22.2)	0
Diabetes	9 (50.0)	4 (66.7)
Multiple valvular heart disease		
Any	5 (27.8)	0
Mitral regurgitation	3 (16.7)	0
Tricuspid regurgitation	2 (11.1)	0
Pulmonary regurgitation	2 (11.1)	0
Aortic echocardiography findings, mean (SD)		
Left ventricular ejection fraction, %	50.7 (10.3)	47.7 (14.2)
Peak aortic velocity, m/s	3.7 (1.2)	3.8 (0.4)
Peak aortic valve gradient, mm Hg	60.7 (25.7)	58.8 (11.7)
Mean aortic valve gradient, mm Hg	34.0 (14.9)	34.8 (7.6)
Aortic valve area, cm^2^	0.9 (0.2)	0.9 (0.1)
Use of antithrombotics^c^		
Any	18 (100)	6 (100)
Antiplatelets	9 (50.0)	3 (50.0)
Anticoagulants	9 (50.0)	3 (50.0)
Previous angiodysplasia diagnosis^d^	4 (22.2)	0
Required transfusion units^e^		
Yes	9 (50.0)	0
Transfusion units received, median (IQR), No.^e^	5.0 (2.0-7.5)	NA
Laboratory results, mean (SD)		
Hemoglobin, g/dL	10.8 (1.8)	11.4 (1.3)
Angiogenesis growth factors, pg/mL		
Vascular endothelial growth factor	80.2 (33.6)	47.4 (11.3)
Angiopoietin-2	555.5 (225.0-3035.0)	471.6 (4.2-1105.5)
Von Willebrand factor analyses		
VWF:Ag, median (IQR), IU/dL	157.4 (125.0-209.1)	131.0 (86.3-207.0)
VWF:RCoF, median (IQR), IU/dL	143 (109.6-191.3)	133.0 (99.0-176.8)
VWF:RCoF/VWF:Ag, ratio	0.9 (0.1)	1.0 (0.2)
VWF:RCoF/VWF:Ag <0.7	1 (5.6)	0
VWF-HMWM		
Mean (SD), %	46.3 (5.9)	47.2 (4.8)
Normalized, mean (SD), %	91.7 (14.2)	95.8 (12.3)
Normalized <80%	3 (16.7)	1 (16.7)
Vascular lesions, mean (SD), No.		
Any	5.3 (6.0)	NA
Typical angiodysplasias	2.6 (3.6)	NA
Diminutive angiectasias, median (IQR)	1.0 (0.0-2.0)	NA
Active bleeding, median (IQR)	0.0 (0.0-1.0)	NA

Abbreviations: NA, not applicable; VWF, von Willebrand factor; VWF:Ag, VWF antigen; VWF-HMWM, VWF high–molecular weight multimers; VWF:RCoF, VWF ristocetin cofactor or activity.

SI conversion factor: To convert hemoglobin to g/L, multiply by 10.

^a^ Of 6 patients without Heyde syndrome, 4 were diagnosed with different pathologies (2 patients with duodenal ulcers, 1 patient with duodenal adenocarcinoma, and 1 patient with fundic gland polyps).

^b^ Coagulopathies included thrombocytopenia (2 patients) and thrombocytopathy secondary to myelodysplastic syndrome (2 patients). Both myelodysplastic syndrome patients had platelet counts within reference range, were categorized as low risk, and received darbepoetin treatment for multiple years before study participation.

^c^ Two patients with Heyde syndrome used dual antiplatelet therapy before TAVI. Three patients with Heyde syndrome used vitamin K antagonists instead of direct oral anticoagulants.

^d^ Four patients had been previously diagnosed with angiodysplasias through bidirectional endoscopy (3 patients) or capsule endoscopy (1 patient).

^e^ Includes all patients who required at least 1 red blood cell transfusion or intravenous iron infusion (transfusion units) in the 6 months prior to study inclusion.

In total, 15 patients with Heyde syndrome underwent a TAVI procedure; 3 patients were ineligible due to comorbidities (Figure 1). Eleven patients agreed to undergo a second capsule endoscopy 6 months after TAVI (mean [SD], 208 [31] days). Patients had not received endoscopic treatment or antiangiogenic agents between TAVI and the second capsule endoscopy, and the antithrombotic regimen was not modified. Capsule endoscopy assessments of both readers (κ = 0.62) are detailed in eTable 3 in Supplement 1.

### Primary Outcome: Differences in Vascular Lesions

**Figure 2.**
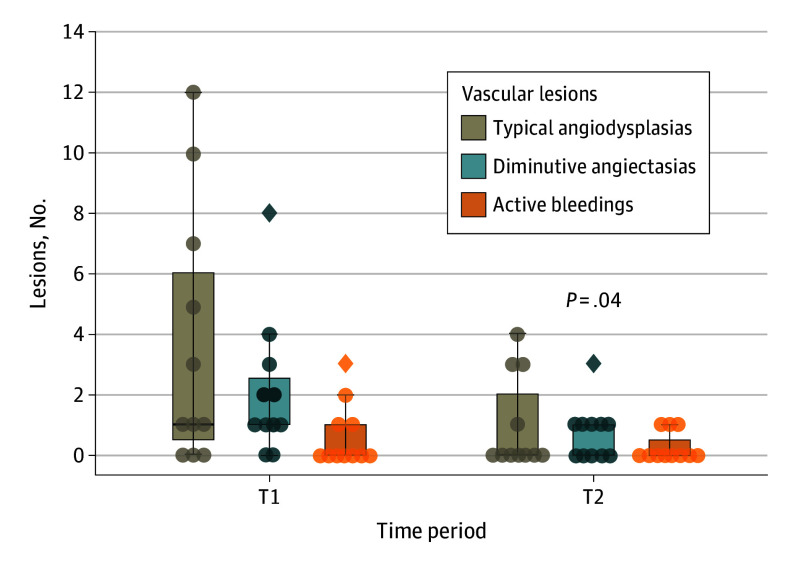
Differences in Vascular Lesions Before vs After Transcatheter Aortic Valve Implantation T1 indicates before transcatheter aortic valve implantation; T2, 6 months after transcatheter aortic valve implantation. Boxes indicate IQR; horizontal lines, medians; whiskers, range; diamonds, outliers; and circles, individual patients.

Patients with Heyde syndrome who underwent a second capsule endoscopy had a mean (SD) of 6.4 (5.6) vascular lesions each before TAVI. Vascular lesions included typical angiodysplasias (median [IQR], 1.0 [0.0-7.0] lesions per patient), diminutive angiectasias (mean [SD], 2.1 [2.3] lesions per patient), and active bleedings (median [IQR], 0.0 [0.0-1.0] lesions per patient). Six months after TAVI, the number of vascular lesions decreased to a mean (SD) of 2.0 (2.1) lesions per patient, a significant reduction of 4.4 (95% CI, 0.4-8.4) lesions (*P* = .04). All types of vascular lesions decreased numerically after TAVI (Figure 2 and Table 2). Of 11 patients who underwent the second capsule endoscopy, 9 patients (81.8%) had fewer vascular lesions after TAVI. Only 1 patient had more vascular lesions after TAVI, and another retained the same number of lesions (1 active bleeding lesion in the colon). Typical angiodysplasias disappeared in 6 patients (54.5%), including 2 patients who each retained 1 diminutive angiectasia and 4 patients without vascular lesions. All vascular lesions are illustrated in eFigure 2 in Supplement 1.

**Table 2.** Outcomes After TAVI Among 11 Patients Who Underwent a Second Capsule Endoscopy

Characteristic	TAVI		Difference (95% CI)	*P* value
	Before	After		
Vascular lesions, mean (SD), No.	6.4 (5.6)	2.0 (2.1)	4.4 (0.4 to 8.4)	.04
Typical angiodysplasias, median (IQR), No.^a^	1.0 (0.0 to 7.0)	0.0 (0.0 to 3.0)	1.0 (−0.8 to 2.8)	.03
Diminutive angioectasias, No.	2.1 (2.3)	0.7 (0.9)	1.4 (−0.3 to 3.0)	.10
Active bleedings, median (IQR), No.^a^	0.0 (0.0 to 1.0)	0.0 (0.0 to 1.0)	0.0 (0.0 to 0.0)	.29
Secondary outcomes, mean (SD)				
Hemoglobin, g/dL	11.4 (1.5)	12.1 (192)	0.6 (−0.3 to 1.5)	.41
Angiogenesis growth factors, pg/mL				
Vascular endothelial growth factor, mean (SD)	88.9 (29.6)	69.6 (24.3)	19.3 (6.3 to 32.3)	.008
Angiopoietin-2, median (IQR)^a^	313 (207 to 764)	258 (120 to 382)	87 (−38 to 212)	.05
VWF				
VWF:Ag, mean (SD), IU/dL	148.2 (43.3)	160.2 (48.0)	12.1 (2.2 to 26.3)	.09
VWF:RCoF, mean (SD), IU/dL	130.6 (36.4)	152.6 (42.6)	22.0 (0.2 to 44.2)	.05
VWF:RCoF/VWF:Ag, median (IQR), ratio^a^	0.9 (0.9 to 1.0)	1.0 (0.9 to 1.0)	0.1 (−0.0 to 0.1)	.06
VWF-HMWM, mean (SD), %	47.3 (6.1)	49.0 (5.5)	1.7 (−2.9 to 6.3)	.44
Normalized VWF-HMWM, mean (SD), %	94.4 (12.2)	98.3 (10.4)	4.6 (−4.8 to 12.4)	.35

Abbreviations: TAVI, transcatheter aortic valve implantation; VWF, von Willebrand factor; VWF:Ag, VWF antigen; VWF-HMWM, VWF high–molecular weight multimers; VWF:RCoF, VWF ristocetin cofactor or activity.

SI conversion factor: To convert hemoglobin to g/L, multiply by 10.

^a^ The Wilcoxon signed-rank test was used instead of the paired samples *t* test.

### Secondary Outcomes

#### Transfusion Requirements

Of 9 patients with Heyde syndrome who required transfusion units in the 6 months before study inclusion (50.0%), 6 underwent TAVI. In the 6 months following TAVI, 12 of 15 patients with Heyde syndrome (80.0%) did not require any transfusions, including 5 who had previously needed them. Three patients required transfusions after TAVI, including 2 who had not needed them previously. One of these patients received transfusions exclusively in the periprocedural period (<48 hours after TAVI). The 3 patients who did not undergo TAVI remained transfusion dependent. Individual transfusion requirements are depicted in eFigure 3 in Supplement 1.

#### Laboratory Analyses

**Figure 3.**
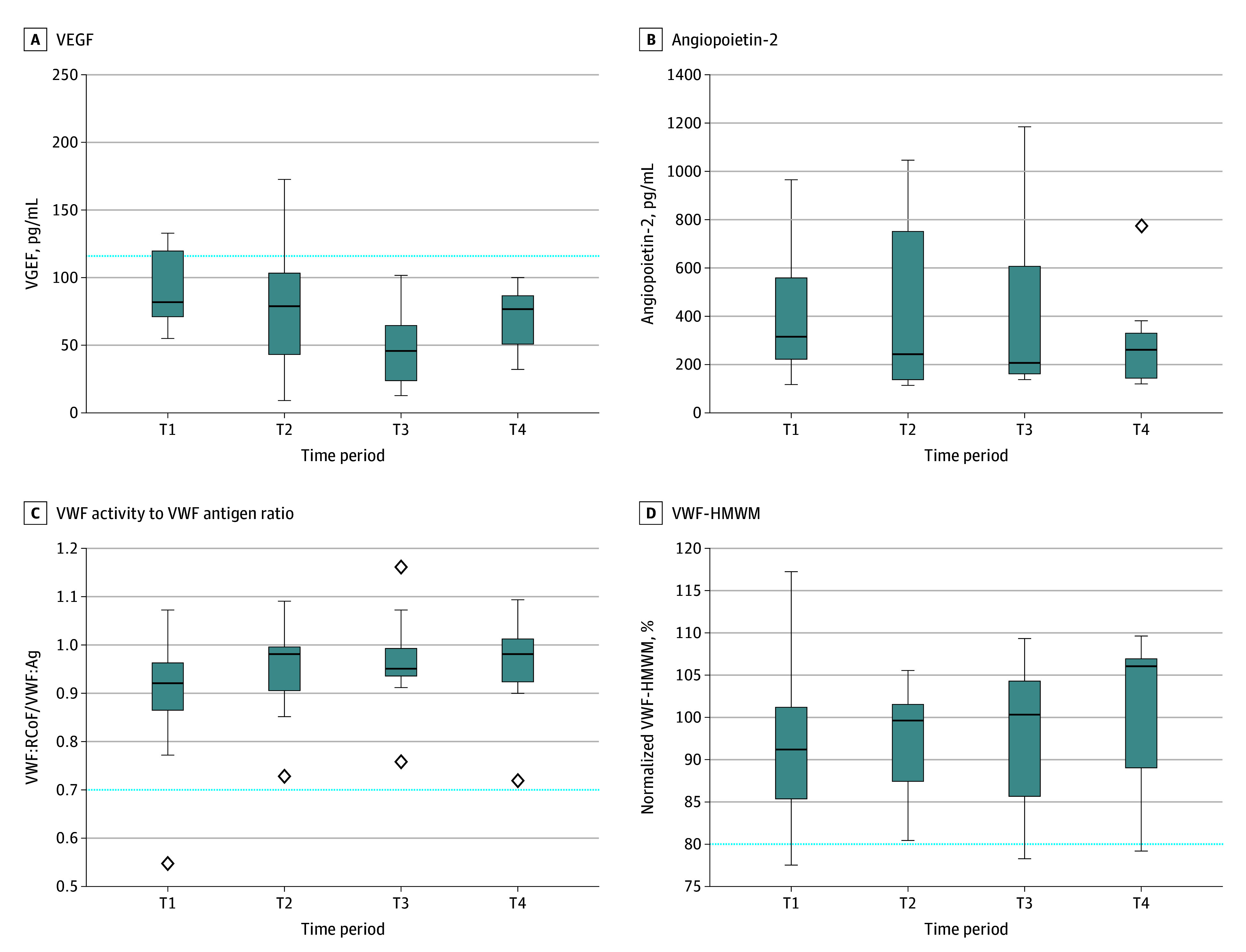
Differences in Laboratory Values Before vs After Transcatheter Aortic Valve Implantation T1 indicates before transcatheter aortic valve implantation; T2, <72 hours after transcatheter aortic valve implantation; T3, 3 months after transcatheter aortic valve implantation; T4, 6 months after transcatheter aortic valve implantation; VEGF, vascular endothelial growth factor; VWF, von Willebrand factor; VWF:Ag, VWF antigen; VWF-HMWM, high–molecular weight multimers; VWF:RCoF, VWF ristocetin cofactor. Boxes indicate IQRs; horizontal lines, medians; whiskers, range; diamonds, outliers; dotted lines, reference range (A) or thresholds to diagnose acquired von Willebrand disease (C and D). The results of 1 patient are not depicted in B, as their levels far exceeded those of the other patients (30 341 pg/mL at T1, 23 213 pg/mL at T2, 21 503 pg/mL at T3, and 16 520 pg/mL at T4).

Table 2 depicts the outcomes of our laboratory analyses before and 6 months after TAVI for 11 patients who underwent the second capsule endoscopy. Figure 3 displays the distribution of angiogenesis growth factors and VWF analyses at every time point. Patients with Heyde syndrome had a mean (SD) hemoglobin level of 11.4 (1.5) g/dL before TAVI, which increased numerically to 12.1 (1.9) g/dL (*P* = .41). VEGF levels significantly decreased after TAVI, from a mean (SD) of 89 (30) pg/mL to 70 (24) pg/mL (*P* = .008), as did angiopoietin-2 levels, from a median (IQR) of 313 (207-764) pg/mL to 258 (120-382) pg/mL (*P* = .05), and all values were within reference ranges at 3- and 6-month follow-ups (Figure 2A and B). The protein levels of VWF (VWF:Ag) and VWF activity (VWF:RCoF) remained within reference ranges (50 to 178 IU/dL) throughout the study period. Overall, there was no significant change in VWF:Ag levels before vs after TAVI (mean [SD], 148.2 [43.3] IU/dL vs 160.2 [48.0] IU/dL; *P* = .09) but VWF:RCoF levels significantly increased (mean [SD], 130.6 [36.4] IU/dL vs 152.6 [42.6] IU/dL; *P* = .05). The normalized proportion of VWF-HMWM remained unchanged before and after TAVI (mean [SD], 94.4% [12.2%] vs 98.3% [10.4%]; *P* = .44). Before TAVI, only 2 patients (18.2%) had AVWD (Figure 2C and D). After TAVI, both patients fully recovered, while 1 patient (9.1%) was newly diagnosed with AVWD. Densitometry plots of the VWF multimer distribution are illustrated in eFigure 4 in Supplement 1.

#### Factors Associated With Angiodysplasia Recovery

All patients with multiple valvular heart disease before TAVI still had typical angiodysplasias at follow-up (3 of 3 patients), while of 8 patients with isolated AS, 6 patients (75.0%) did not have typical angiodysplasias after TAVI. Patients with mild or worse paravalvular leakage after TAVI were more likely to have angiodysplasias at follow-up (3 of 5 patients [60.0%]) compared with patients with no or trace paravalvular leakage (2 of 6 patients [33.3%]). The only patient with AVWD after TAVI had no typical angiodysplasias at follow-up (eTable 4 in Supplement 1).

## Discussion

In this prospective cohort study, we examined whether TAVI was associated with gastrointestinal vascular lesions in patients with Heyde syndrome, and we were able to make several key observations. First, TAVI was associated with a significantly reduced mean number of vascular lesions (from 6.4 to 2.0) per patient, and 55% of patients no longer had typical angiodysplasias after TAVI. Second, the reduction in vascular lesions coincided with a significant decline in angiogenesis growth factors. Third, angiodysplasia recovery was not associated with VWF improvements, as most patients (78%) commenced the study with levels within reference ranges. Fourth, patients with persistent angiodysplasias after TAVI were more likely to have residual heart valve disease. These findings underscore the efficacy of TAVI as a treatment modality for patients with Heyde syndrome.

We endoscopically reassessed patients with Heyde syndrome 6 months after TAVI and found that the mean number of vascular lesions significantly decreased, from 6.4 to 2.0. This finding aligns with the results reported by Yashige et al,^18^ who found a reduction in angiodysplasias from 9.0 to 4.0. Typical angiodysplasias were no longer present in 6 of 11 patients evaluated (55%). Our previous meta-analysis of 10 retrospective cohorts (including 300 patients) reported a pooled gastrointestinal bleeding cessation rate of 73% after aortic valve replacement.^14^ Several factors can explain the lower rate observed in the current study. First, bleeding cessation might precede complete angiodysplasia resolution, as lesion size has been associated with active bleeding.^38^ The study by Yashige et al^18^ found that persisting angiodysplasias after TAVI had reduced in size and were no longer actively bleeding, which was also observed in 2 patients in our study. Second, our aforementioned meta-analysis found lower bleeding cessation rates after TAVI compared with surgical valve replacement (82% vs 64%).^14^ The higher rate of residual valve disease following TAVI could explain this difference, as continuous bleeding was more common among patients with paravalvular leakage.^11,12,14^ This accords with the current findings, as patients with multiple valvular heart disease before TAVI and paravalvular leakage after TAVI were more likely to have persistent angiodysplasias at follow-up. Third, the retrospective design of earlier studies could have introduced bias with overestimation of results.^14^

Angiodysplasias have long been recognized as the probable source of gastrointestinal bleeding in Heyde syndrome.^5^ However, the reported prevalence in patients with AS varies considerably, from 1 to 20%.^14,32^ We found that of 24 patients with severe AS and iron-deficiency anemia, 18 had Heyde syndrome, which supports the prevalence of 94% reported by Yashige et al.^18^ Earlier studies likely underdiagnosed angiodysplasias, as capsule endoscopy is not routinely performed in patients with symptomatic anemia, causing small bowel lesions (78% of angiodysplasias) to go unnoticed.^14,16^ Moreover, we detected angiodysplasias in patients with asymptomatic anemia who would not have been assessed otherwise. We found that the VWF levels of patients with Heyde syndrome were largely within reference ranges, corroborating previous reports.^18,39^ Only 4 of 18 patients with Heyde syndrome (22%) were diagnosed with AVWD, disputing the hypothesis that VWF is central to the angiogenesis-dependent formation of angiodysplasias.^8,14^ Similarly, AVWD was also diagnosed in 2 patients without vascular lesions (1 patient before and 1 patient after TAVI). However, AVWD likely contributes to bleeding severity, as all patients with a normalized VWF-HMWM proportion less than 80% experienced symptomatic bleeding episodes before TAVI, despite having relatively few vascular lesions. Supporting this hypothesis, a prior study reported angiodysplasias in 50% of heart failure patients before left ventricular assist device insertion and subsequent AVWD and gastrointestinal bleeding.^40,41^

Multiple studies have highlighted the prognostic impact of anemia in patients undergoing TAVI, showing worse clinical outcomes.^1,2^ Endoscopic argon plasma coagulation is the first-line treatment of angiodysplasias, but high rebleeding rates have been reported due to lesion recurrence.^42^ Second-line treatment includes antiangiogenic agents (octreotide and thalidomide).^43,44,45^ Similar to TAVI, octreotide decreases the number and size of angiodysplasias, reducing bleeding episodes.^43,46,47^ Despite promising results, medication is expensive, and discontinuation leads to rebleeding.^15,48,49^ Notably, we found that angiogenesis growth factor levels (VEGF and angiopoietin-2) were significantly reduced up to 6 months after TAVI, suggesting an advantage in managing angiodysplasias. However, as complete angiodysplasia resolution takes several months, bleeding cessation might not immediately follow TAVI.^18^ Indeed, a 2022 study noted a surge in gastrointestinal bleeding in the first months after aortic valve repair, possibly due to antithrombotics.^11^ In the current study, 2 patients also continued to experience gastrointestinal bleeding despite a noticeable decrease in angiodysplasias. Conventional treatment modalities could bridge this gap, as numerous reports demonstrated reduced bleeding rates in patients with Heyde syndrome who received prophylactic care.^22,39,50,51^

### Limitations

Our study has several limitations, including the lack of a control group, allowing secular changes to influence results. As angiodysplasias tend to increase with age, this could have led to an underestimation of effects.^38^ To minimize bias in the pre-post design, patients were not allowed to receive endoscopic treatment or antiangiogenic agents during the study.^15,43^

Additional limitations include the small sample size and the relatively high number of patients who did not undergo TAVI or a second capsule endoscopy. Notably, the 3 patients excluded from TAVI all had frequent bleeding episodes, 2 of whom had AVWD, highlighting the frailty and complex medical status of patients with Heyde syndrome. Despite these issues, our results are consistent with a 2023 endoscopy study.^18^

Furthermore, the relatively low number of patients with symptomatic bleeding episodes before TAVI obscures clinical relevance and may explain the low prevalence of AVWD.^18,39^ The low prevalence could also be due to the use of a semiautomated VWF-multimer assay, which has not been validated specifically for structural heart disease.^29^ However, this assay has been validated for type 2A and 2B von Willebrand disease, which are also characterized by reduced VWF-HMWM.^29,31^ Additionally, it was developed to minimize high error rates associated with nonstandardized, in-house techniques, likely accounting for the wide variation in reported AVWD prevalence among patients with severe AS.^14,30^ Moreover, we also determined the VWF:RCoF to VWF:Ag ratio, a common surrogate marker for VWF-HMWM in structural heart disease, which indicated an even lower AVWD prevalence.^14^

Furthermore, most patients did not undergo bidirectional endoscopy at the time of capsule endoscopy, despite its superiority in diagnosing stomach and colon lesions.^16^ Nevertheless, capsule endoscopy results were consistent with available bidirectional endoscopy, and the proportion of patients with angiodysplasias in the stomach (22%) and colon (50%) accords with previous literature.^6,18^ Additionally, while capsule endoscopy has a high diagnostic yield for small bowel angiodysplasias, minute vascular lesions may have been missed.^52^

## Conclusions

In this cohort study of 24 patients with iron deficiency anemia and severe AS, Heyde syndrome was identified as the cause of anemia in 75% of patients. Vascular lesions decreased significantly after TAVI, suggesting that it is an effective treatment for angiodysplasia-related bleeding. However, paravalvular leakage and concomitant valvular heart disease prevented the complete resolution of angiodysplasias.

## Notes

Supplement 1. **eFigure 1.** Study Design **eTable 1.** Description of Vascular Lesions **eTable 2.** Laboratory Analyses **eTable 3.** Capsule Endoscopy Assessment **eFigure 2.** Capsule Endoscopy Images of Vascular Lesions **eFigure 3.** Transfusion Requirements of Patients With Heyde Syndrome **eFigure 4.** Densitometry Plots of the von Willebrand Factor Multimer Distribution **eTable 4.** Factors Associated With Angiodysplasia Resolution After TAVI
eReferences.

Supplement 2.
Data Sharing Statement
