# Supplementary material for: Gastrointestinal Angiodysplasia Resolution After Transcatheter Aortic Valve Implantation
Source: JAMA Netw Open. 2024 Oct 30;7(10):e2442324. doi: 10.1001/jamanetworkopen.2024.42324 (PMC11525602; doi:10.1001/jamanetworkopen.2024.42324)
Supplement: Supplement 1. — eFigure 1. Study Design eTable 1. Description of Vascular Lesions eTable 2. Laboratory Analyses eTable 3. Capsule Endoscopy Assessment eFigure 2. Capsule Endoscopy Images of Vascular Lesions eFigure 3. Transfusion Requirements of Patients With Heyde Syndrome eFigure 4. Densitometry Plots of the von Willebrand Factor Multimer Distribution eTable 4. Factors Associated With Angiodysplasia Resolution After TAVI eReferences. [file jamanetwopen-e2442324-s001.pdf]

Supplemental Online Content

Goltstein LMJ, Rooijakkers MP, Thierens NE, et al. Gastrointestinal angiodysplasia resolution after transcatheter aortic valve implantation. *JAMA Netw Open*. 2024;7(10):e2442324. doi:10.1001/jamanetworkopen.2024.42324

eFigure 1. Study Design

eTable 1. Description of Vascular Lesions

eTable 2. Laboratory Analyses

eTable 3. Capsule Endoscopy Assessment

eFigure 2. Capsule Endoscopy Images of Vascular Lesions

eFigure 3. Transfusion Requirements of Patients With Heyde Syndrome

eFigure 4. Densitometry Plots of the von Willebrand Factor Multimer Distribution

eTable 4. Factors Associated With Angiodysplasia Resolution After TAVI

eReferences

This supplemental material has been provided by the authors to give readers additional information about their work.

© 2024 Goltstein LMJ et al. *JAMA Network Open.*

eFigure 1. Study design

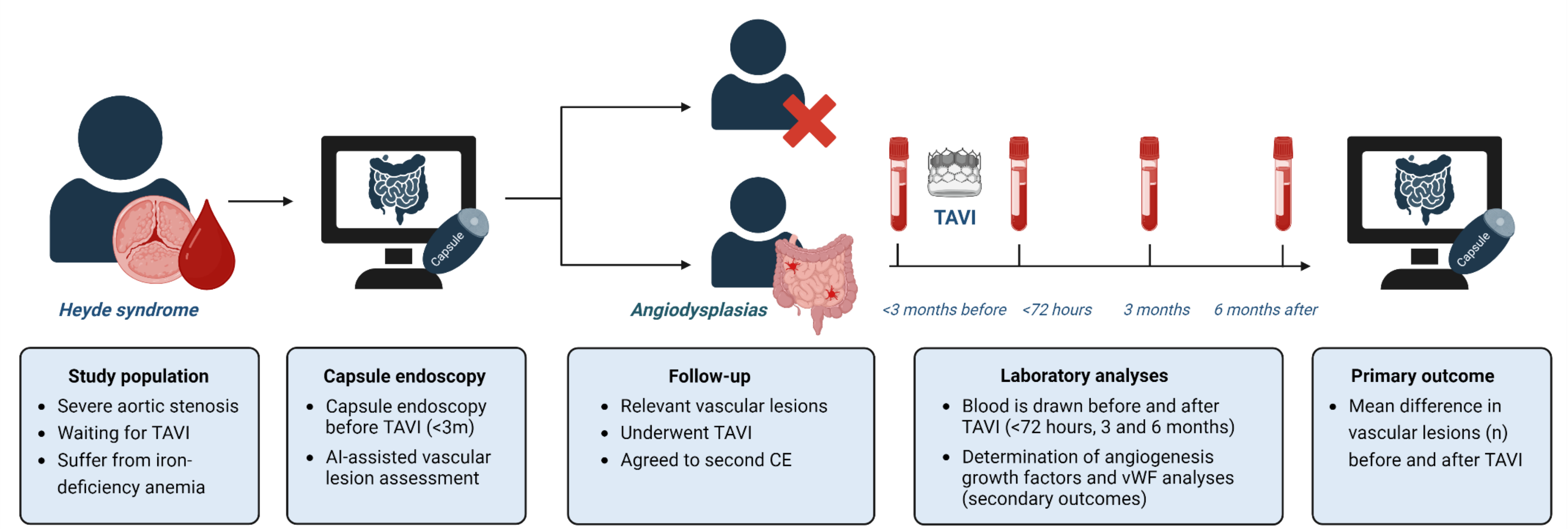

Abbreviations: AI, artificial intelligence; CE, capsule endoscopy; TAVI, transcatheter aortic valve implantation; vWF, von Willebrand factor.
Figure illustrates the design of the study. The study population consisted of all patients with severe aortic stenosis who were waitlisted for TAVI and suffered from iron-deficiency anemia. Capsule endoscopy was performed within three months before undergoing TAVI. All patients with relevant vascular lesions before TAVI were followed up after TAVI. Blood was drawn before TAVI, <72 hours, three months, and six months after TAVI. Patients were asked to undergo a second capsule endoscopy six months after TAVI. The primary outcome was the mean difference in the number of relevant vascular lesions before and after TAVI. Two independent readers assessed vascular lesions using an advanced AI solution (AXARO®, Augmented Endoscopy).[1] Secondary outcomes included the differences in hemoglobin, angiogenesis growth factor, and von Willebrand factor levels.

© 2024 Goltstein LMJ et al. *JAMA Network Open.*

eTable 1. Description of vascular lesions

Nomenclature[b]	Semantic description[b]	Saurin classification[a]
Active bleeding	Lesion unidentifiable due to active bleeding	P2: High bleeding potential
Typical angiodysplasia	A clearly demarcated, bright-red, flat lesion consisting of tortuous and clustered capillary dilatations within the mucosal layer (surrounded by intestinal villi). Can be small (few mm) to large (few cm).	P2: High bleeding potential
Diminutive angiectasia	A clearly demarcated, linear, bright-red lesion consisting of tiny non-clustered capillary dilatations within the mucosal layer (surrounded by intestinal villi).	P1: Intermediate bleeding potential
Erythematous patch	A small (few mm) and flat reddish area without any vessel appearance within the mucosal layer (surrounded by intestinal villi).	P0: Low bleeding potential
Red spot/dot	A minuscule (less than 1 mm), punctuate, and flat lesion with a bright-red area without linear or vessel appearance within the mucosal layer (surrounded by intestinal villi).	P0: Low bleeding potential
Phlebectasia	A small (few mm), flat to slightly elevated, bluish venous dilatation below the mucosa (covered by intestinal villi).	P0: Low bleeding potential

[a]Vascular lesions were divided into lesions with an intermediate (P1) to high risk of bleeding (P2), and lesions with no or a low bleeding potential (P0) according to the Saurin classification.[2]
[b]Relevant vascular lesions (P1 and P2) were classified into diminutive angiectasias (P1) and typical angiodysplasias (P2), following two consensus statements on the semantic description and clinical relevance of vascular lesions.[3,4]

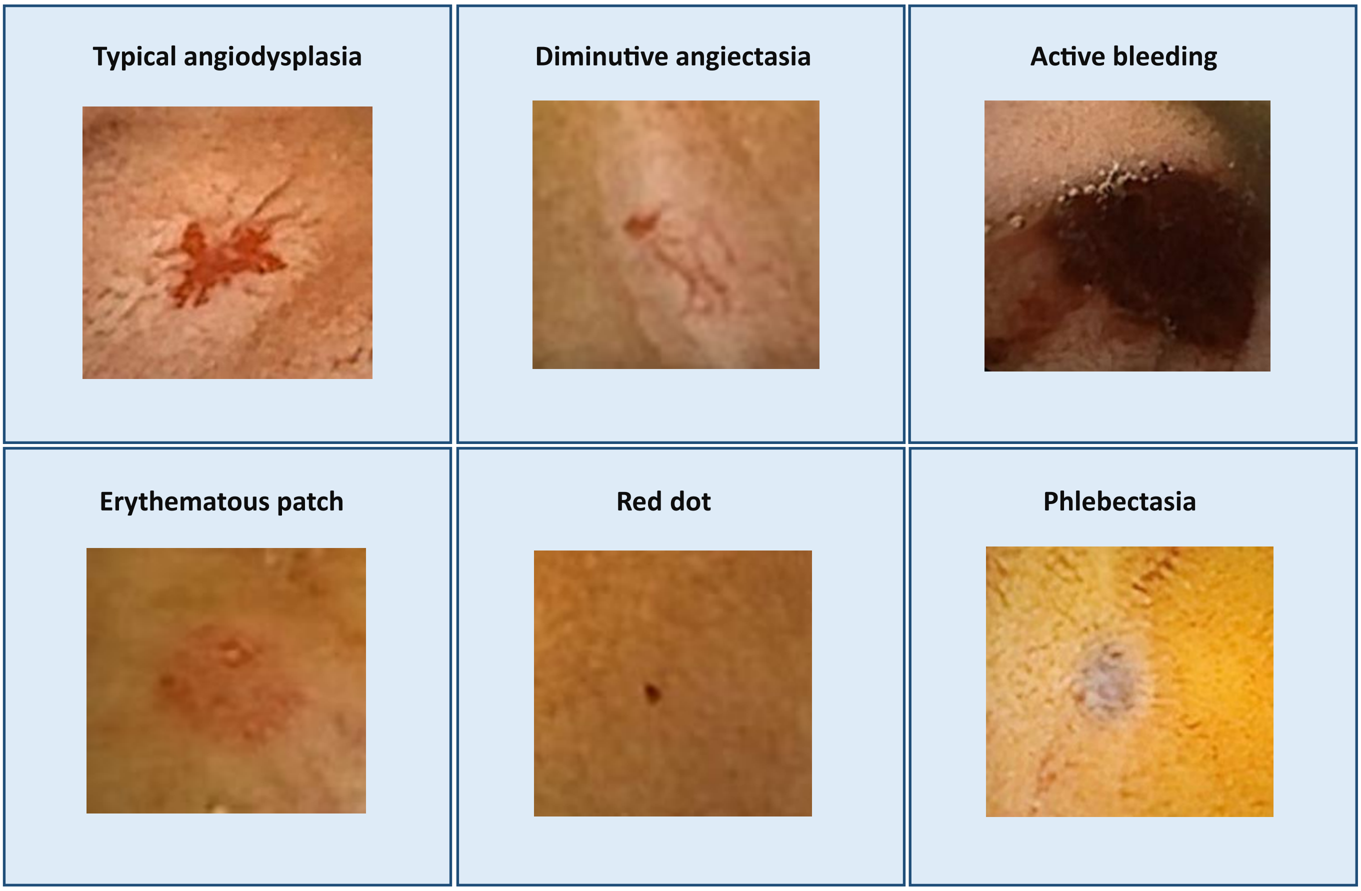

© 2024 Goltstein LMJ et al. *JAMA Network Open.*

**eTable 2**. Laboratory analyses		
**Laboratory test**	**Assay or software program**	**Company**
Vascular endothelial growth factor (VEGF)	Human VEGF Quantikine ELISA Kit DVE00	R&D Systems (Minnesota, USA)
Angiopoietin-2	Human Angiopoietin-2 ELISA Kit ab99971	Abcam (Cambridge, UK)
Von Willebrand factor antigen (vWF:Ag)	CLIA of HemosIL AcuStar	Werfen (Barcelona, Spain)
Ristocetin cofactor (vWF:RCo)	CLIA of HemosIL AcuStar	Werfen (Barcelona, Spain)
Multimeric pattern	Semi-automated Hydrasis2 analyzer	Sebia (Lisses, France)
VWF-HMWM proportion (densitometry)	Phoresis CORE version 9.30.	Sebia (Lisses, France)

Abbreviations: CLIA, chemoluminescent immunoassays; ELISA, enzyme-linked immunosorbent assay; VEGF, vascular endothelial growth factor; vWF, von Willebrand factor; vWF:Ag, vWF antigen; vWF-HMWM, vWF high-molecular-weight multimers; vWF:RCo, vWF Ristocetin cofactor or activity.

© 2024 Goltstein LMJ et al. *JAMA Network Open.*

eTable 3A. Capsule endoscopy assessment

Patient		Preselected images by AXARO[a]	Vascular lesions (P1/P2)		Common lesions[b]	Missing lesions[c]		Additional lesions[c]		Scored differently[b]
			Reader 1	Reader 2		Reader 1	Reader 2	Reader 1	Reader 2	
**Patient 1**	***Baseline***	116	10	5	5	0	3	2	0	0
	Follow-up	129	4(1)	3	3	0	1	0	0	0
**Patient 2**	***Baseline***	38	1	2	1	0	0	0	1	0
**Patient 3**	***Baseline***	659	17	16	15	1	2	0	0	1
	Follow-up	293	4	1	1	0	1	2	0	0
**Patient 4**	***Baseline***	129	2	1	1	0	1	0	0	0
	Follow-up	72	1	1	0	0	0	1	1	0
**Patient 5**	***Baseline***	227	5	3	2	0	0	3	1	0
**Patient 6**	***Baseline***	104	2	1	1	0	0	1	0	0
**Patient 7**	***Baseline***	387	13	12	10	3	4	1	1	2
	Follow-up	185	2	0	0	0	0	2	0	0
**Patient 8**	***Baseline***	88	2	0	0	0	1	1	0	0
	Follow-up	75	6	3	2	0	1	2	0	0
**Patient 9**	***Baseline***	26	3	3	3	0	0	0	0	0
	Follow-up	995	0	0	0	0	0	0	0	0
**Patient 10**	***Baseline***	823	0	0	0	0	0	0	0	0
**Patient 11**	***Baseline***	772	0	0	0	0	0	0	0	0
**Patient 12**	***Baseline***	54	1	3	1	0	0	0	2	0
**Patient 13**	***Baseline***	252	8	4	3	1	3	3	1	0
	Follow-up	468	9	5	5	0	0	4	0	2
**Patient 14**	***Baseline***	179	9	3	3	0	0	6	0	1
	Follow-up	42	5	0	0	0	0	5	0	0
**Patient 15**	***Baseline***	94	7	4	4	0	3	0	0	2
	Follow-up	150	2	1	1	0	0	1	0	0

© 2024 Goltstein LMJ et al. *JAMA Network Open.*

Patient		Preselected images by AXARO[a]	Vascular lesions (P1/P2)		Common lesions[b]	Missing lesions[c]		Additional lesions[c]		Scored differently[b]
			Reader 1	Reader 2		Reader 1	Reader 2	Reader 1	Reader 2	
**Patient 16**	***Baseline***	255	0	1	0	0	0	0	1	0
	[redacted]									
**Patient 17**	***Baseline***	263	2	0	0	0	0	2	0	0
	[redacted]									
**Patient 18**	***Baseline***	58	2	0	0	0	1	1	0	0
	[redacted]									
**Patient 19**	***Baseline***	252	7	19	7	13	1	0	1	0
	[redacted]									
**Patient 20**	***Baseline***	41	2	1	1	0	0	1	0	0
	[redacted]									
**Patient 21**	***Baseline***	242	7	6	6	0	0	1	0	0
	Follow-up	168	5	9	5	0	0	0	4	0
**Patient 22**	***Baseline***	26	0	0	0	0	0	0	0	0
	[redacted]									
**Patient 23**	***Baseline***	71	0	0	0	0	0	0	0	0
	[redacted]									
**Patient 24**	***Baseline***	55	4(1)	1(1)	1	0	0	3	0	1
	Follow-up	82	2	1	1	0	0	1	0	0

[a]Readers were not limited to the preselected images by AXARO®, Augmented Endoscopy.[1] Numbers in brackets depict relevant (P1/P2) vascular lesions only visible on unselected images.[2]
[b]Common lesions refer to all relevant vascular lesions scored by both assessors (as P1/P2 lesions).[2] Readers could have differently categorized relevant vascular lesions (e.g., typical angiodysplasia and diminutive angiectasia).[3-5] Differently categorized relevant vascular lesions are depicted in the last column.
[c]Missing lesions were either not scored by readers or scored as irrelevant (P0) lesions. Additional lesions were scored as relevant vascular lesions (P1/P2) by one assessor before the consensus meeting (where it was found to be irrelevant [P0]).[2]

© 2024 Goltstein LMJ et al. *JAMA Network Open.*

eTable 3B. Overall performance of both readers

		Reader 1		Reader 2	
		**Yes**	**No**	**Yes**	**No**
**AXARO[a]**	**Yes**	101	17	96	22
	**No**	43	7.7	13	7.7
		**Sensitivity**: 85.6%	**Specificity**: 99.4%	**Sensitivity**: 81.4%	**Specificity**: 99.8%

eTable 3C. Interobserver agreement

		Reader 2	
		**Yes**	**No**
**Reader 1**	**Yes**	82	67
	**No**	30	7.7

Interobserver agreement (Kappa) = 0.6

[a]Preselected images by AXARO®, Augmented Endoscopy were set as the total number of cases (with or without relevant vascular lesions) instead of the total number of vascular lesions detected by the readers (relevant [P1/P2] and irrelevant [P0]), because of the large number of P0 lesions that are difficult to distinguish.[5] Readers had access to all images, which allowed for the detection of vascular lesions that were not visible on preselected images. This was the case for 2/179 (1.1%) relevant vascular lesions. The number of differently categorized relevant vascular lesions (P1/P2) is not included in the overall performance (e.g., a relevant vascular lesion classified as a typical angiodysplasia by one assessor and a diminutive angiectasia by the other assessor). This was the case for 9/82 (11.0%) relevant vascular lesions detected by both readers.

© 2024 Goltstein LMJ et al. *JAMA Network Open.*

eFigure 2. Capsule endoscopy images of vascular lesions

Patient 1 - Before TAVI

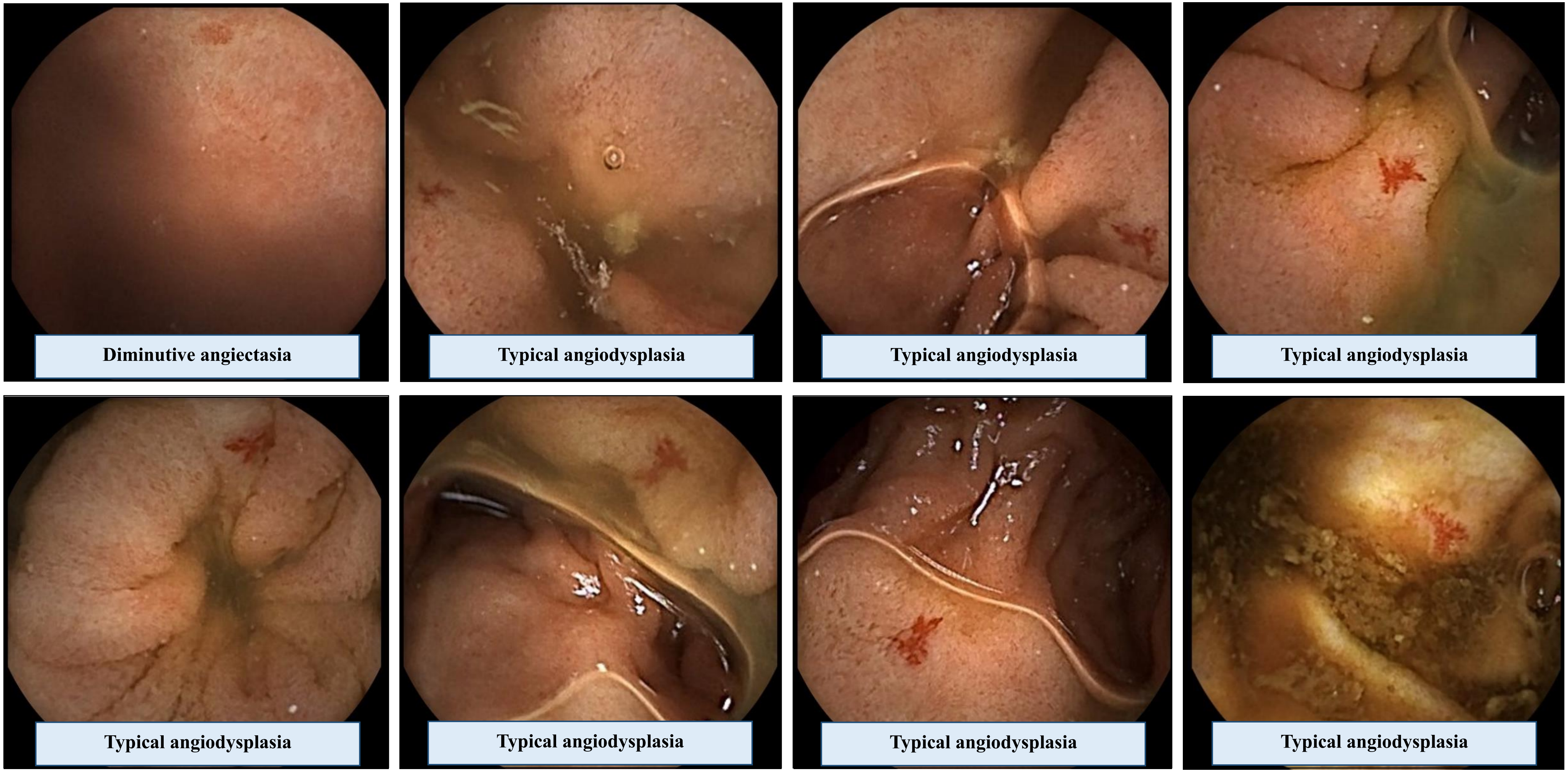

© 2024 Goltstein LMJ et al. *JAMA Network Open.*

Patient 1 - After TAVI

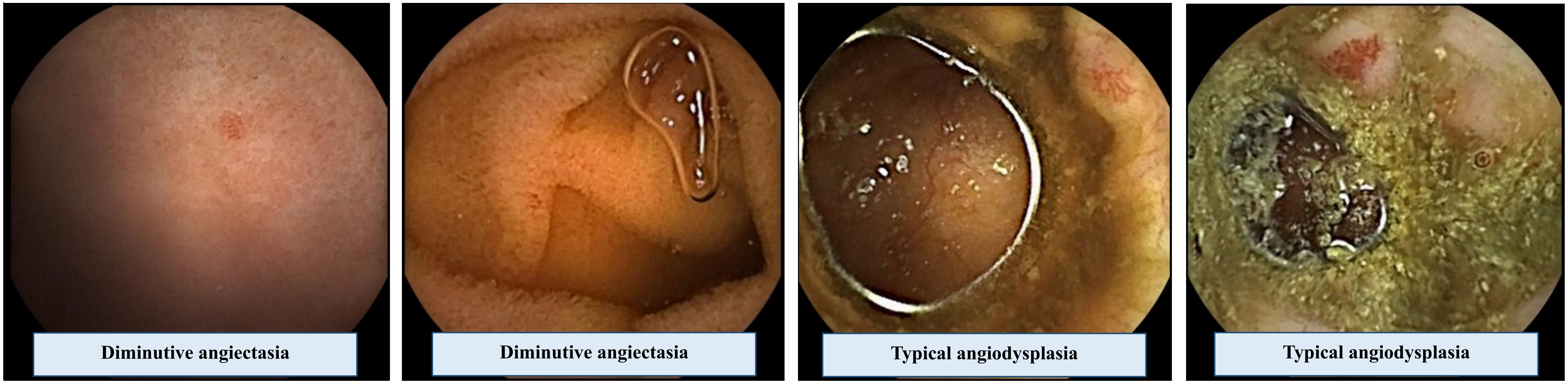

© 2024 Goltstein LMJ et al. *JAMA Network Open.*

Patient 3 - Before TAVI

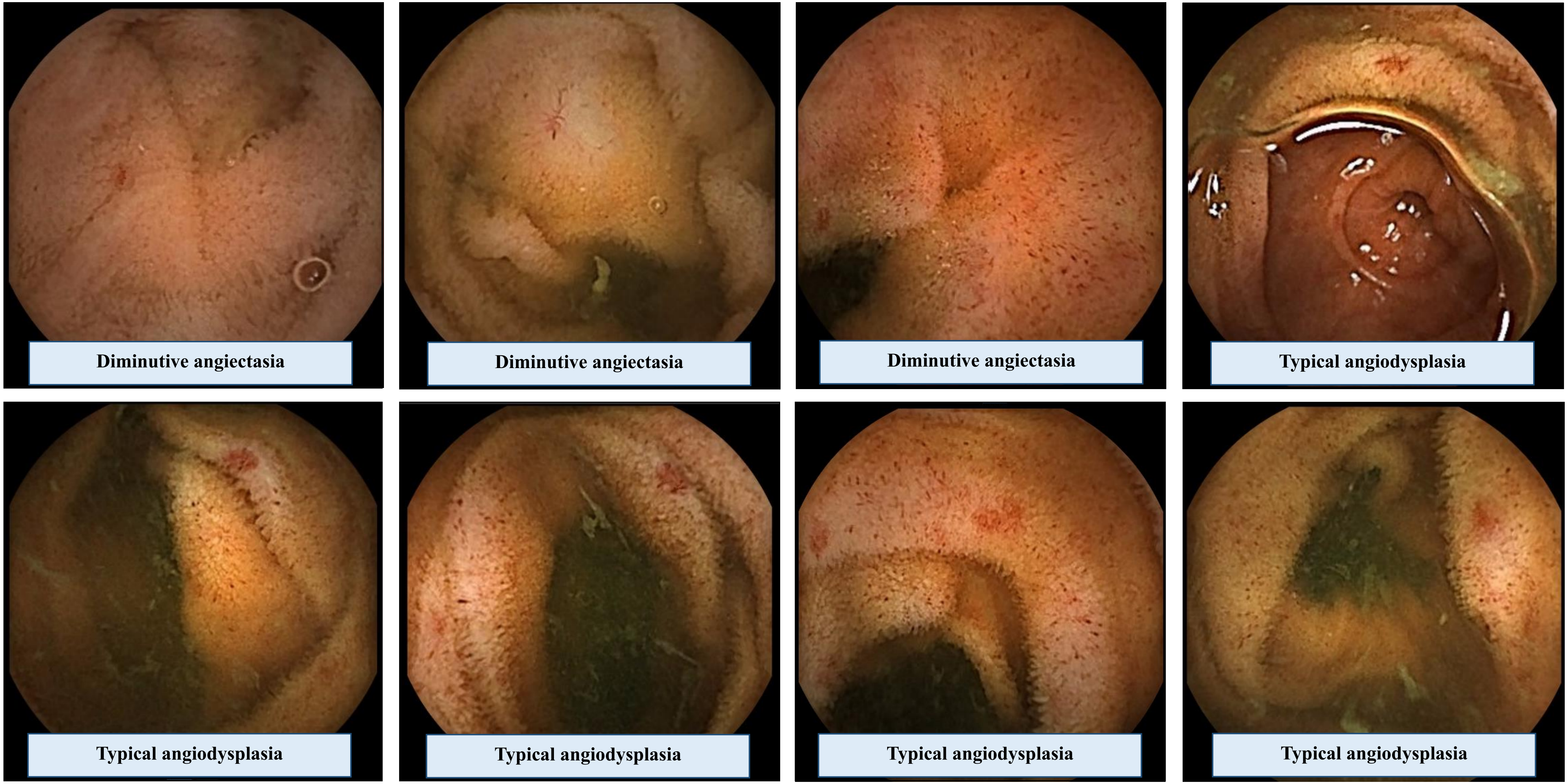

© 2024 Goltstein LMJ et al. *JAMA Network Open.*

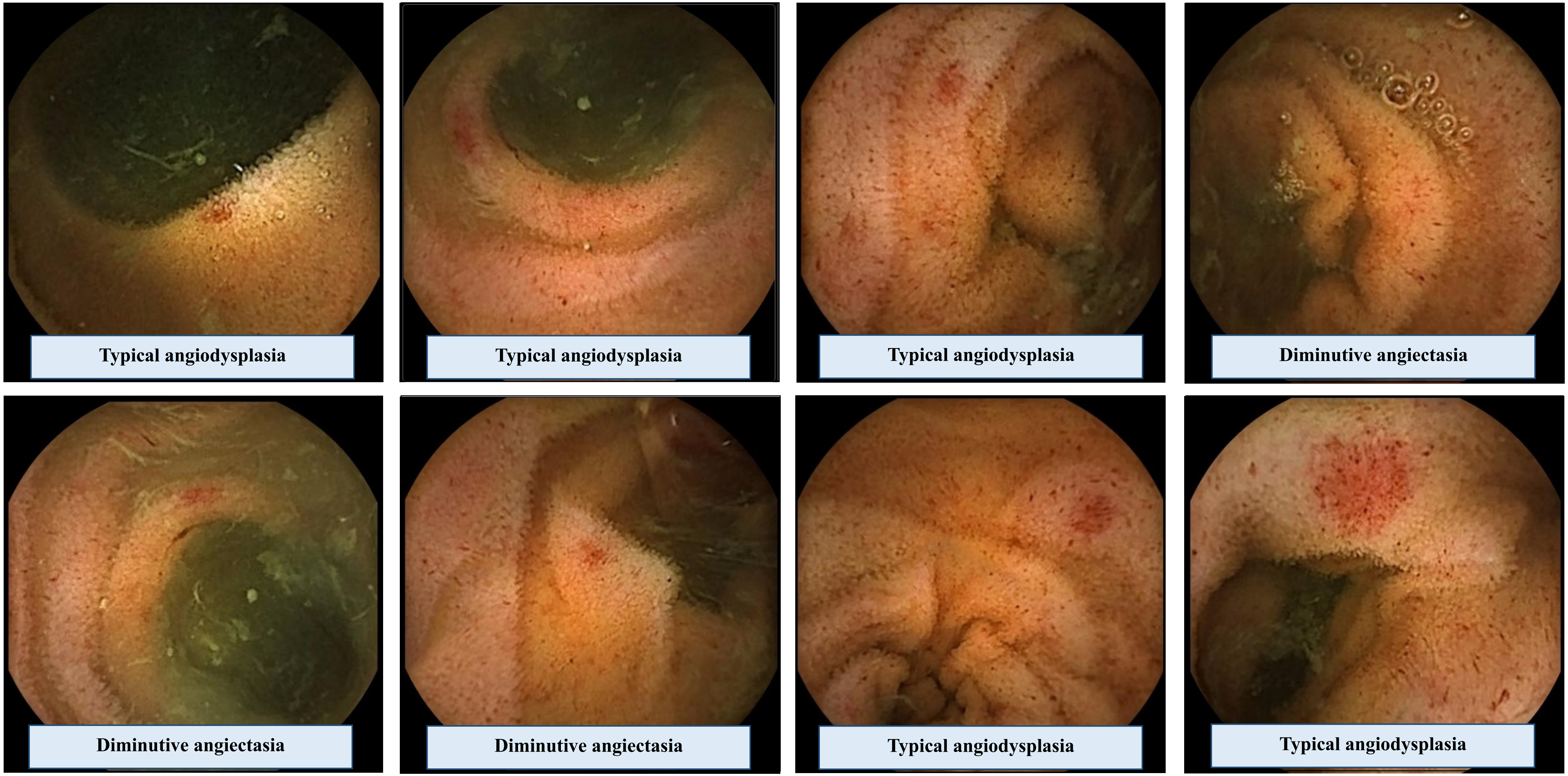

© 2024 Goltstein LMJ et al. *JAMA Network Open.*

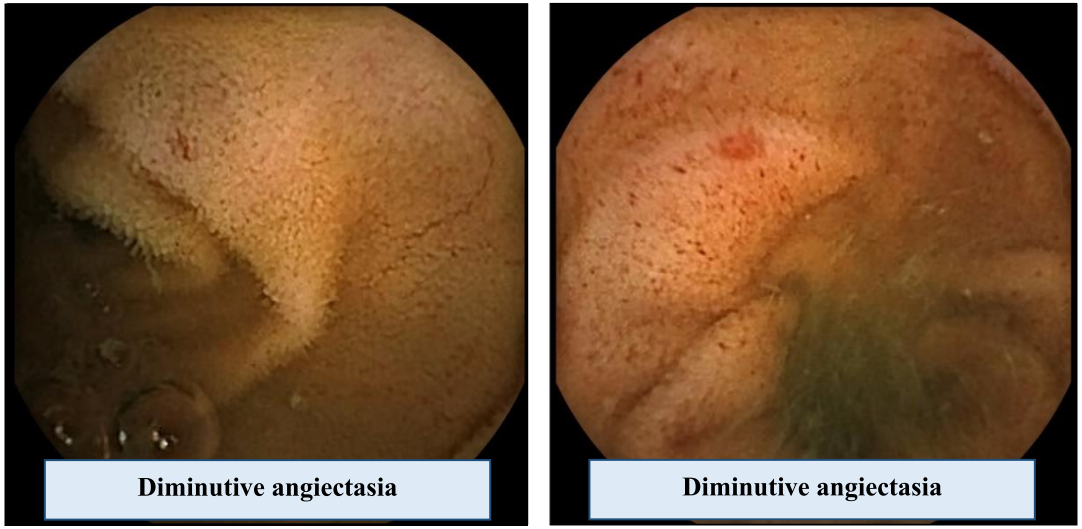

Patient 3 - After TAVI

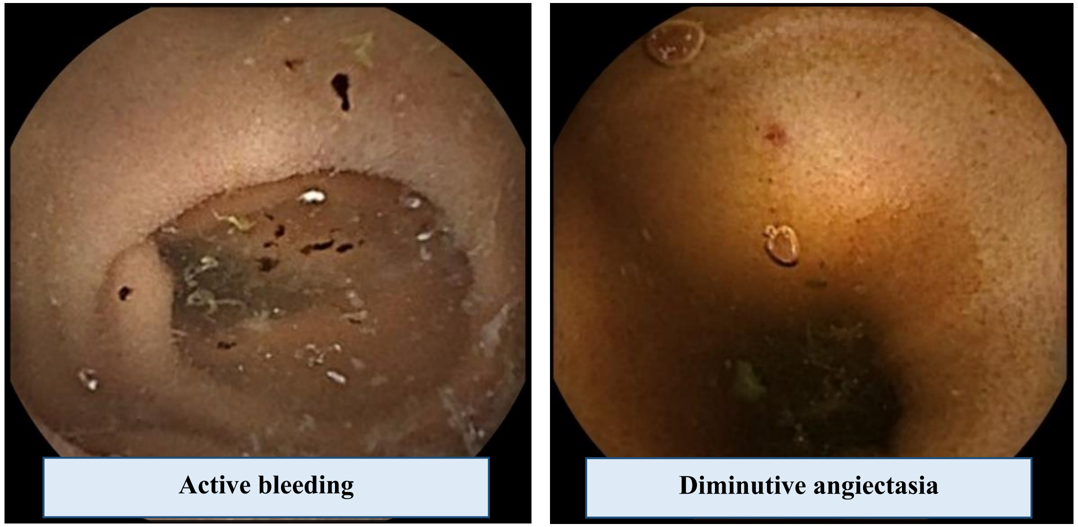

© 2024 Goltstein LMJ et al. *JAMA Network Open.*

Patient 4 - Before TAVI

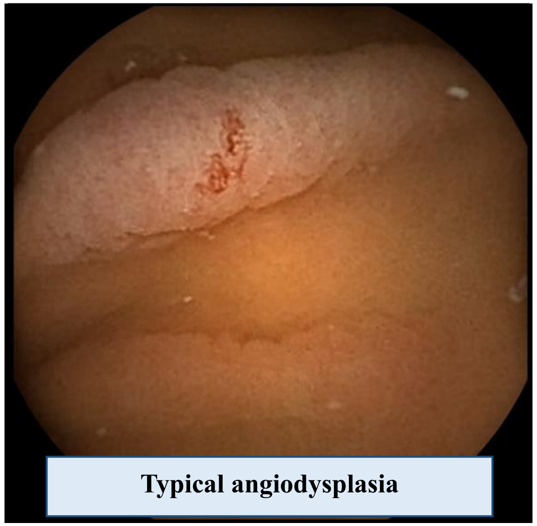

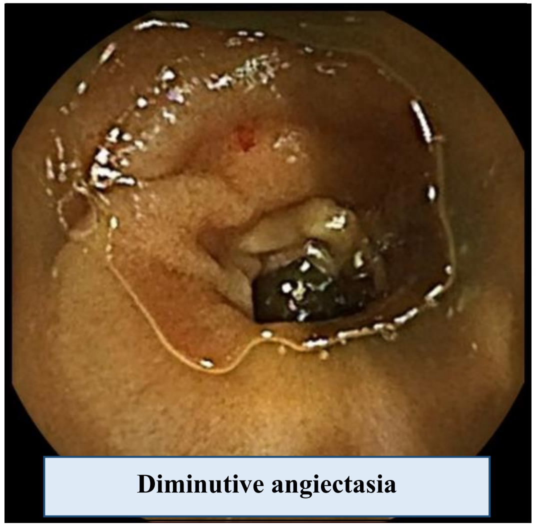

No vascular lesions after TAVI

© 2024 Goltstein LMJ et al. *JAMA Network Open.*

Patient 7 - Before TAVI

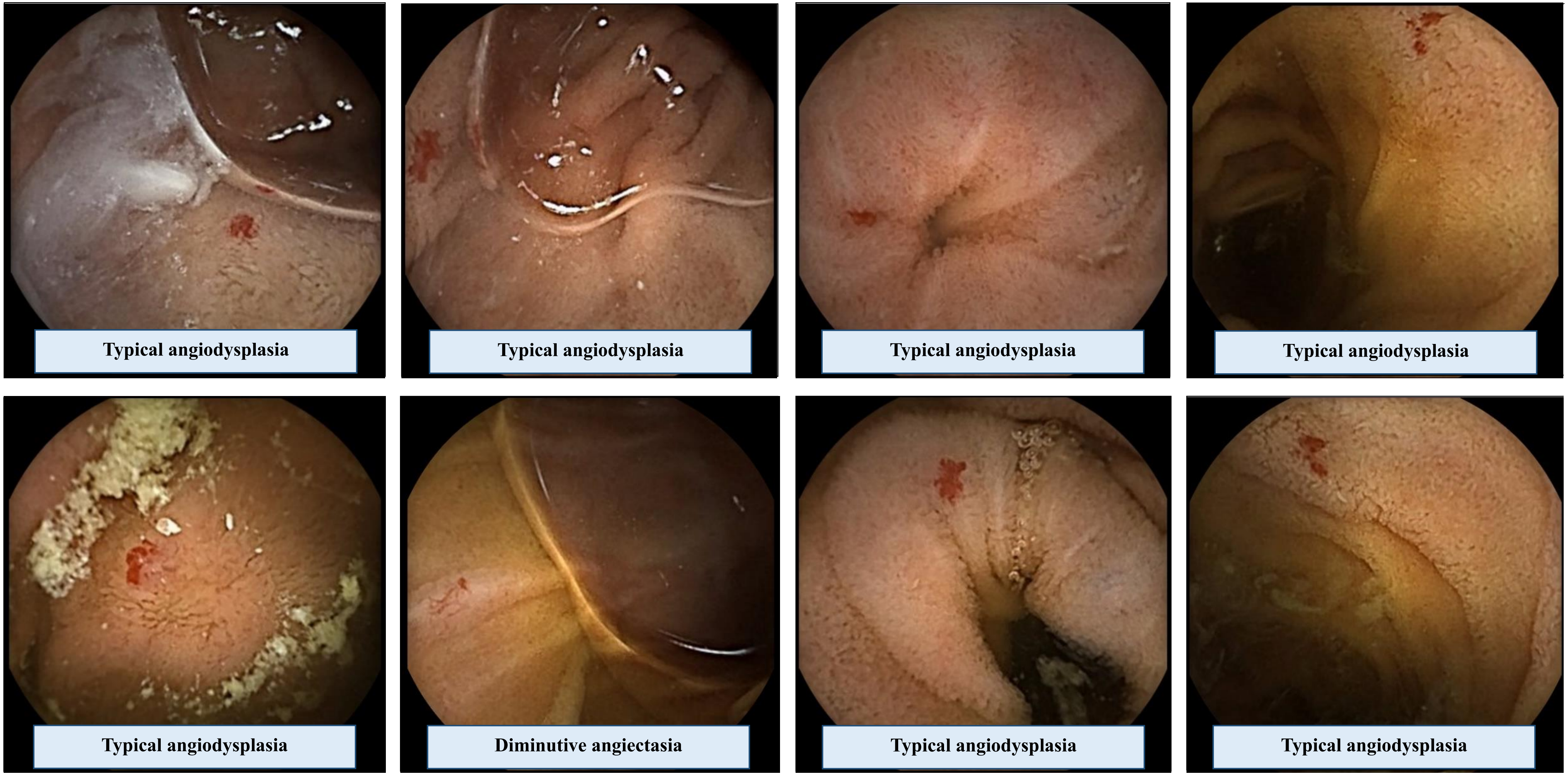

© 2024 Goltstein LMJ et al. *JAMA Network Open.*

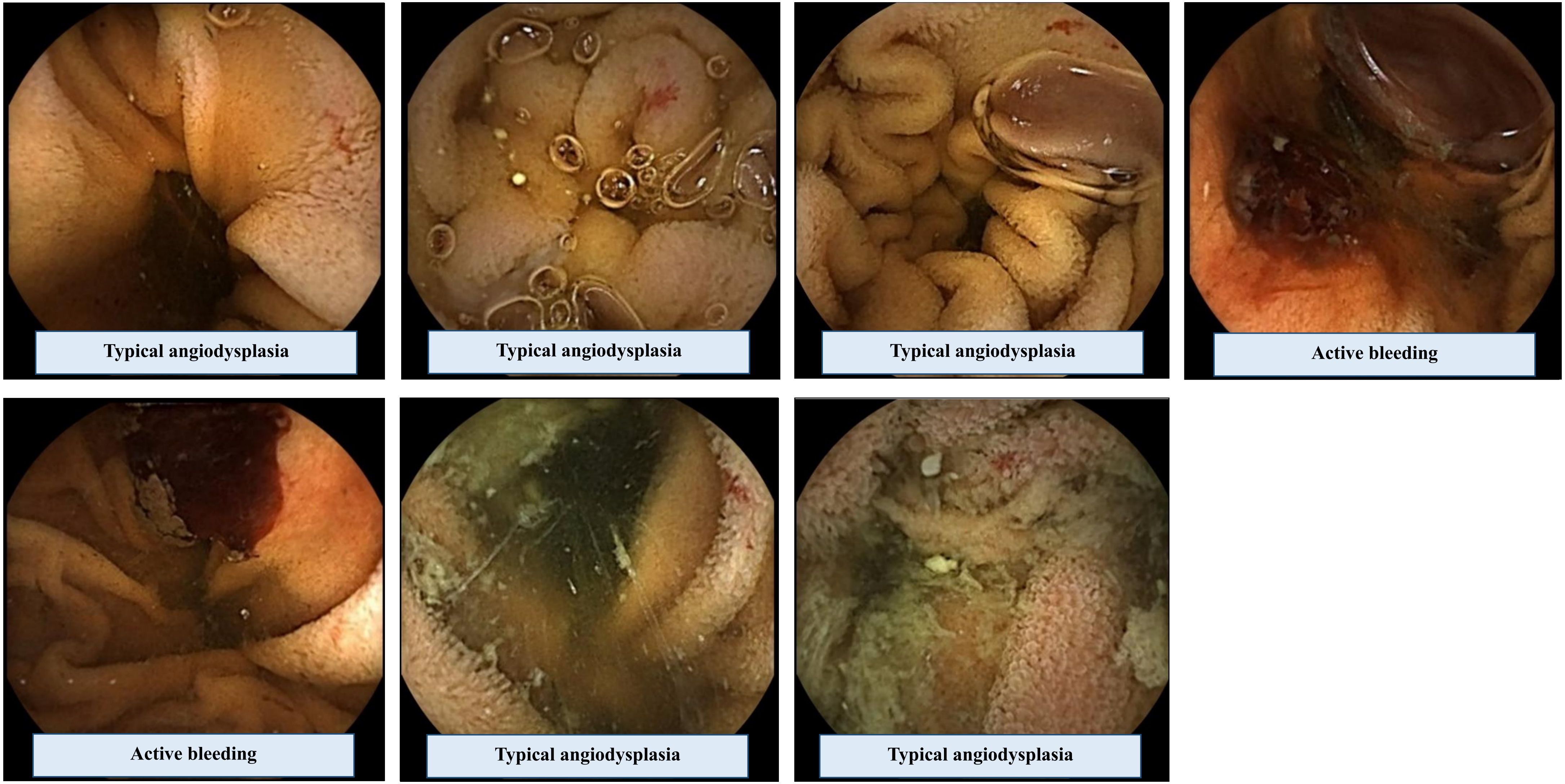

No vascular lesions after TAVI

© 2024 Goltstein LMJ et al. *JAMA Network Open.*

Patient 8 - Before TAVI

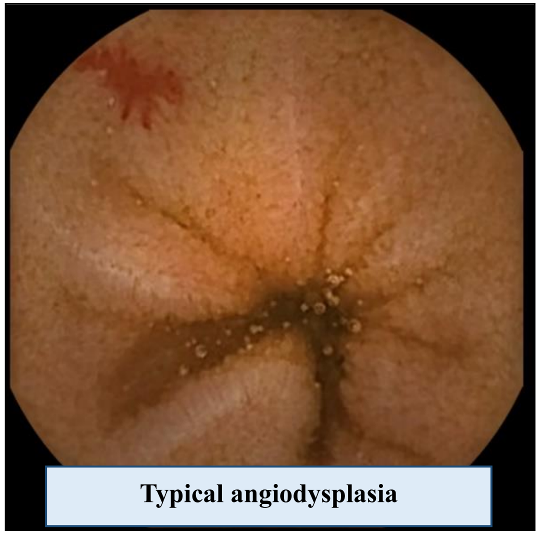

Typical angiodysplasia

Patient 8 - After TAVI

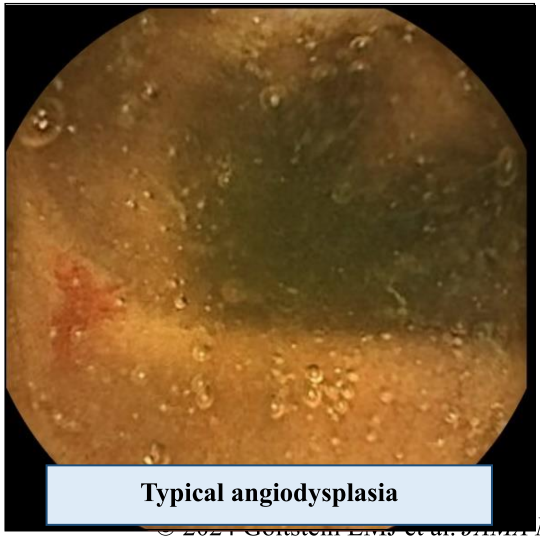

Typical angiodysplasia

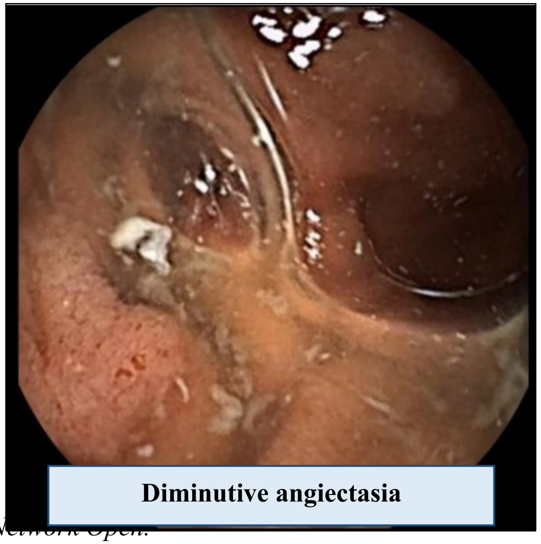

Diminutive angiectasia

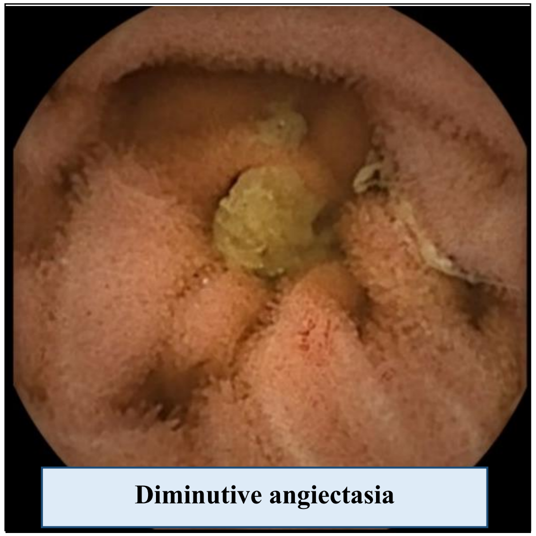

Diminutive angiectasia

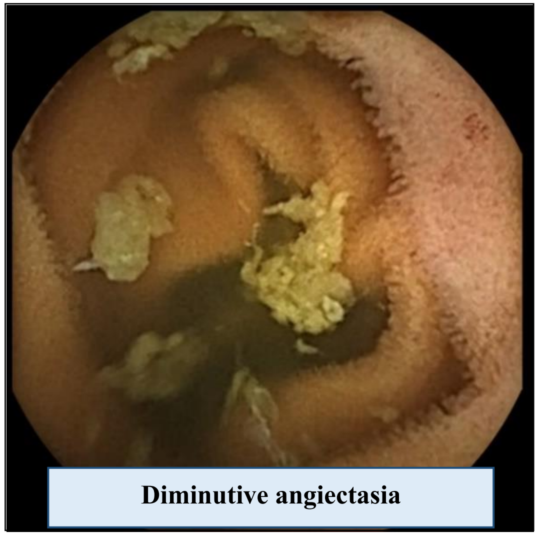

Diminutive angiectasia

© 2024 Goltstein LMJ et al. JAMA Network Open.

Patient 9 - Before TAVI

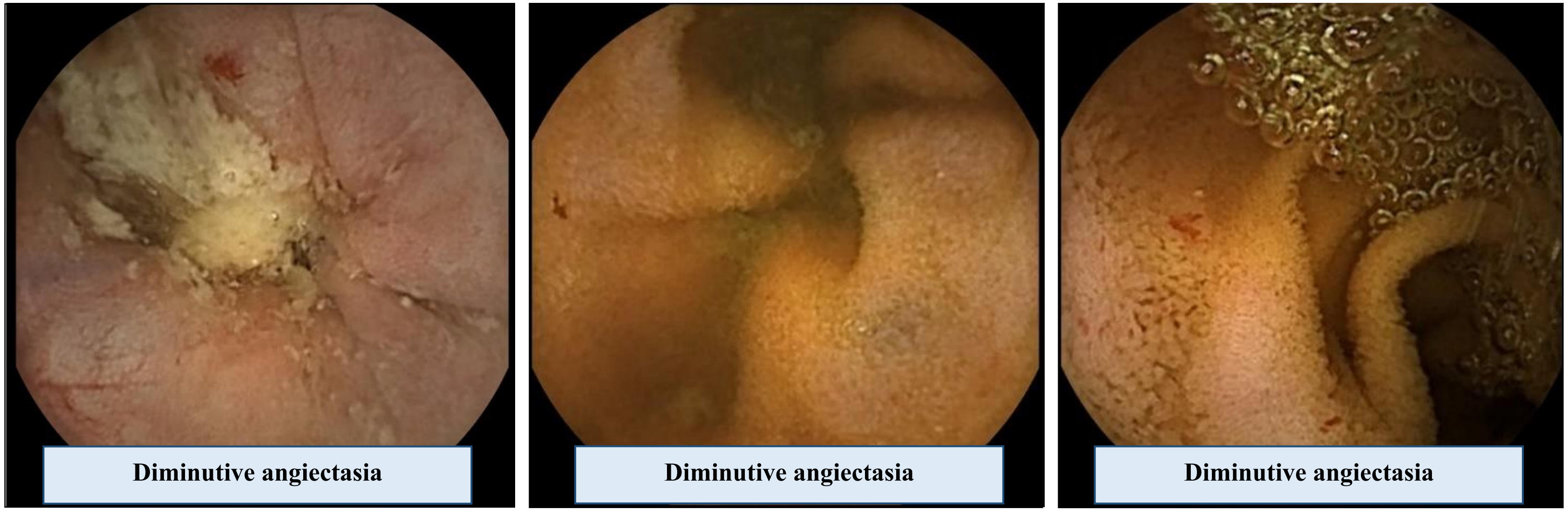

No vascular lesions after TAVI

© 2024 Goltstein LMJ et al. *JAMA Network Open.*

Patient 13 - Before TAVI

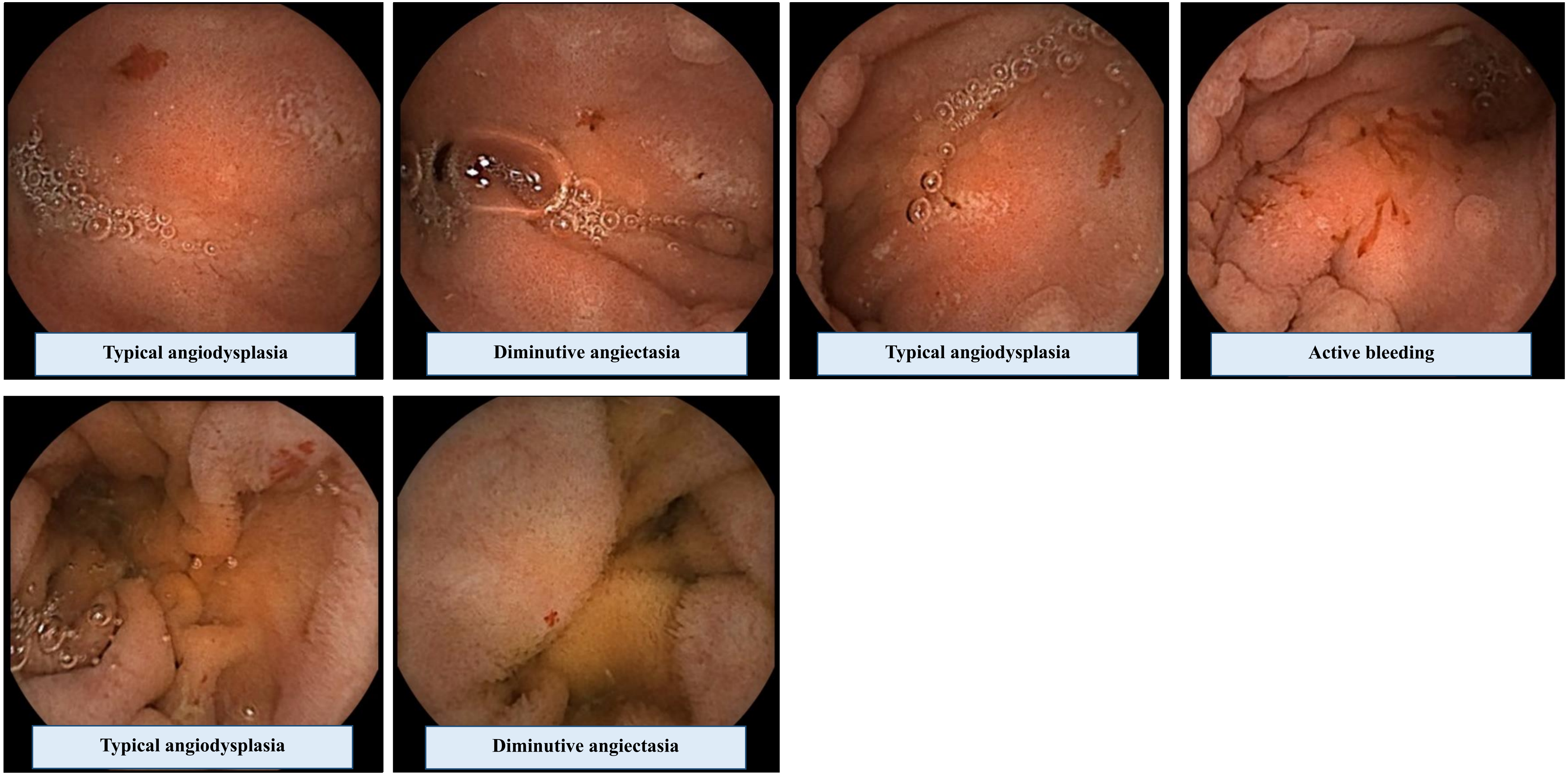

© 2024 Goltstein LMJ et al. *JAMA Network Open.*

Patient 13 - After TAVI

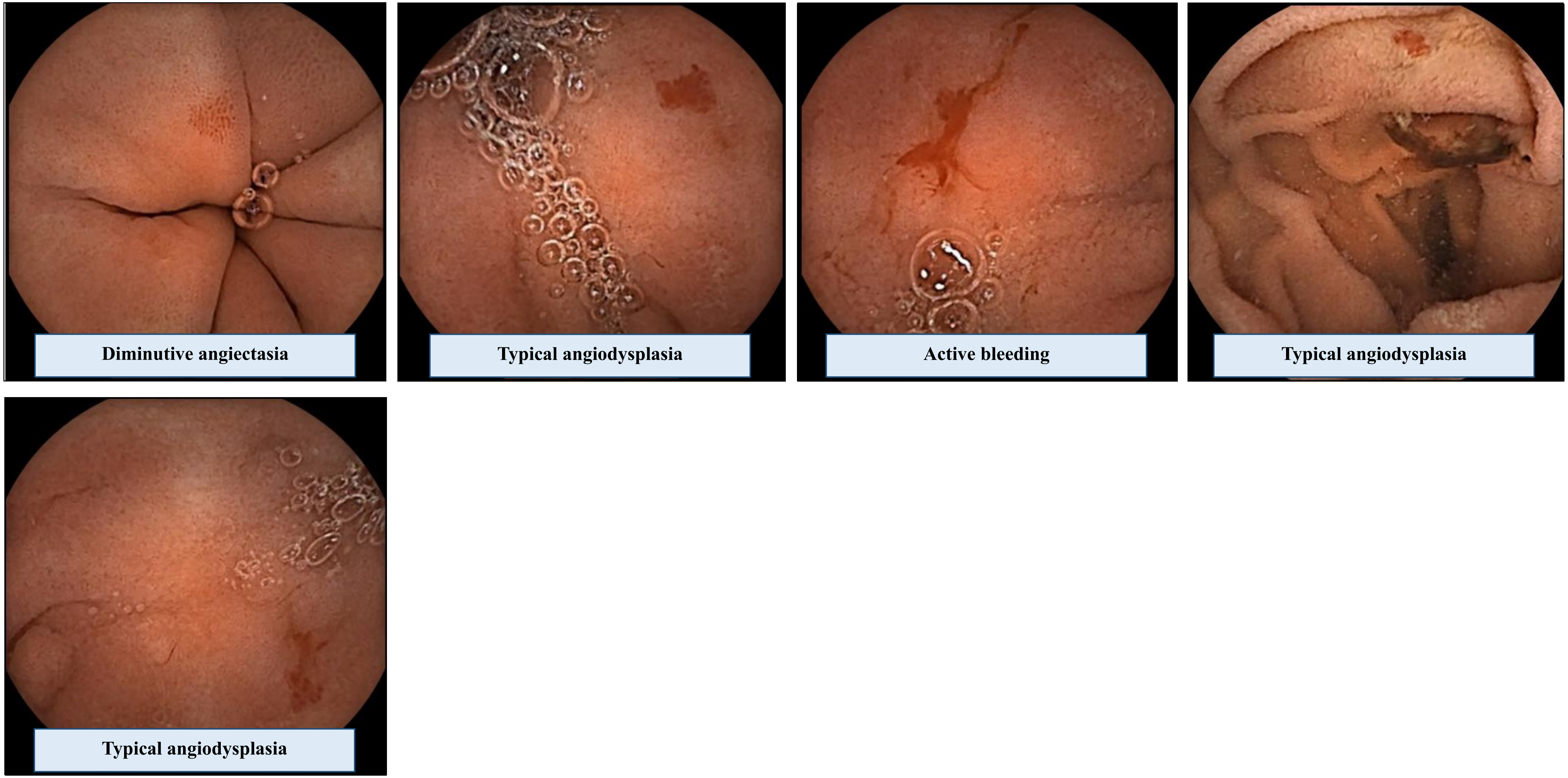

© 2024 Goltstein LMJ et al. *JAMA Network Open.*

Patient 14 - Before TAVI

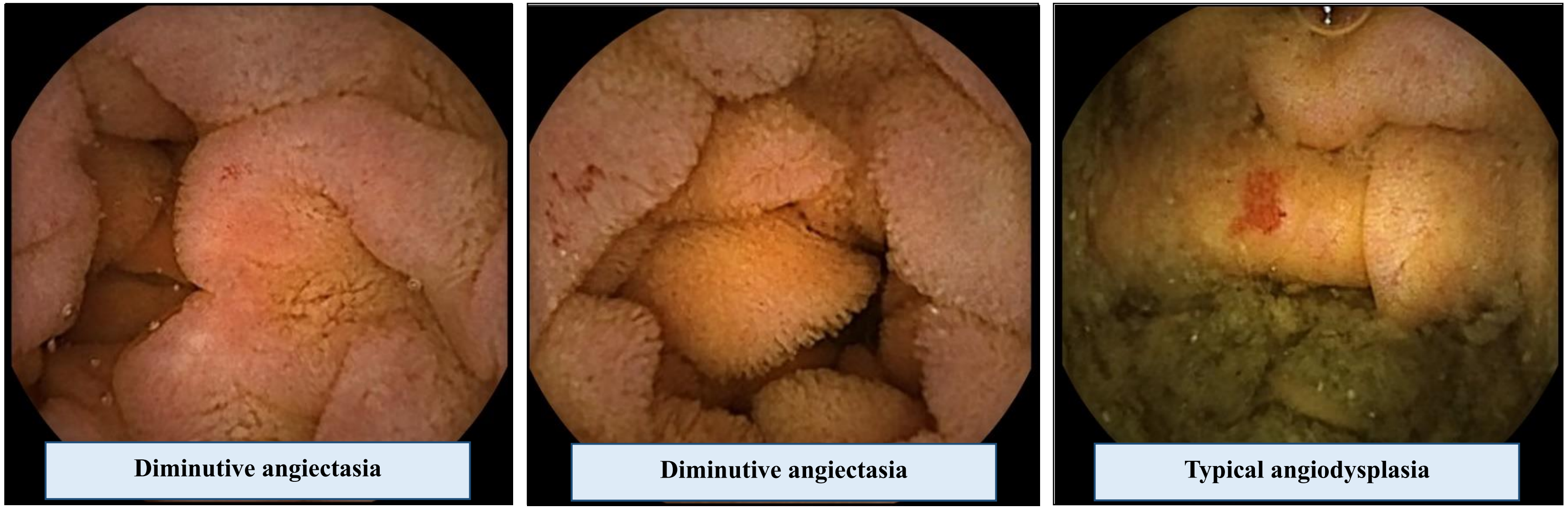

No vascular lesions after TAVI

© 2024 Goltstein LMJ et al. *JAMA Network Open.*

Patient 15 - Before TAVI

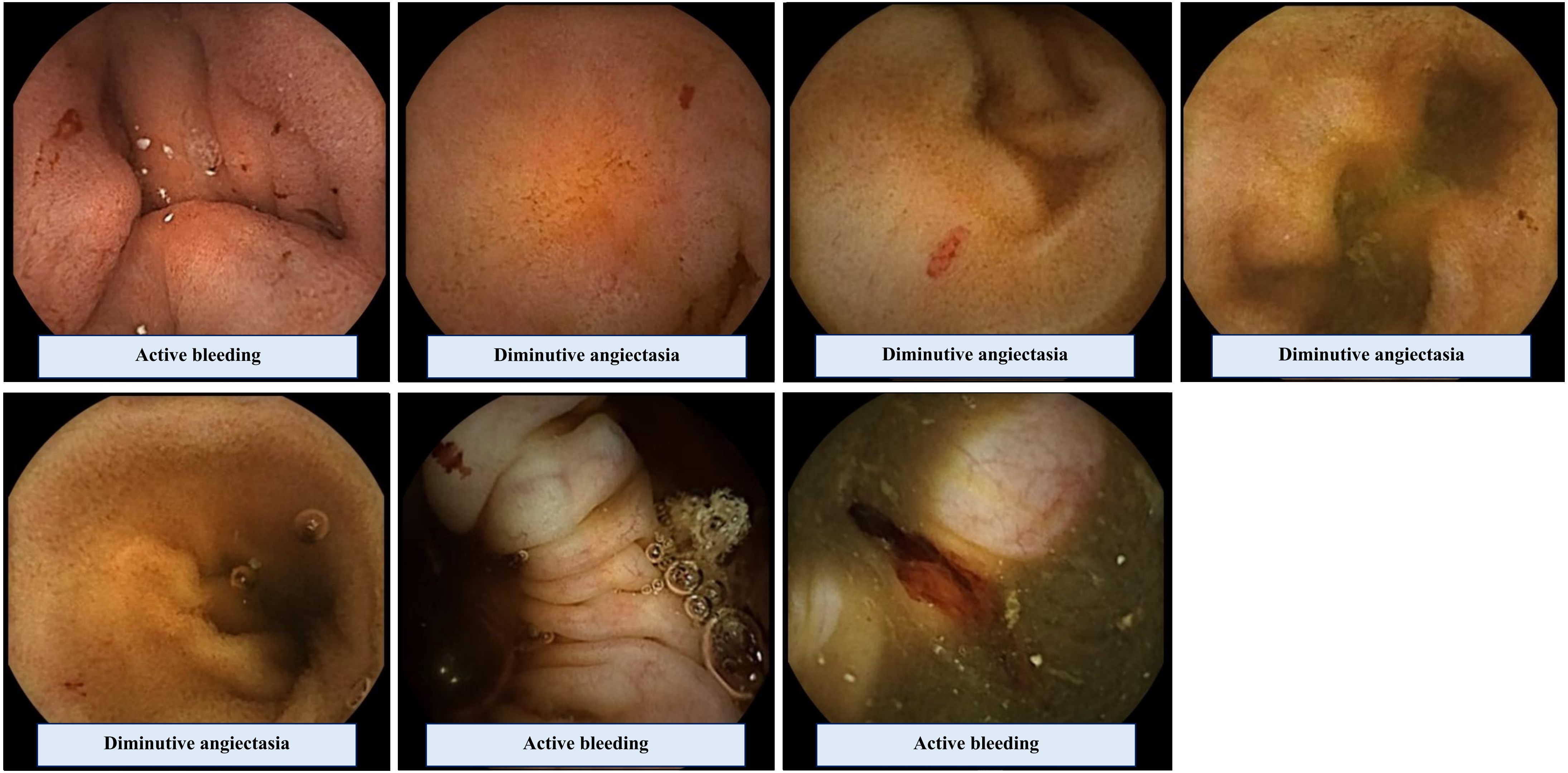

© 2024 Goltstein LMJ et al. *JAMA Network Open.*

Patient 15 - After TAVI

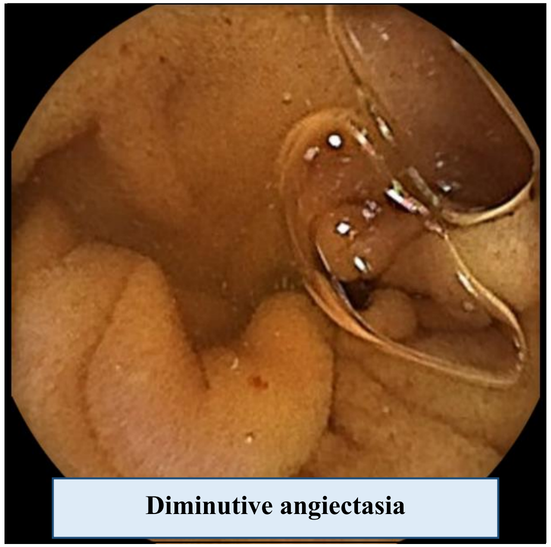

© 2024 Goltstein LMJ et al. *JAMA Network Open.*

Patient 21 - Before TAVI

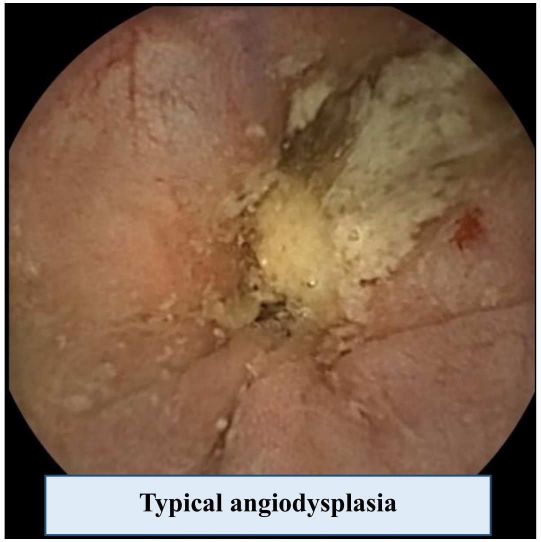
Typical angiodysplasia

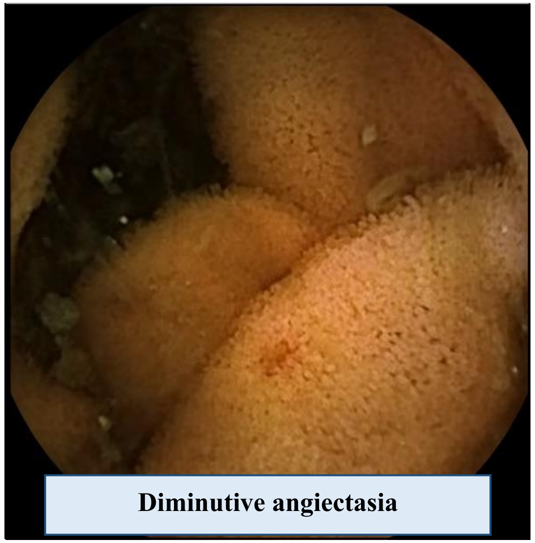
Diminutive angiectasia

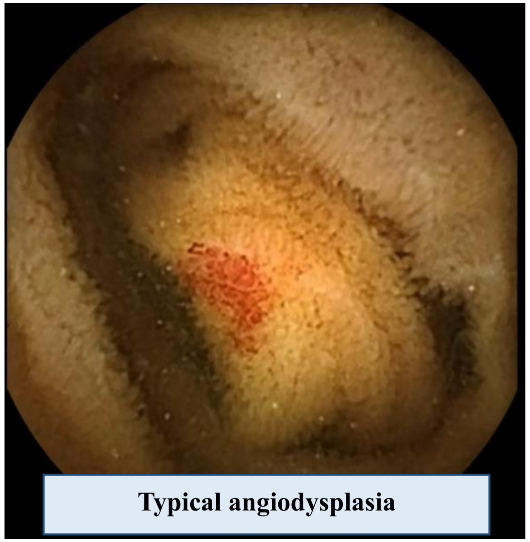
Typical angiodysplasia

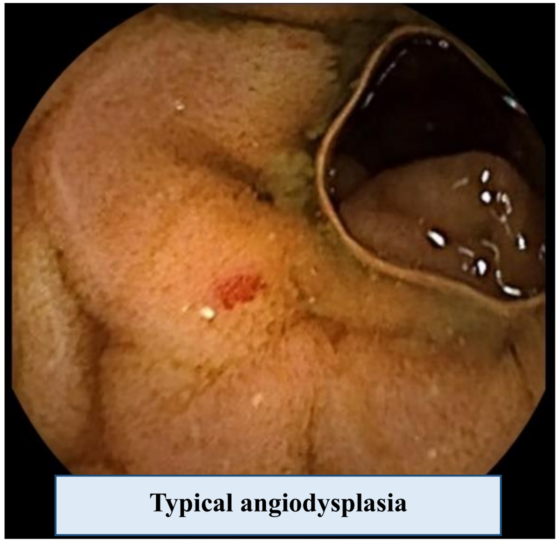
Typical angiodysplasia

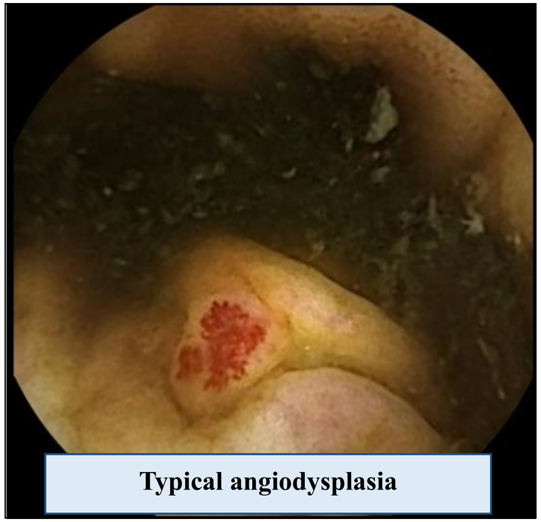
Typical angiodysplasia

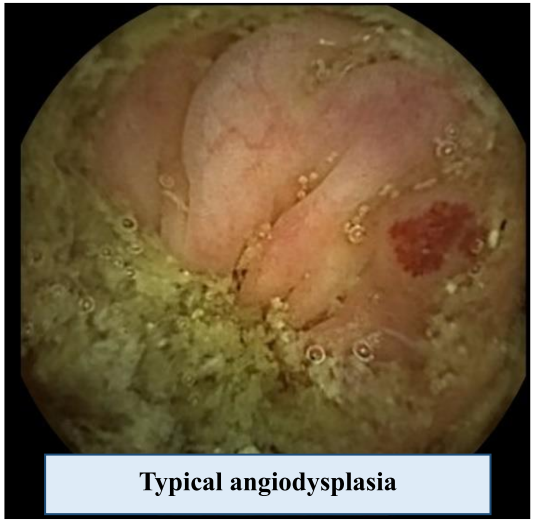
Typical angiodysplasia

© 2024 Goltstein LMJ et al. *JAMA Network Open.*

Patient 21 - After TAVI

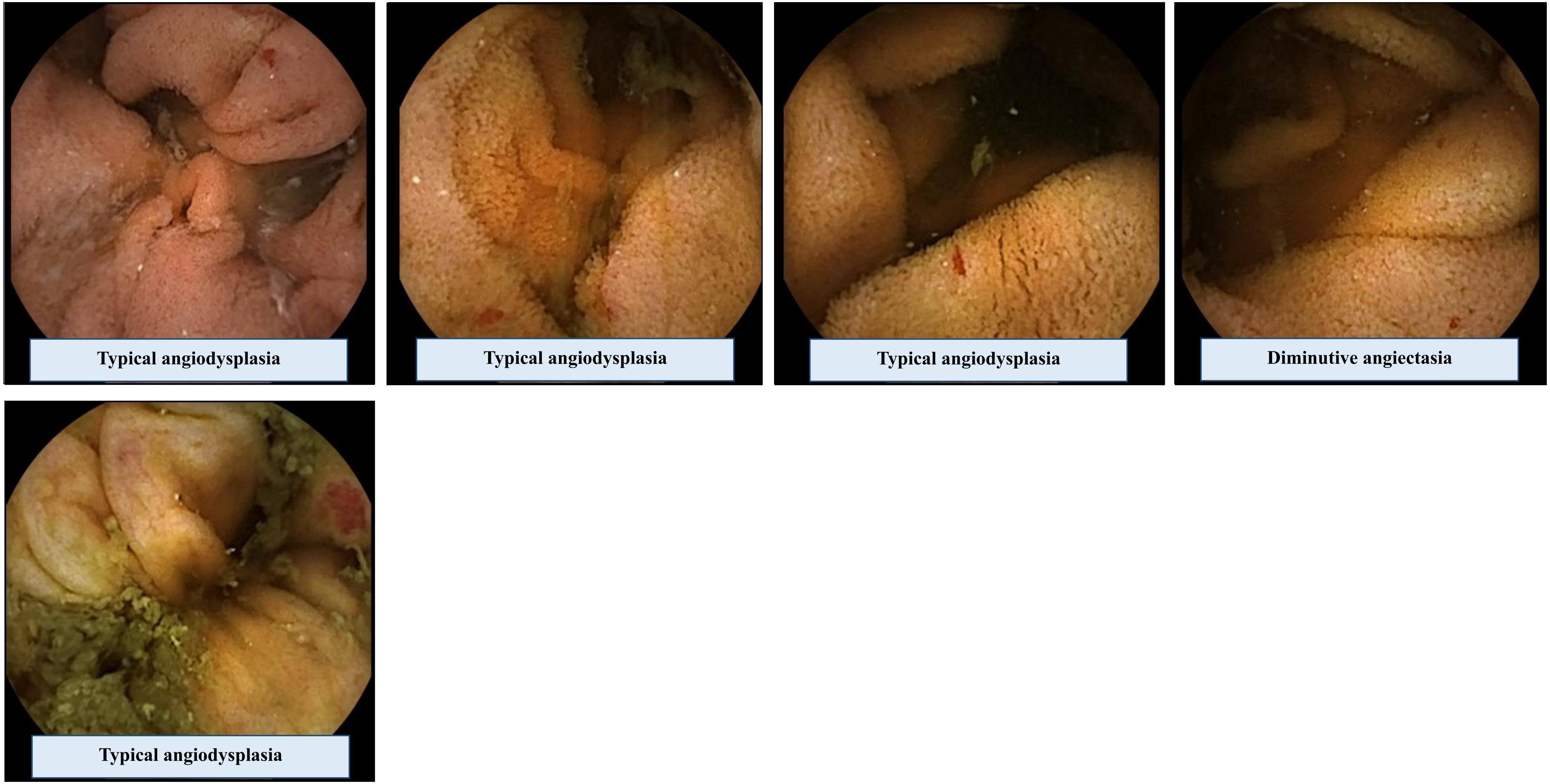

© 2024 Goltstein LMJ et al. *JAMA Network Open.*

Patient 24 - Before TAVI

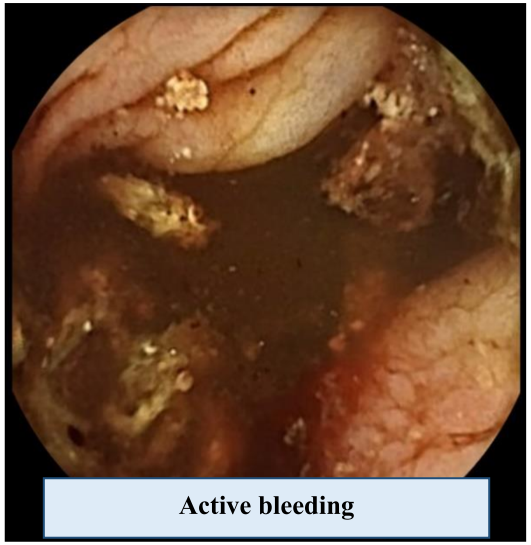

Patient 24 - After TAVI

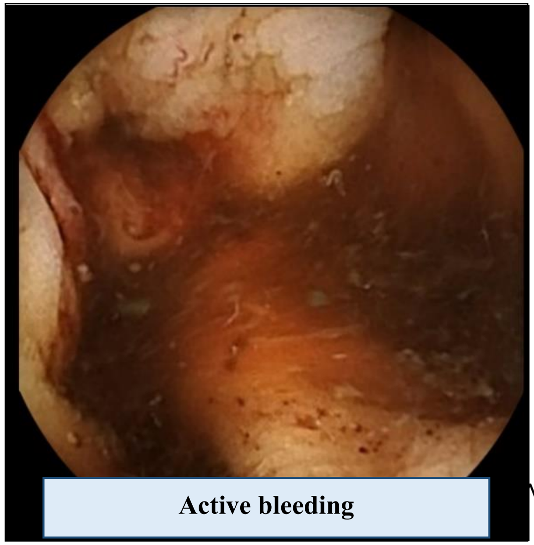

Network Open.

Patient 5 **-** ***No second capsule endoscopy*** **Patient 6** **-** ***No second capsule endoscopy***

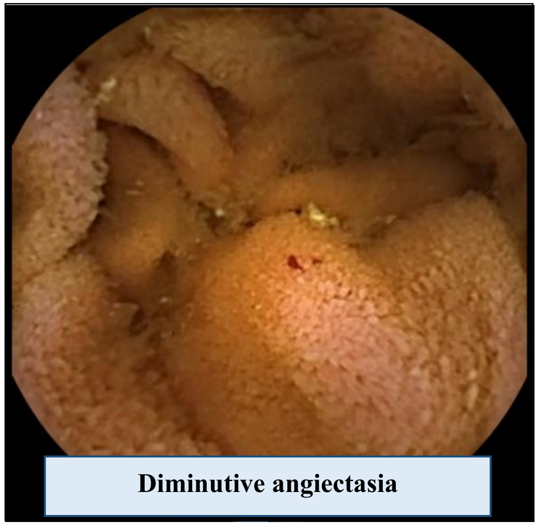

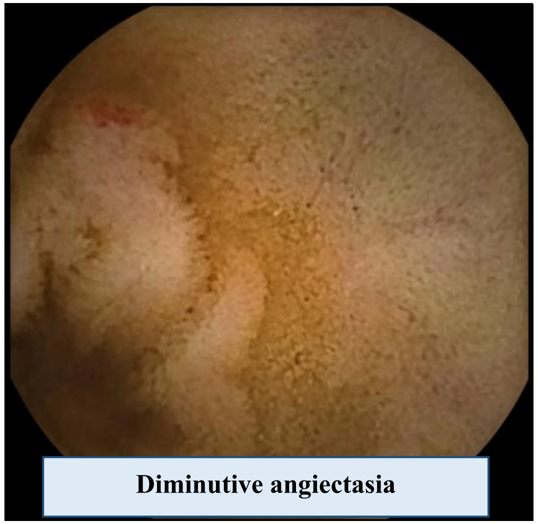

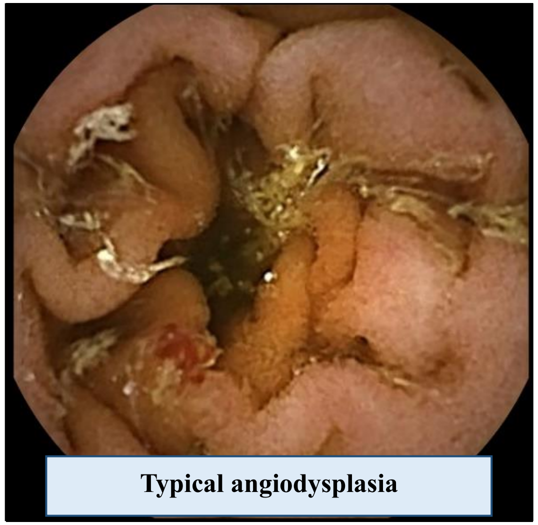

Patient 18 **-** ***No second capsule endoscopy***

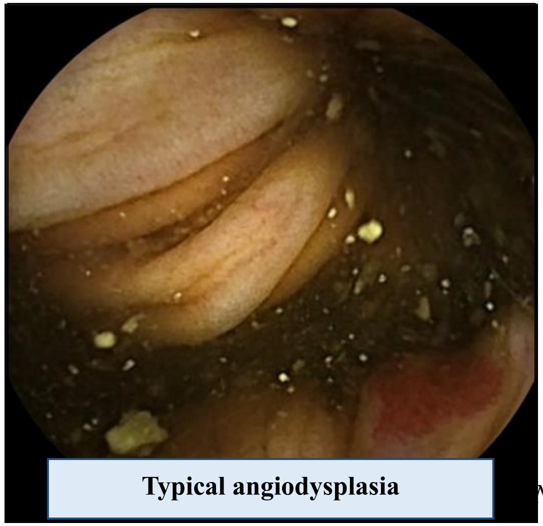

Patient 19 - *No second capsule endoscopy*

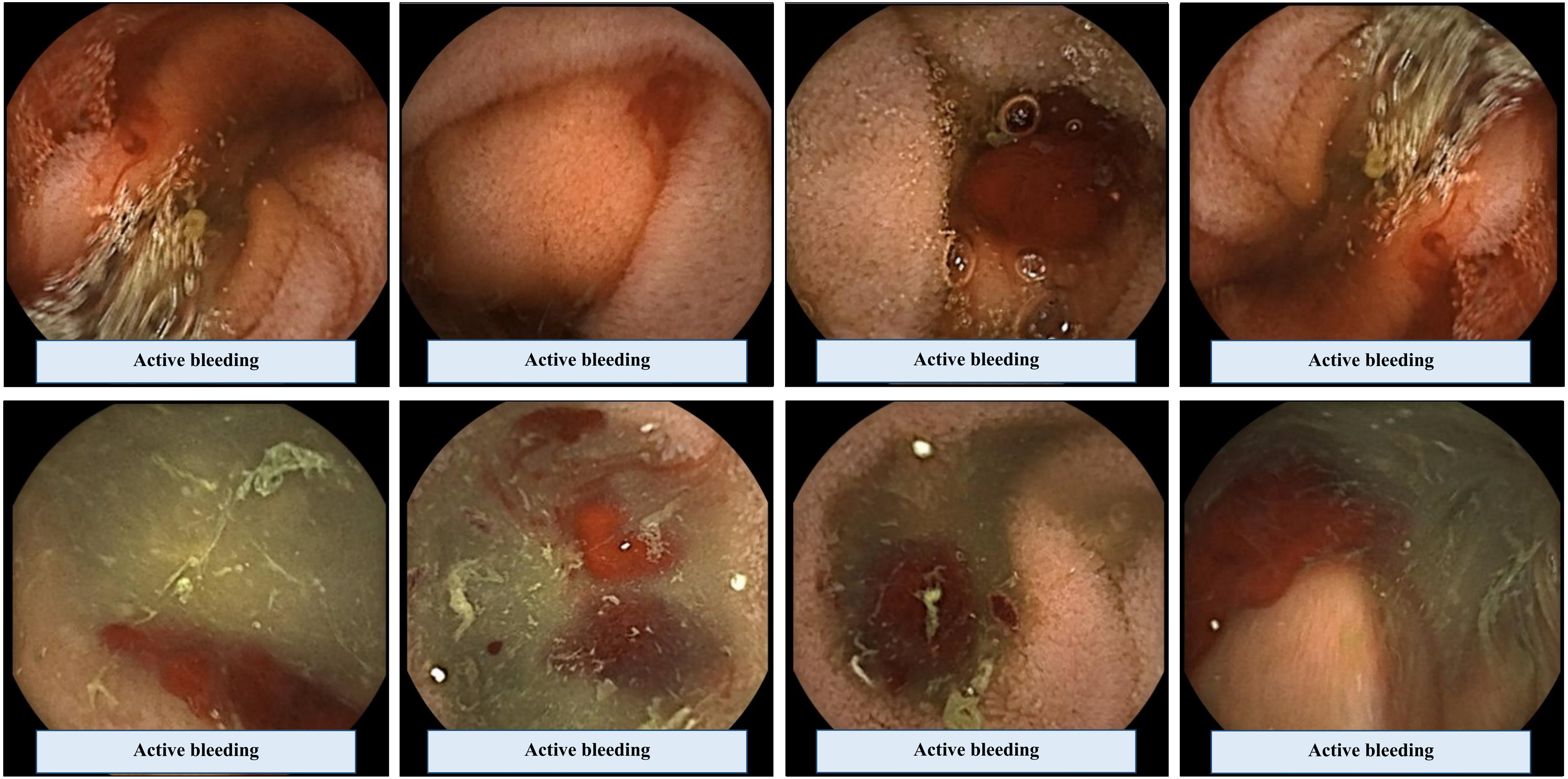

© 2024 Goltstein LMJ et al. *JAMA Network Open.*

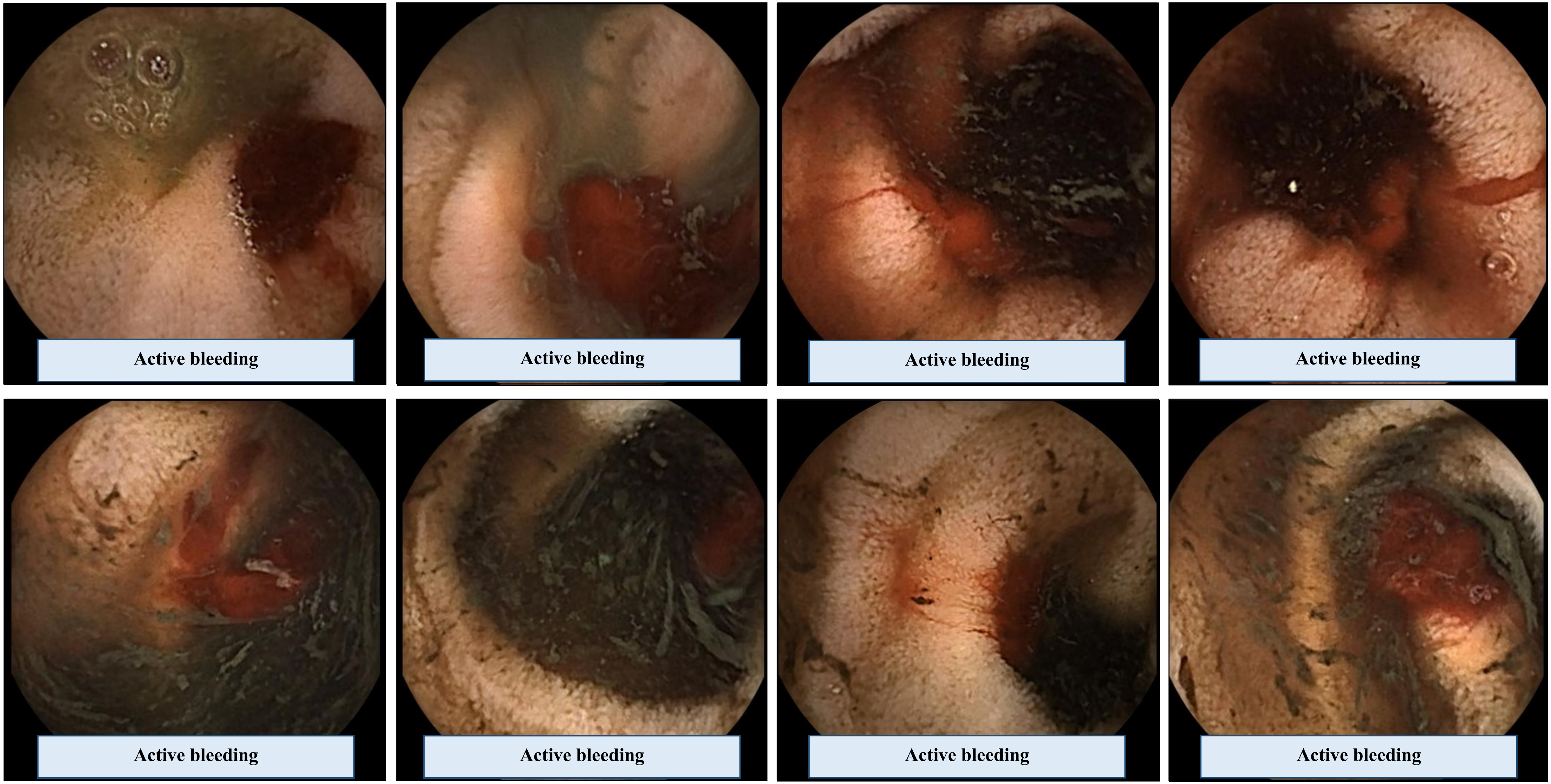

© 2024 Goltstein LMJ et al. *JAMA Network Open.*

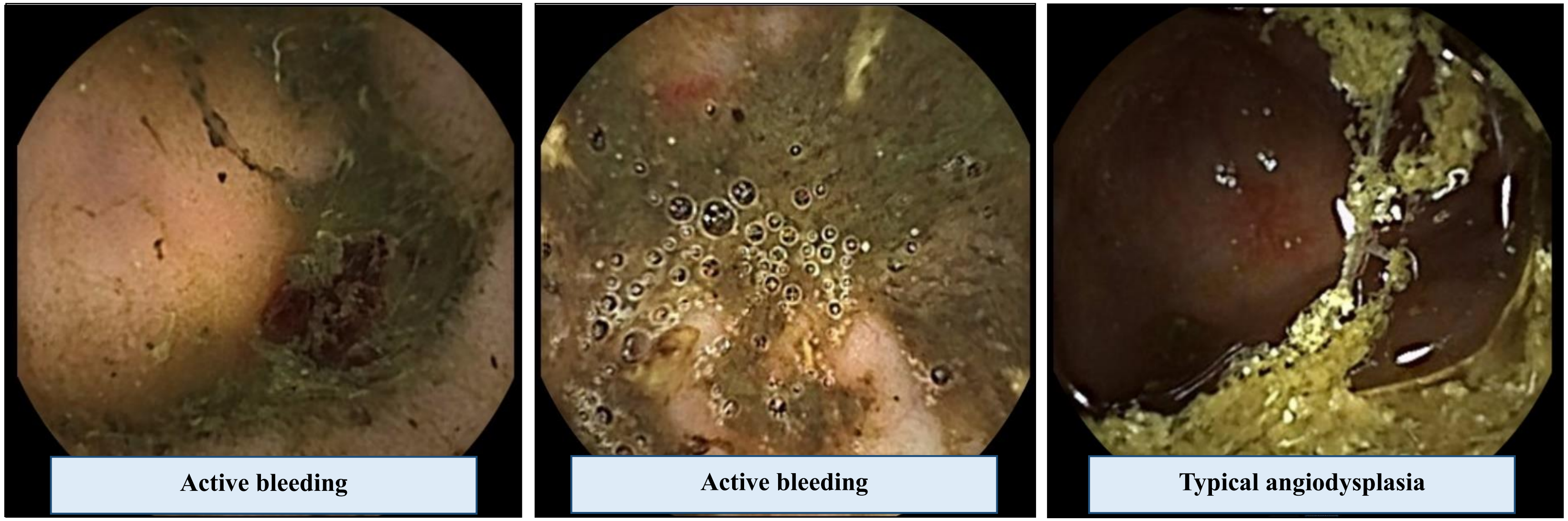

© 2024 Goltstein LMJ et al. *JAMA Network Open.*

Patient 2 - *Not eligible for TAVI*

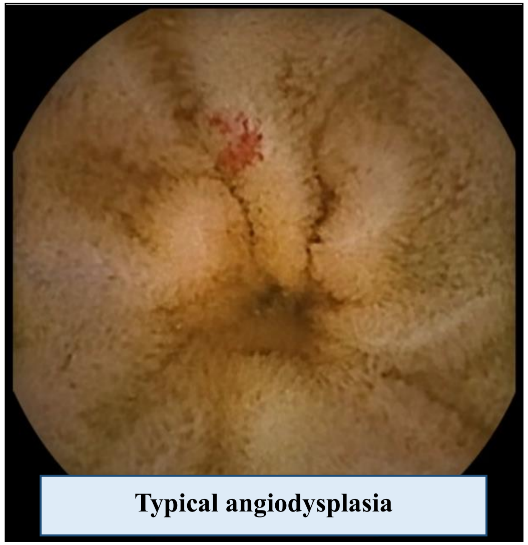

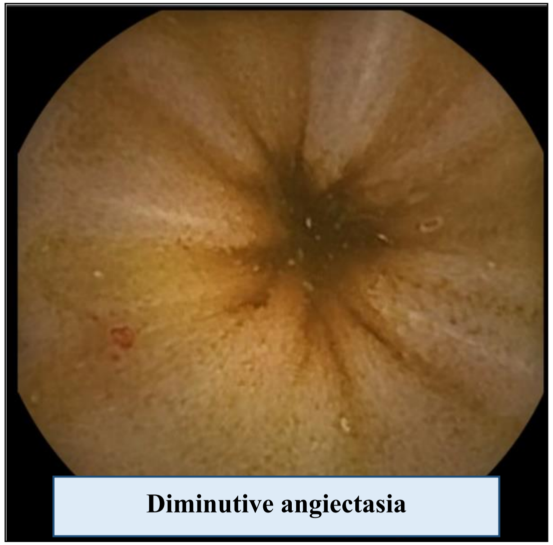

Patient 12 - *Not eligible for TAVI*

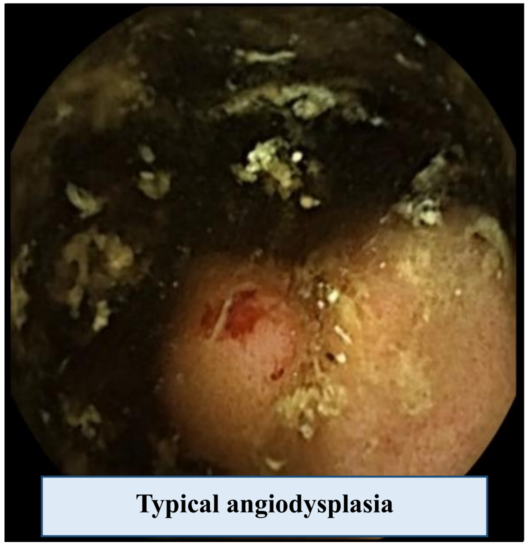

Patient 20 - *Not eligible for TAVI*

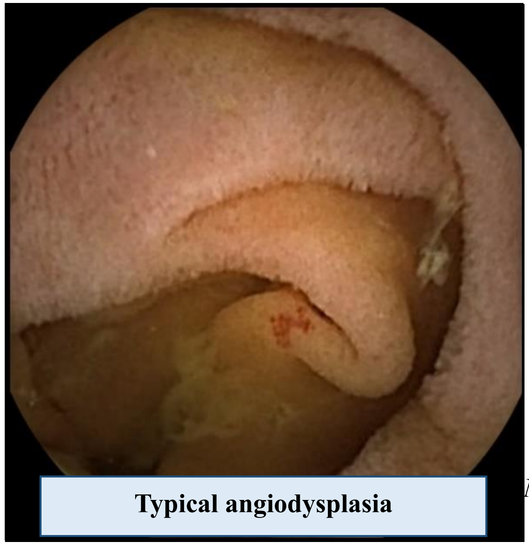

Network Open.

eFigure 3. Transfusion requirements of patients with Heyde syndrome

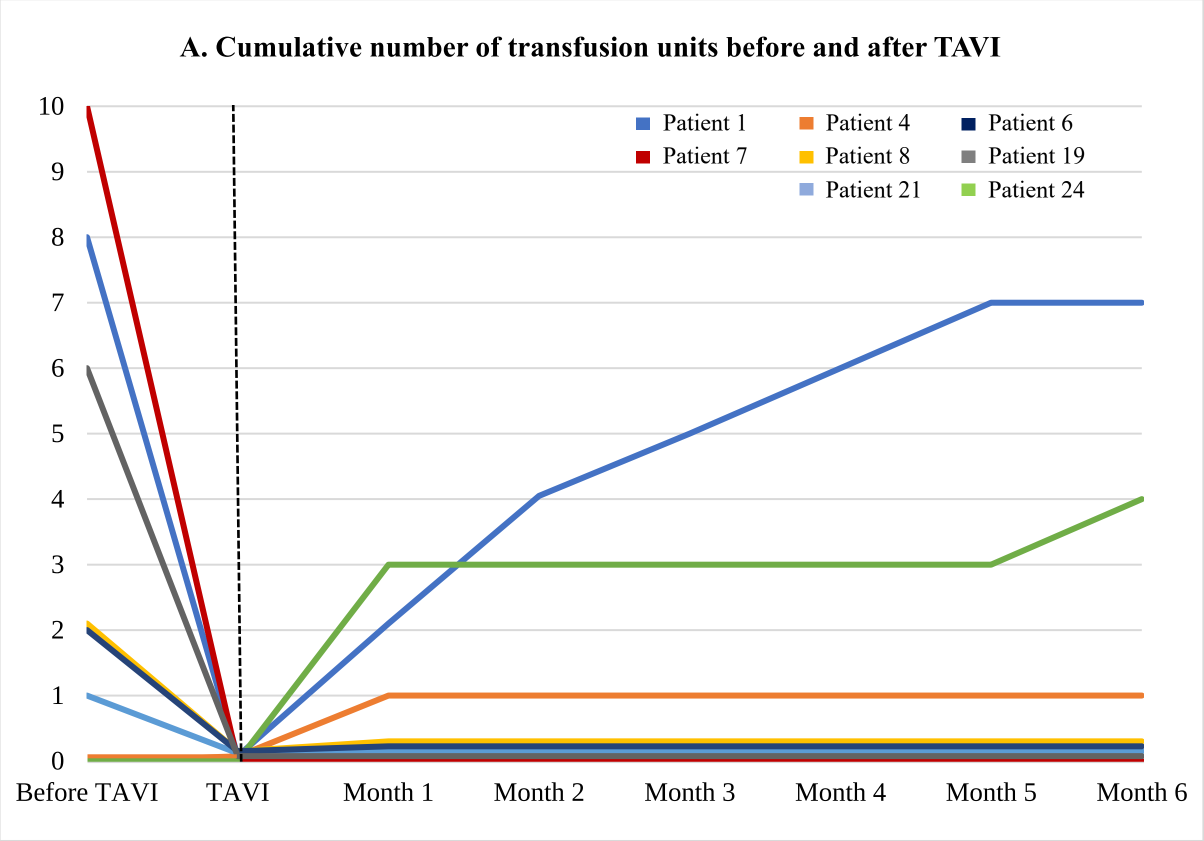

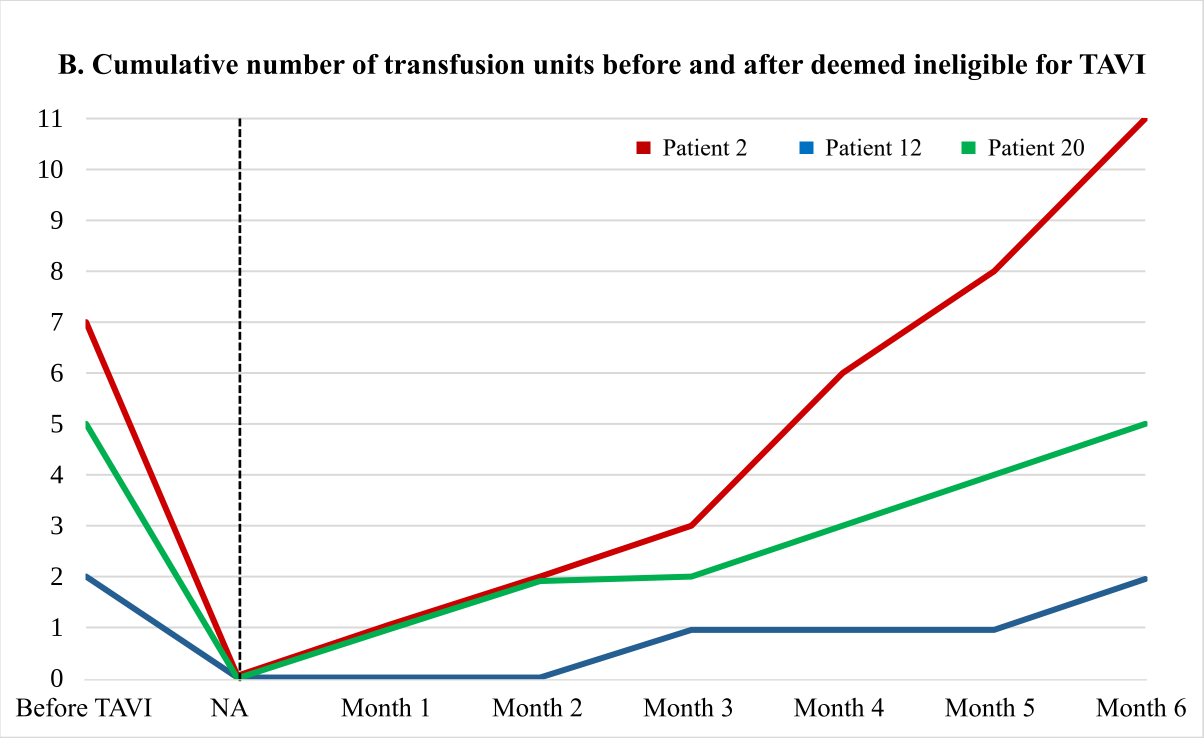

Abbreviations: NA, not applicable; TAVI, transcatheter aortic valve implantation.
The line graphs illustrate the cumulative number of transfusion units (consisting of blood transfusions [per packed cells] and/or intravenous iron infusions [per 500mg] patients with Heyde syndrome required in the six months before TAVI and after study inclusion. Figure A illustrates the results of the 15 patients who underwent TAVI. Seven patients were not included, as they did not require any transfusions before or after TAVI. Figure B shows the results of the three patients who were deemed ineligible for TAVI. For these patients, the date of the multidisciplinary heart team meeting was used instead of the date of TAVI (NA).

eFigure 4. Densitometry plots of the von Willebrand factor multimer distribution

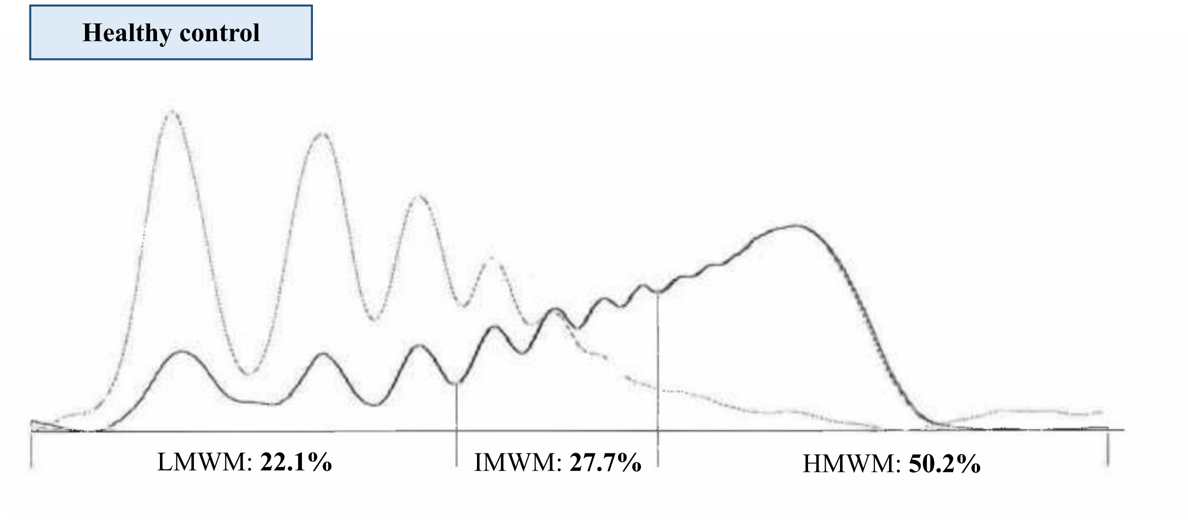

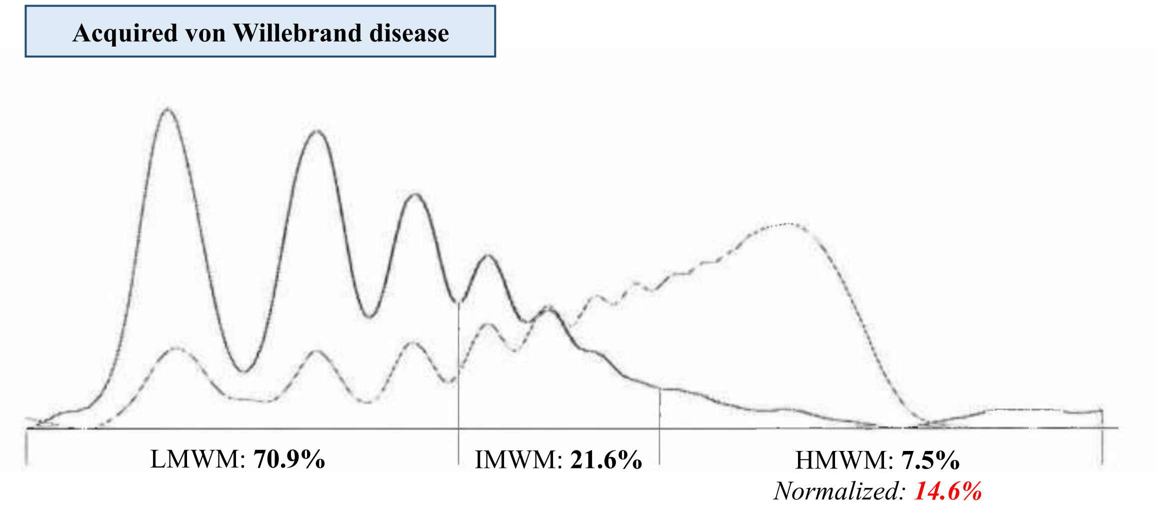

Abbreviations: AVWD, acquired von Willebrand disease; vWF, von Willebrand factor.
vWF-LMWM, proportion of vWF light-molecular-weight multimers.
vWF-IMWM, proportion of vWF intermediate-molecular-weight-multimers.
vWF-HMWM, proportion of vWF high-molecular-weight-multimers.

The figures illustrate the densitometry plots of the vWF distribution of all patients at four time points: the first capsule endoscopy appointment (T1), <72 hours after TAVI (T2), the three-month follow-up appointment (T3), and the second capsule endoscopy appointment approximately six months after TAVI (T4).[6] The vWF-HMWM proportion of patients was normalized to the mean vWF-HMWM proportion of healthy individuals (n=97, mean 51.3%).[6] A patient with a normalized vWF-HMWM proportion <80% is considered to have AVWD.[7,8]

© 2024 Goltstein LMJ et al. *JAMA Network Open.*

Patient 1

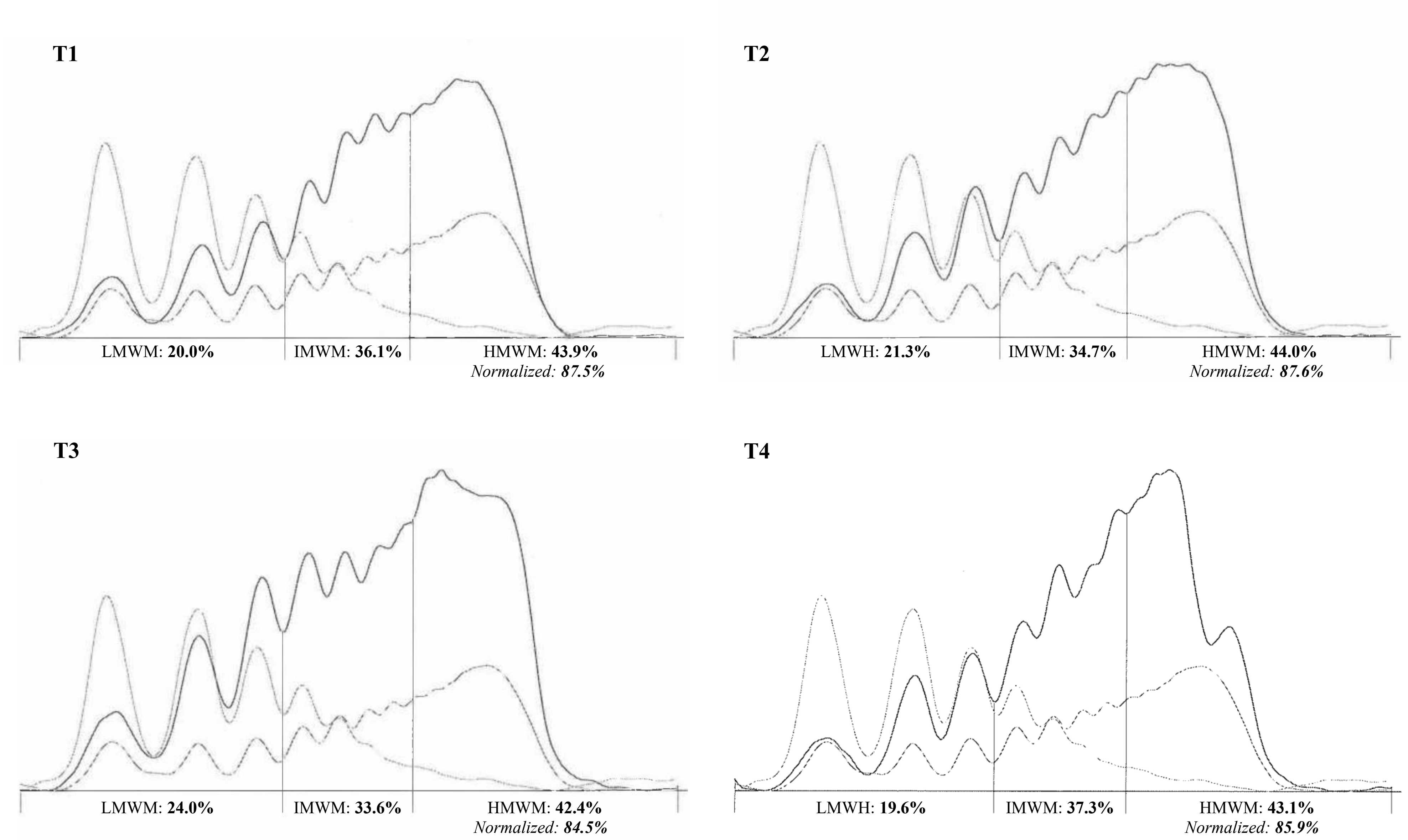

© 2024 Goltstein LMJ et al. *JAMA Network Open.*

Patient 3

T1

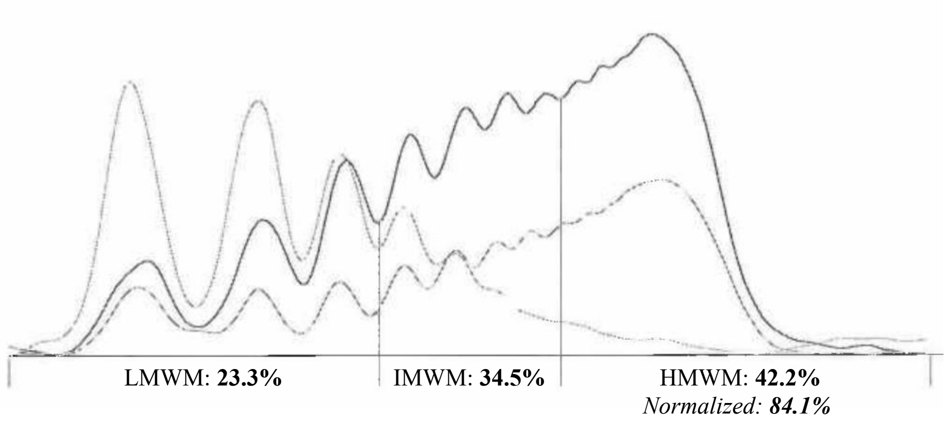

T2

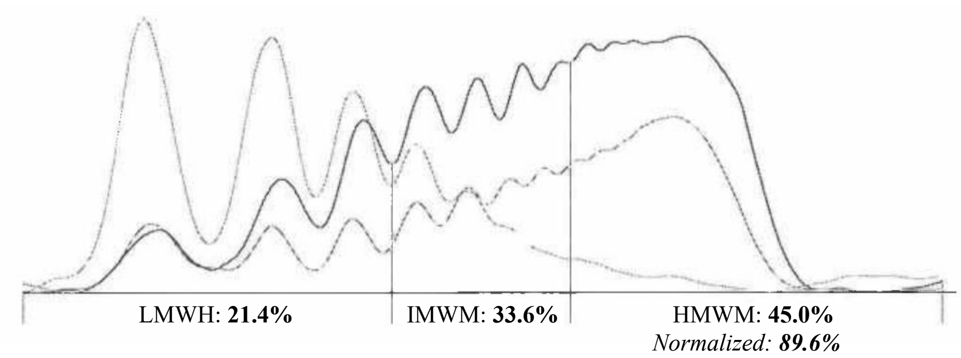

T3

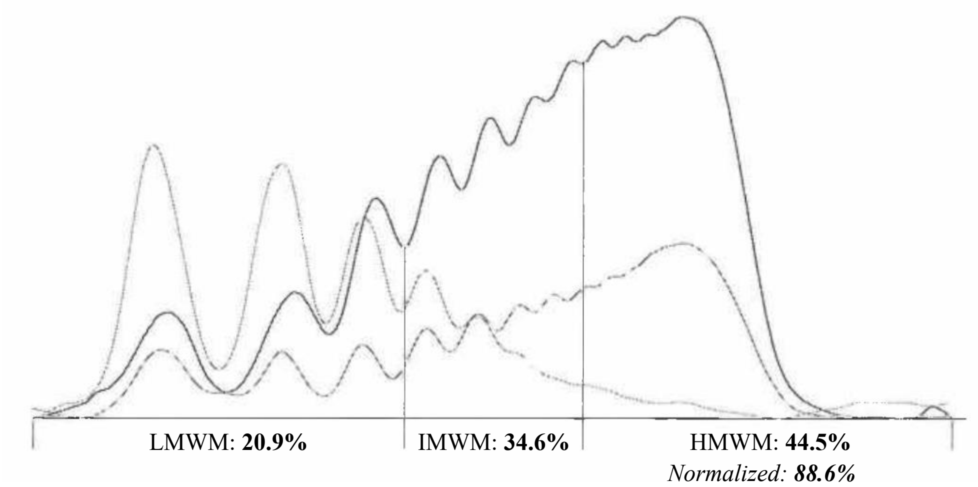

T4

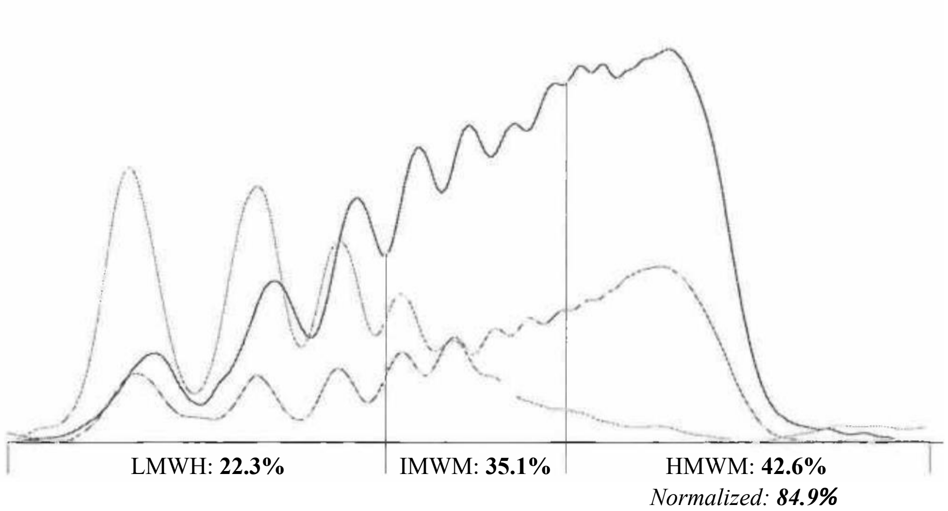

© 2024 Goltstein LMJ et al. *JAMA Network Open.*

Patient 4

T1

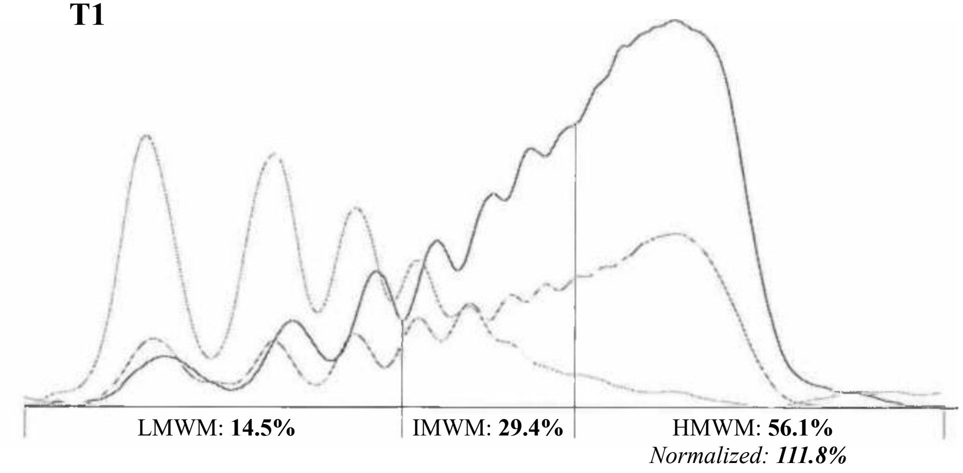

T2

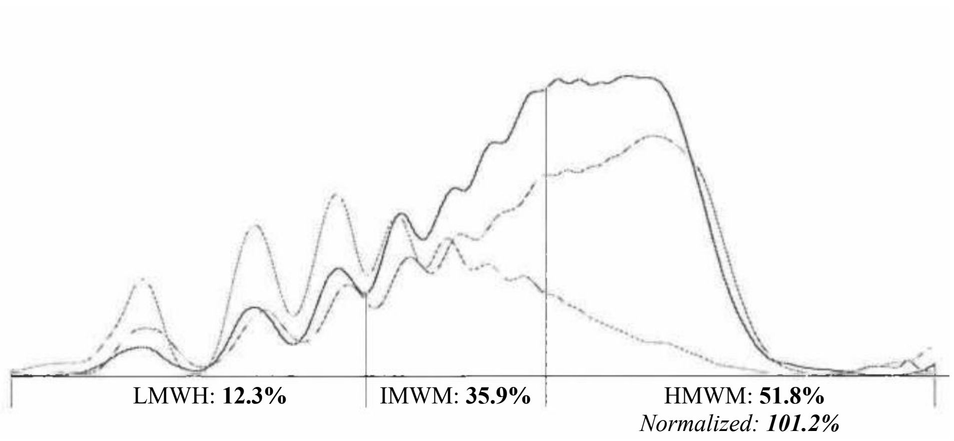

T3

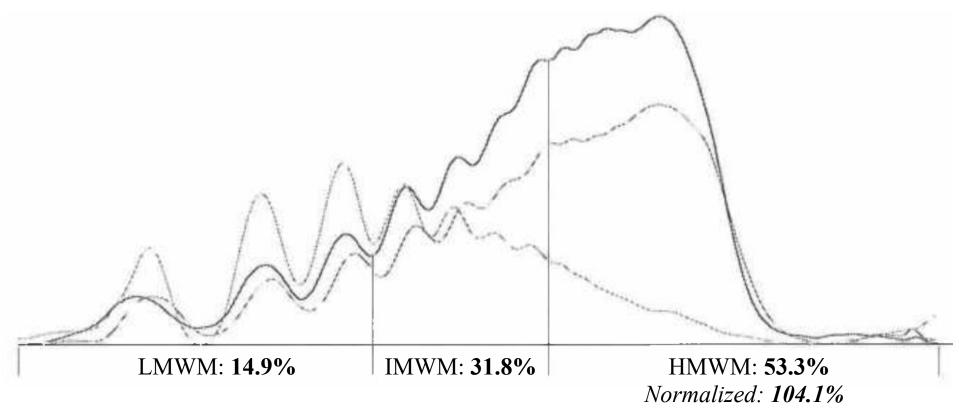

T4

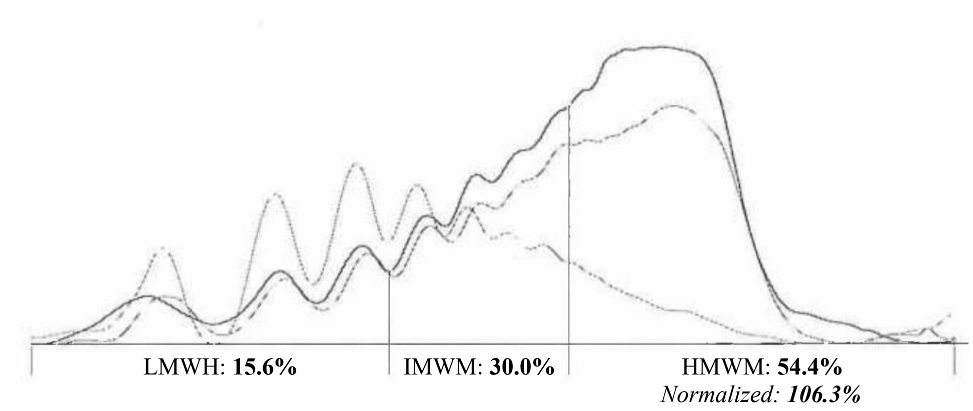

© 2024 Goltstein LMJ et al. *JAMA Network Open.*

Patient 7

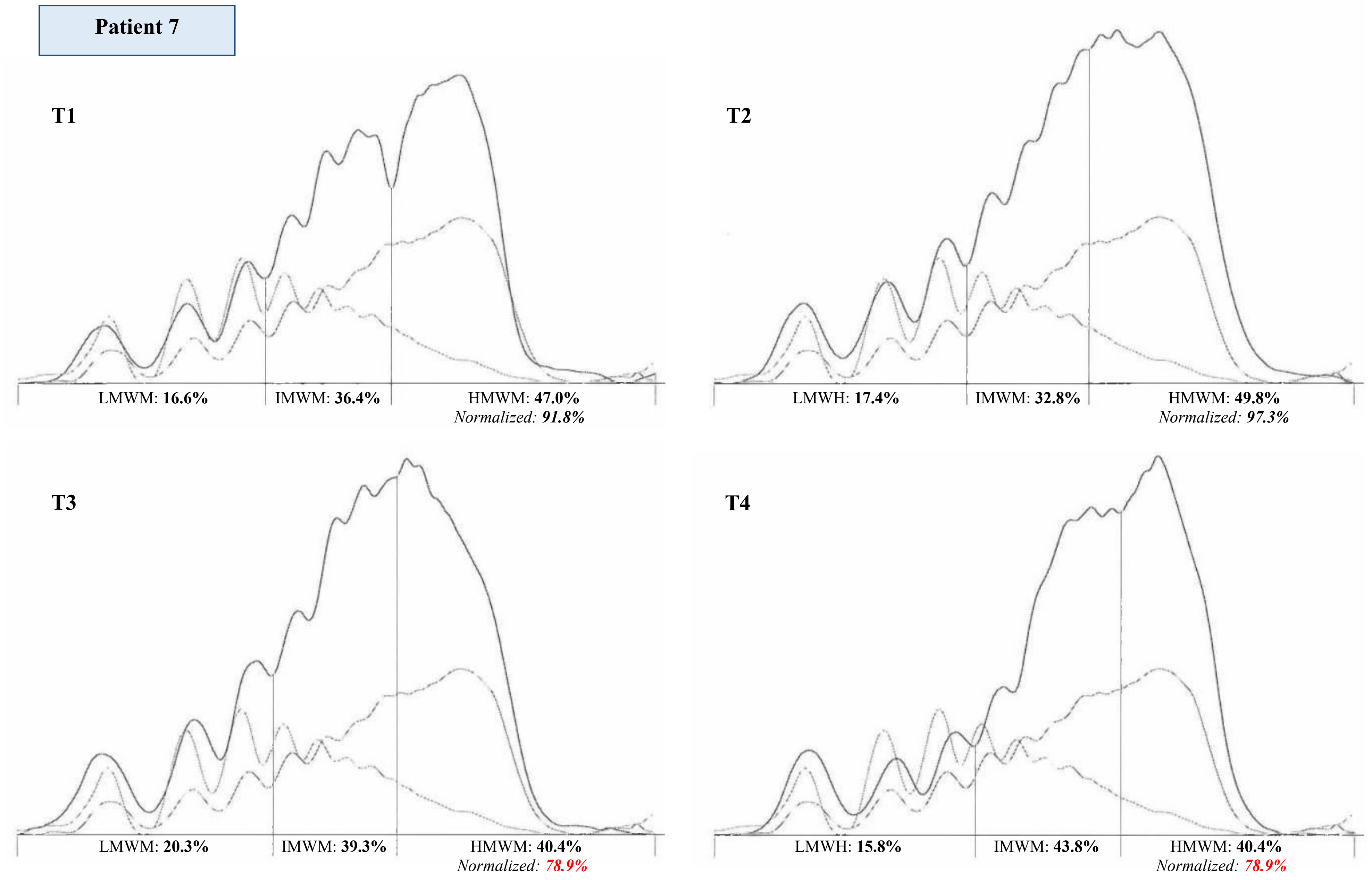

© 2024 Goltstein LMJ et al. *JAMA Network Open.*

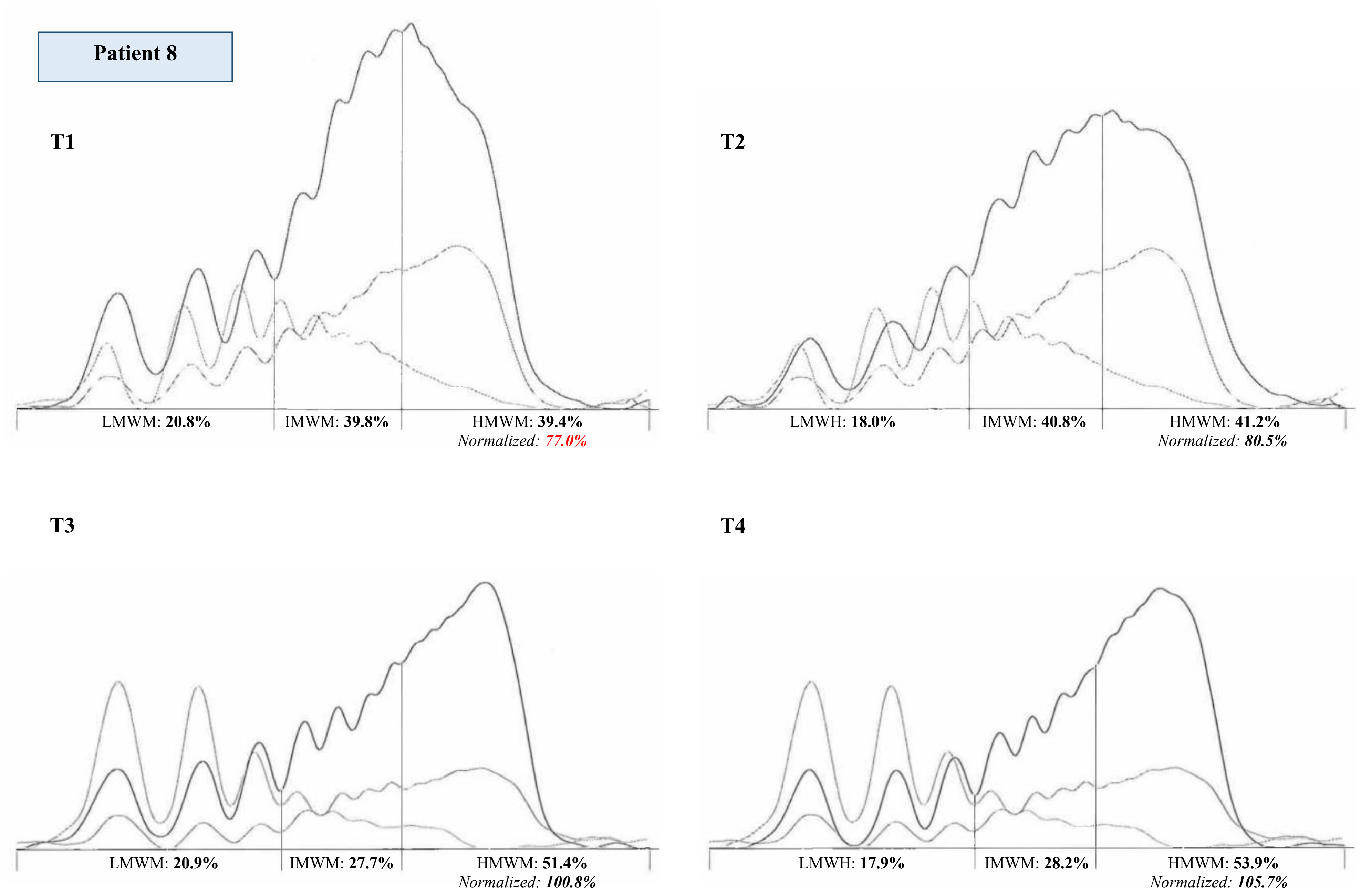

© 2024 Goltstein LMJ et al. *JAMA Network Open.*

Patient 9

T1

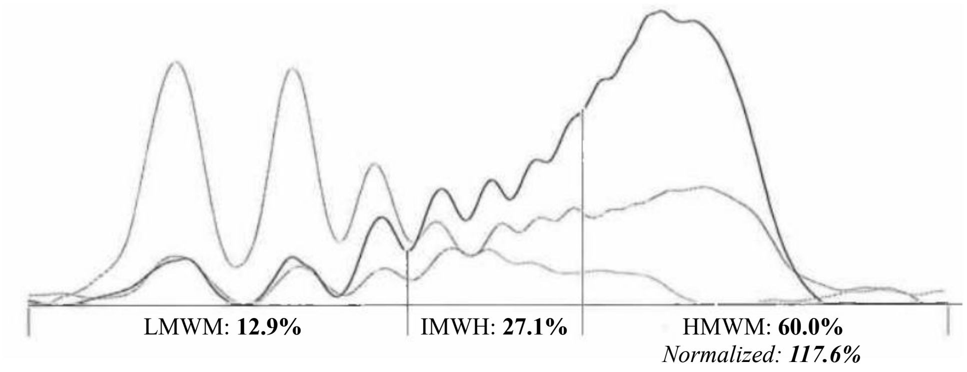

T2

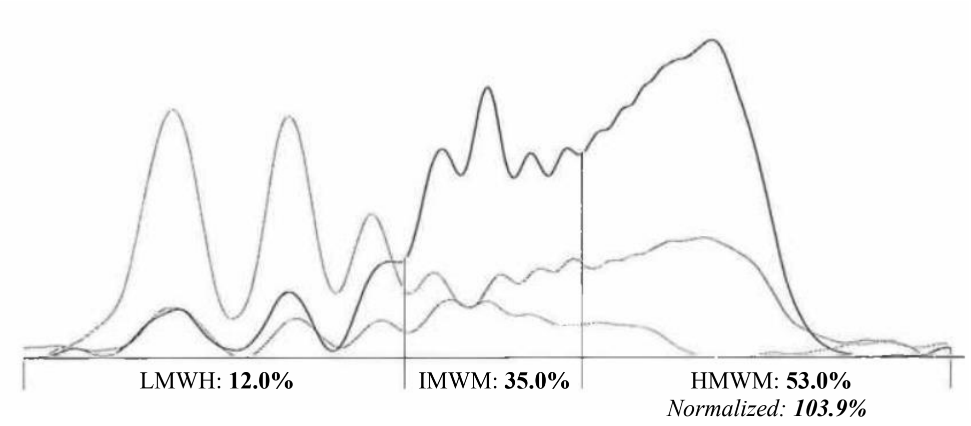

T3

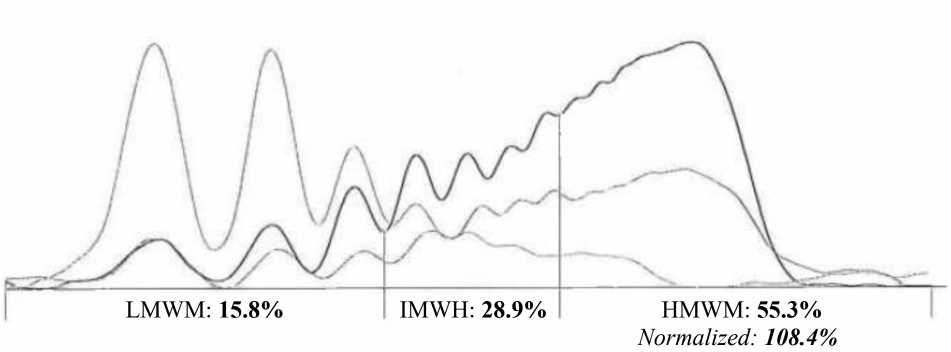

T4

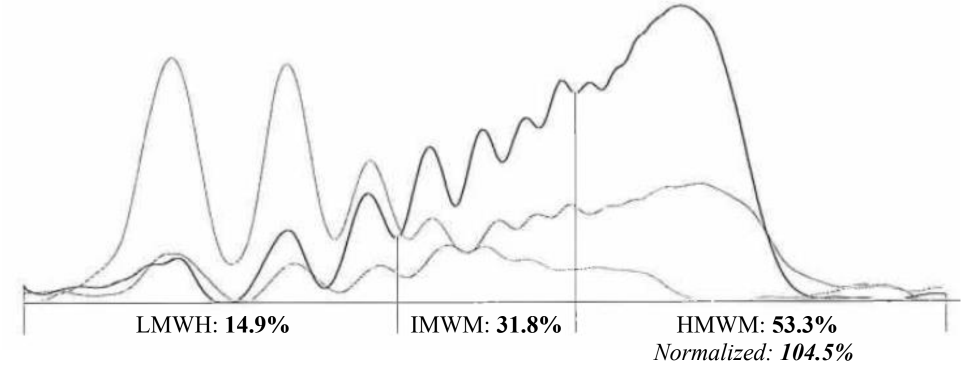

© 2024 Goltstein LMJ et al. *JAMA Network Open.*

Patient 13

T1

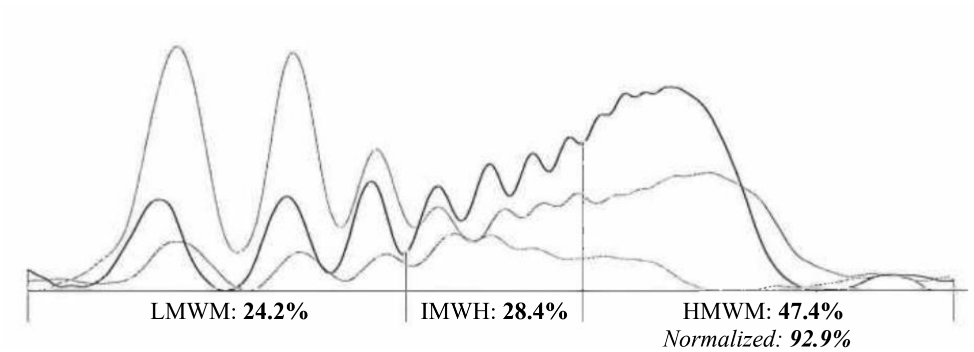

T2

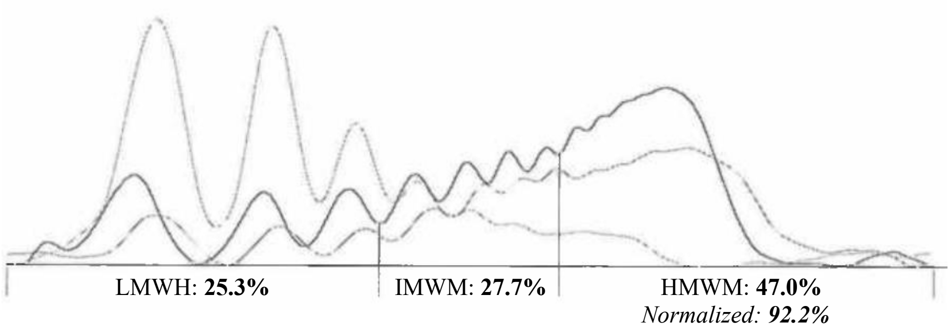

T3

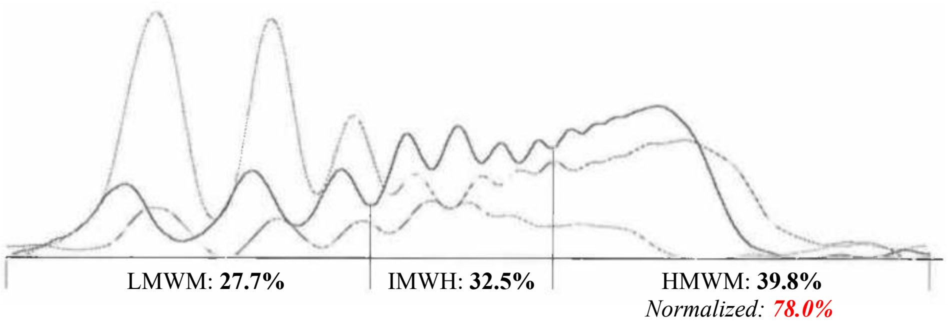

T4

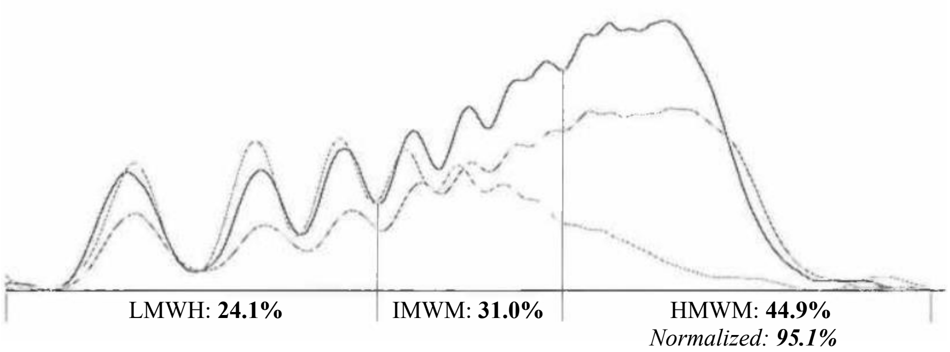

© 2024 Goltstein LMJ et al. *JAMA Network Open.*

Patient 14

T1

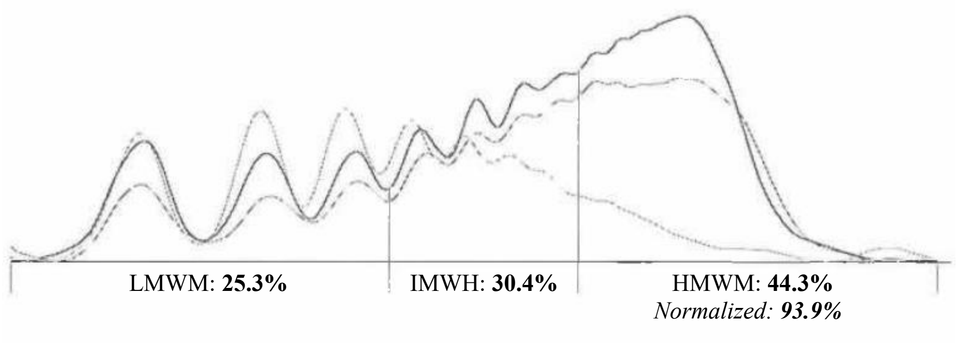

T2

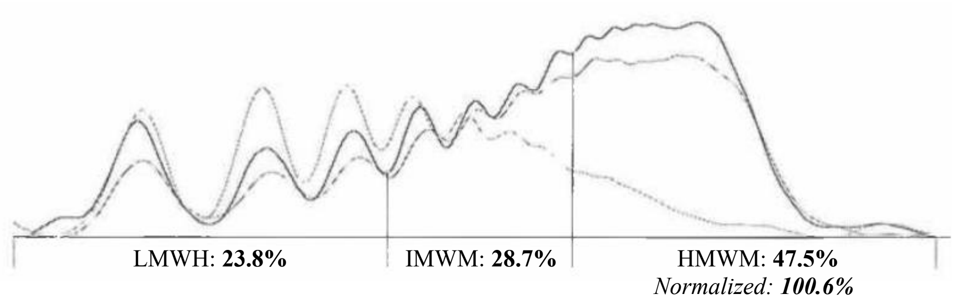

T3

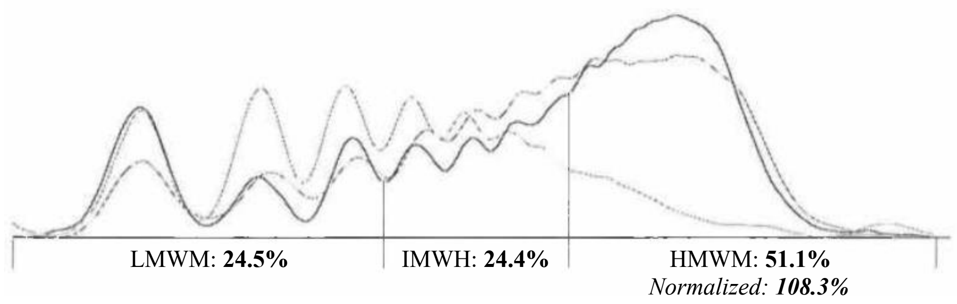

T4

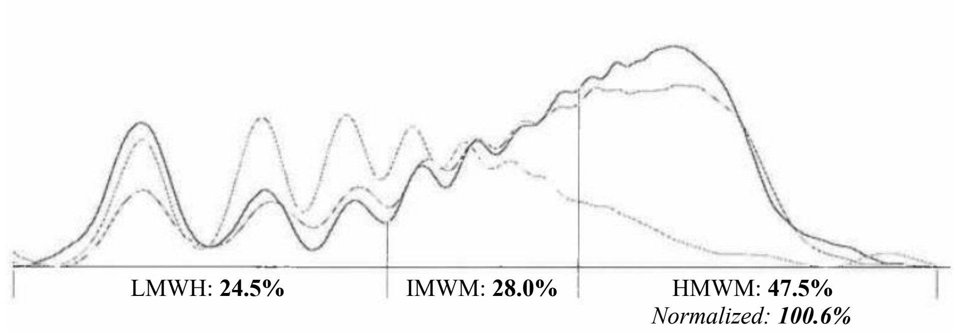

© 2024 Goltstein LMJ et al. *JAMA Network Open.*

Patient 15

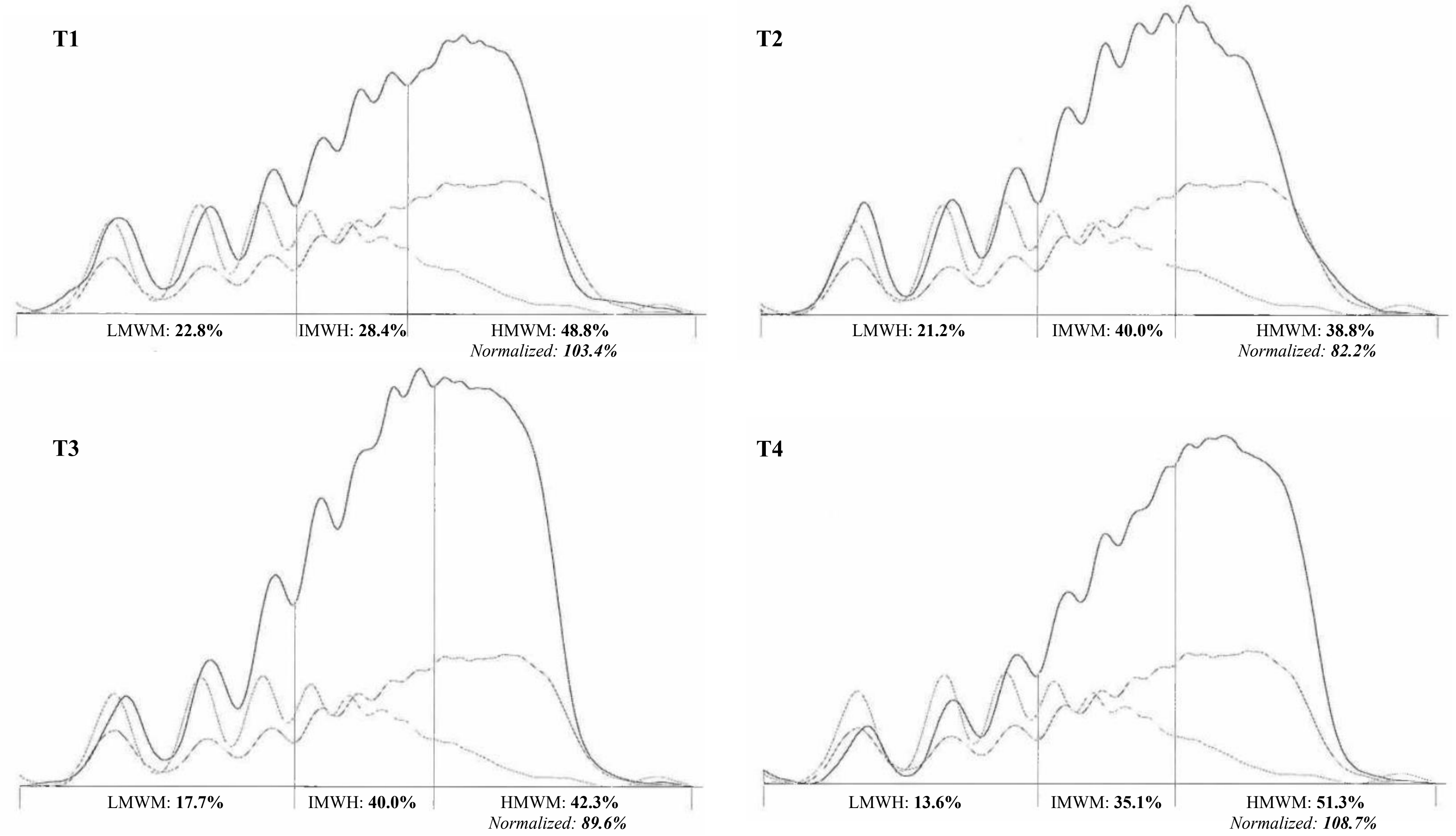

© 2024 Goltstein LMJ et al. *JAMA Network Open.*

Patient 21

T1

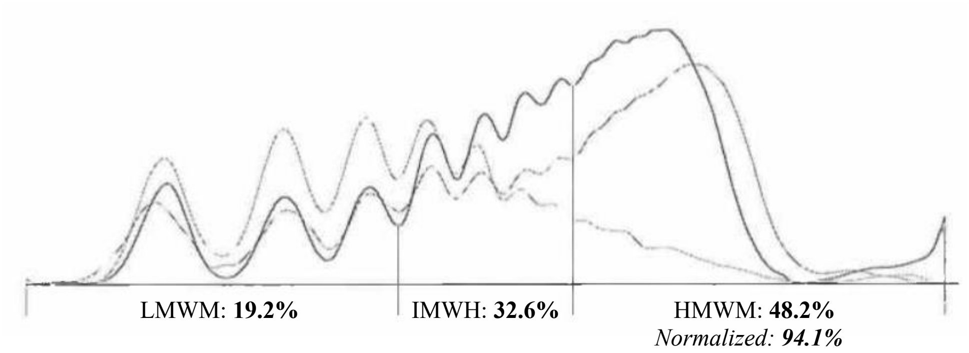

T2

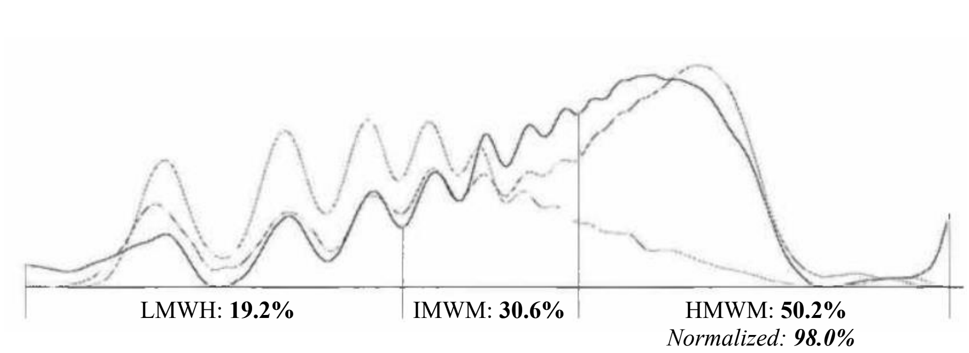

T3

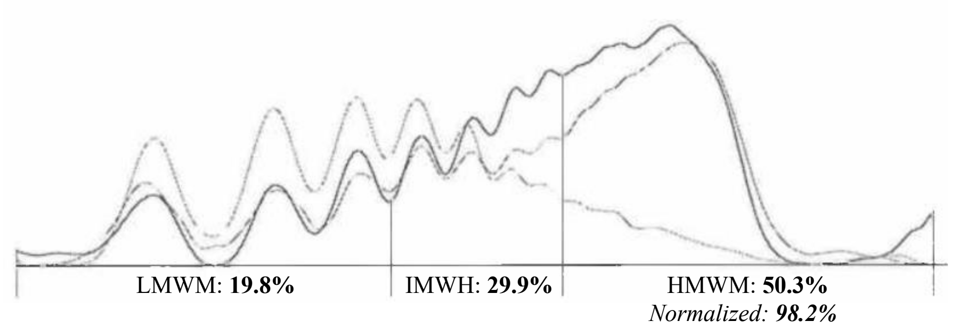

T4

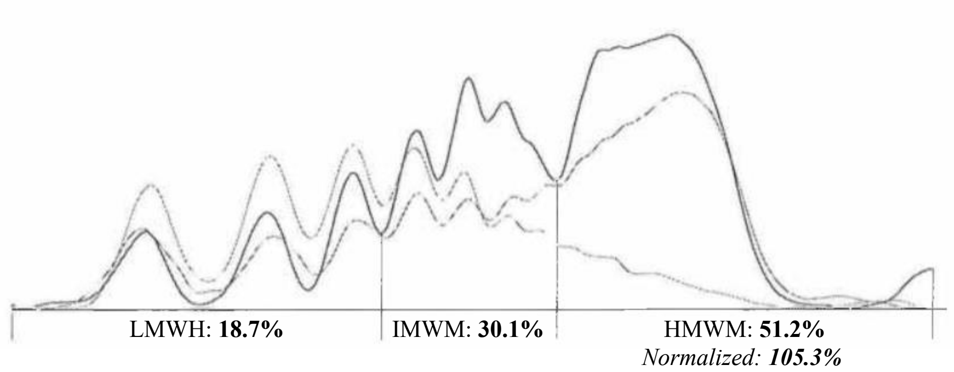

© 2024 Goltstein LMJ et al. *JAMA Network Open.*

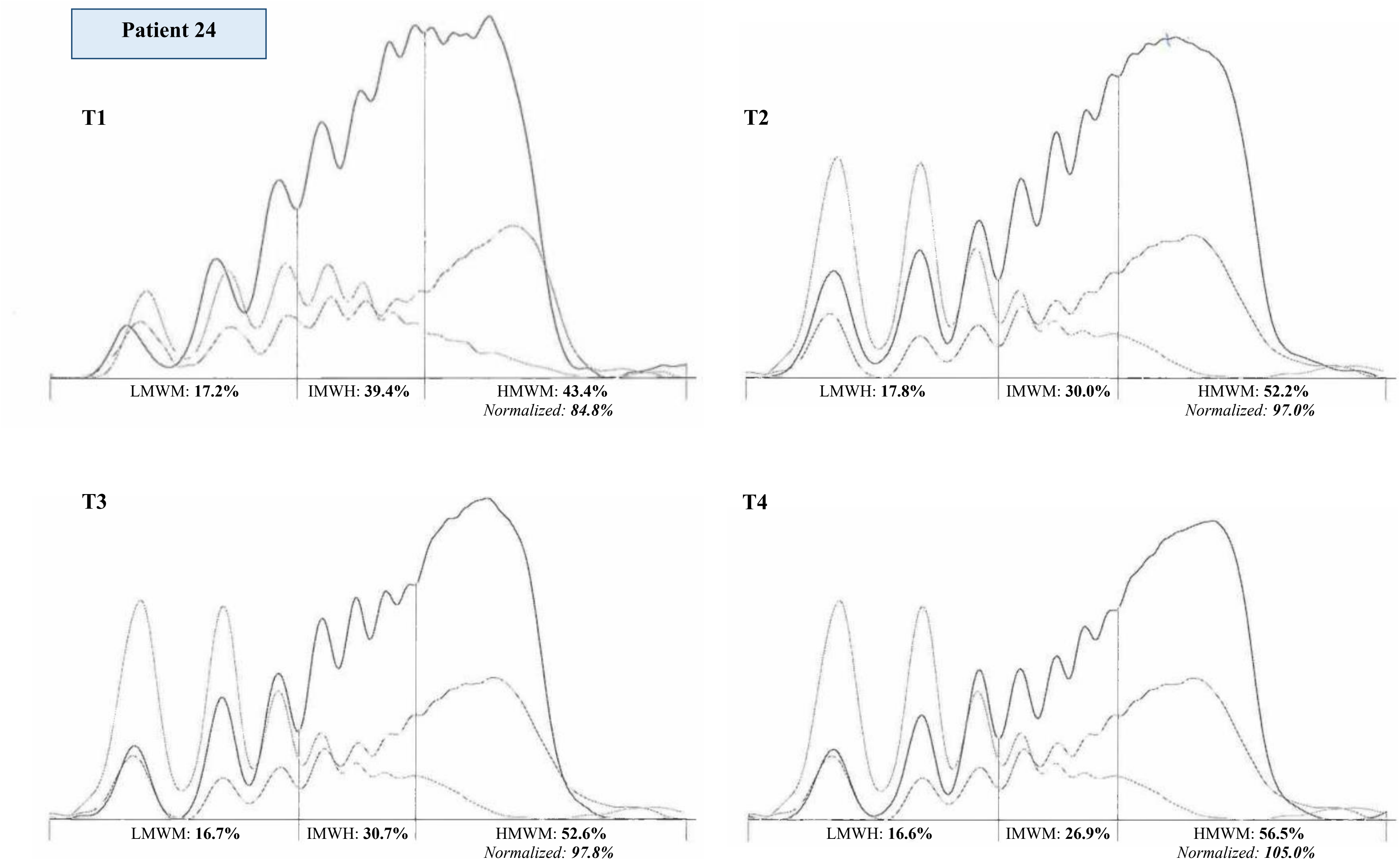

© 2024 Goltstein LMJ et al. *JAMA Network Open.*

Patient 5[a]

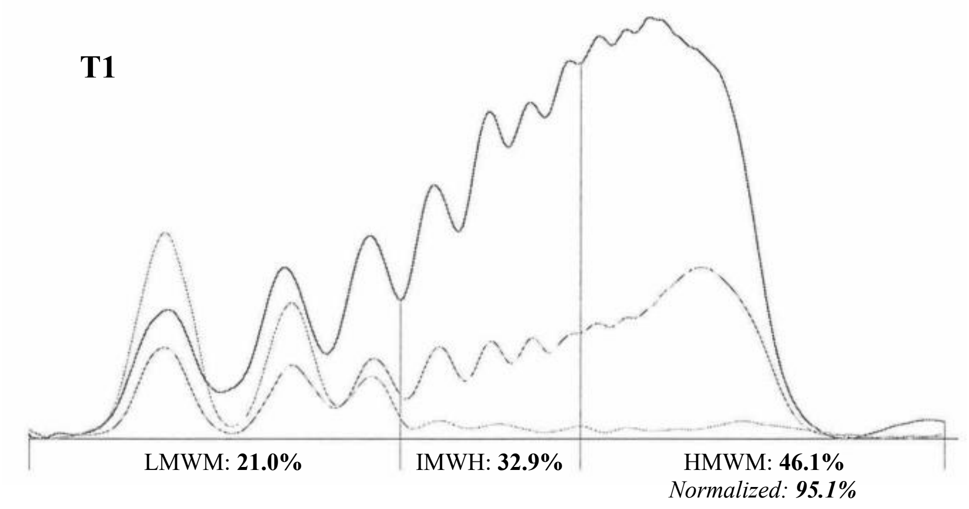

Patient 6[a]

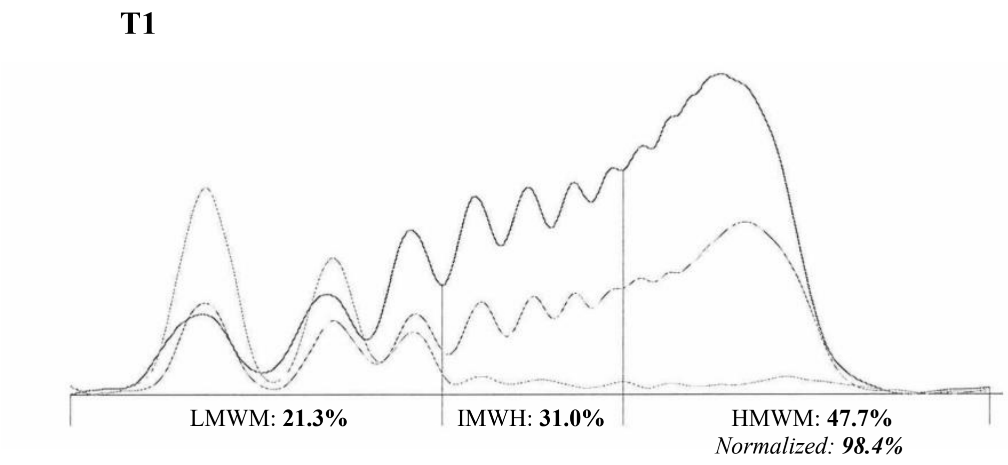

Patient 18[a]

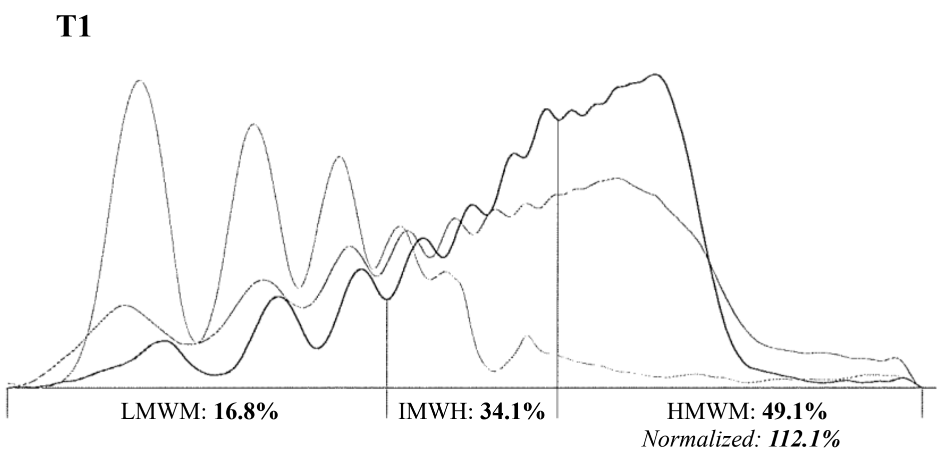

Patient 19[a]

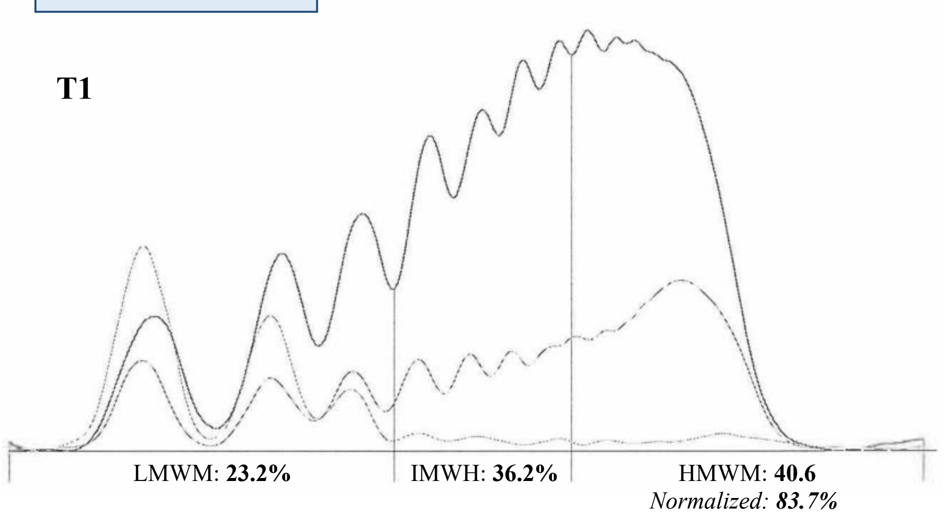

[a]Patients with Heyde syndrome who did not undergo a second capsule endoscopy after TAVI

© 2024 Goltstein LMJ et al. *JAMA Network Open.*

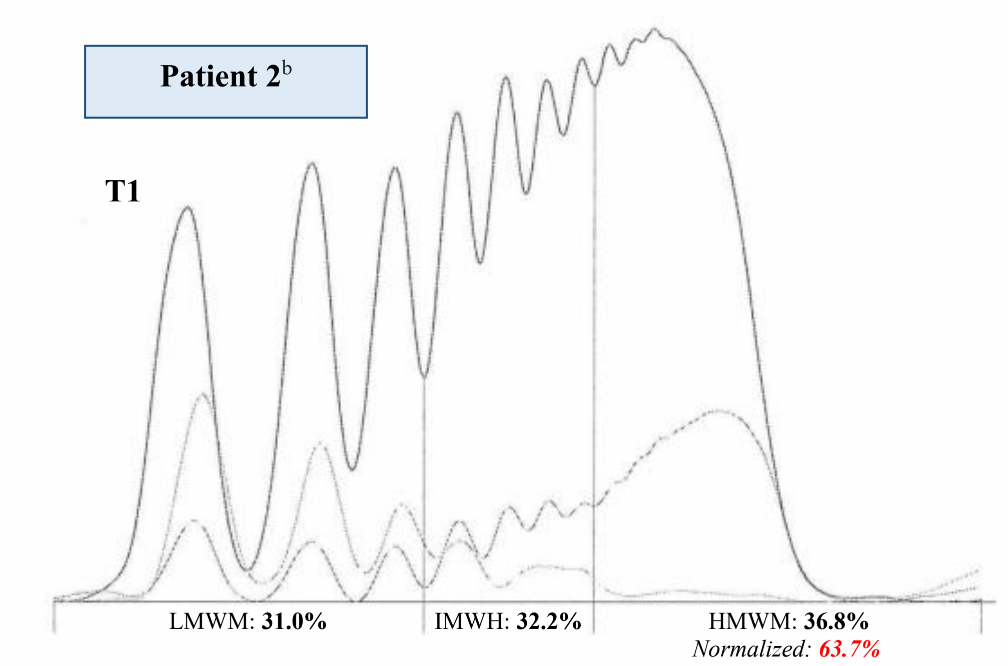

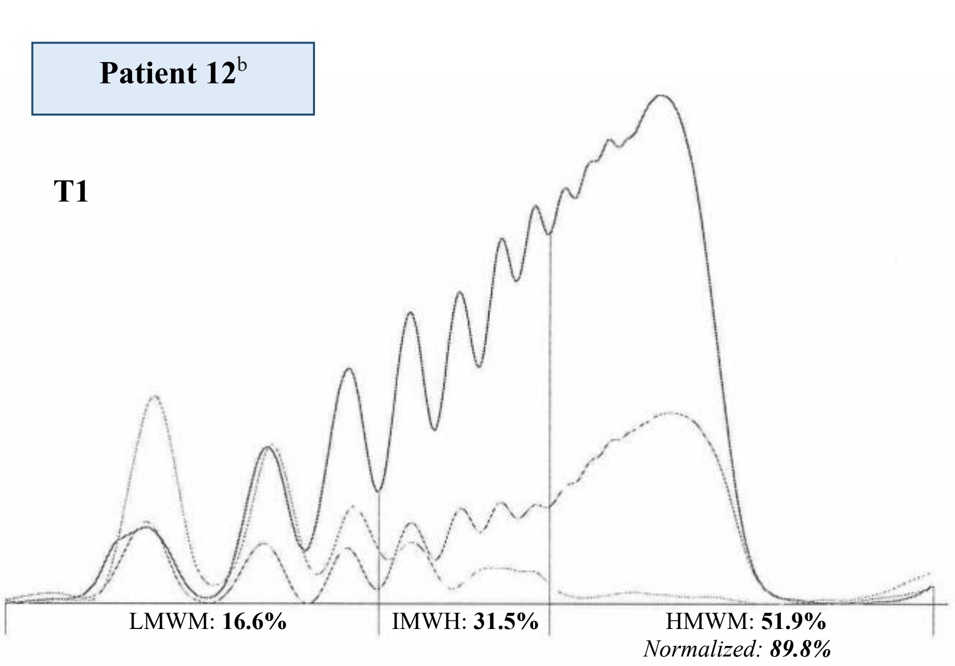

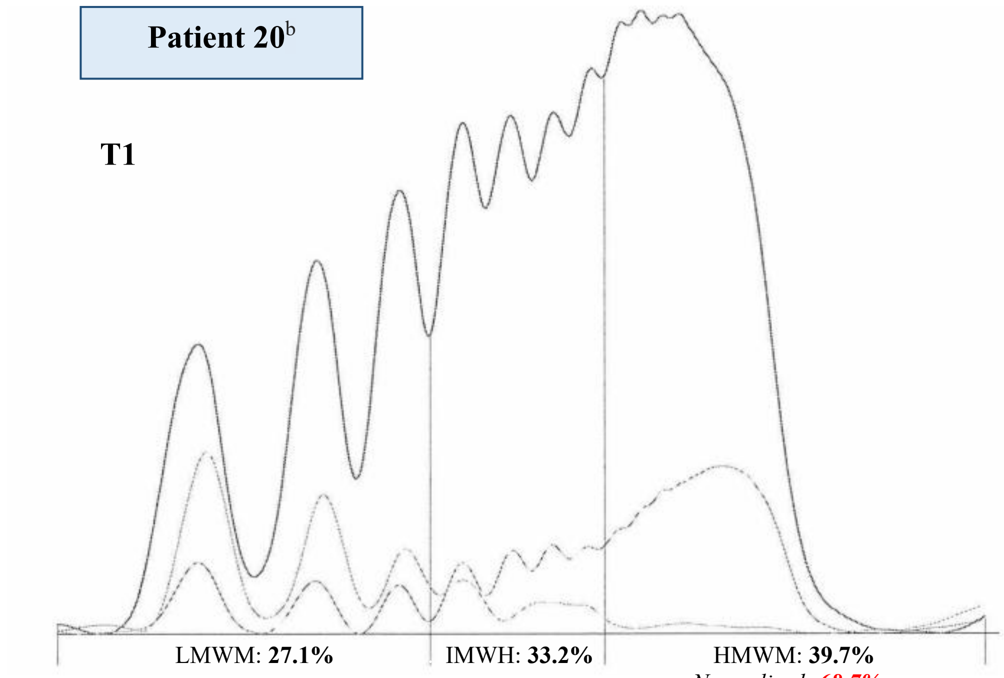

© 2024 Goltstein LMJ et al. *JAMA Network Open.*

[b]Patients with Heyde syndrome who were ineligible for TAVI

Patient 10[c]

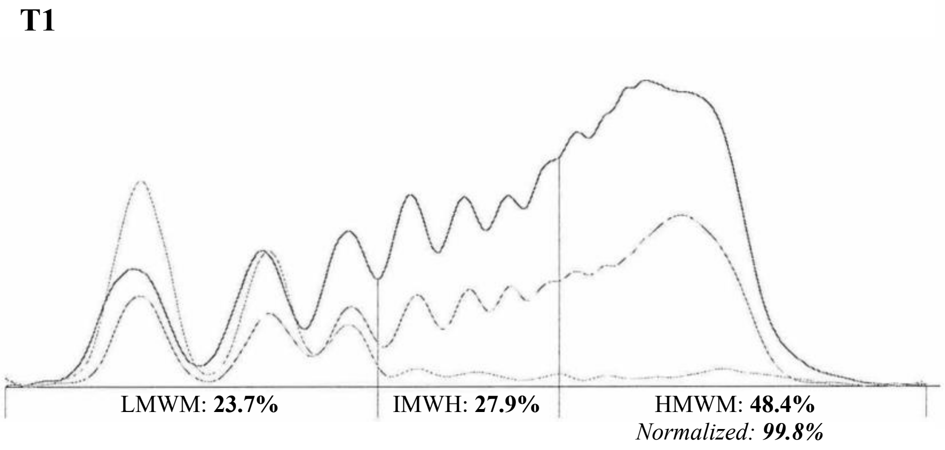

Patient 11[c]

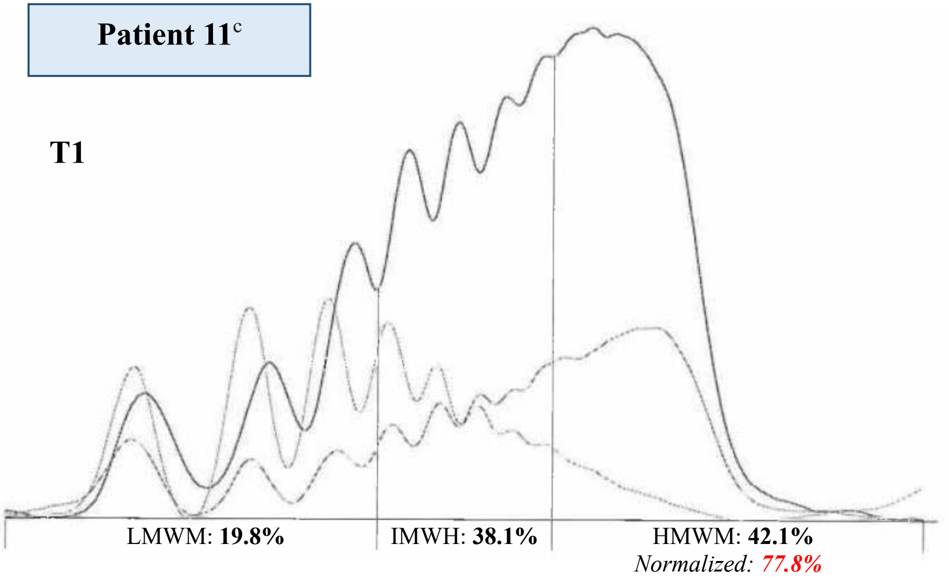

Patient 16[c]

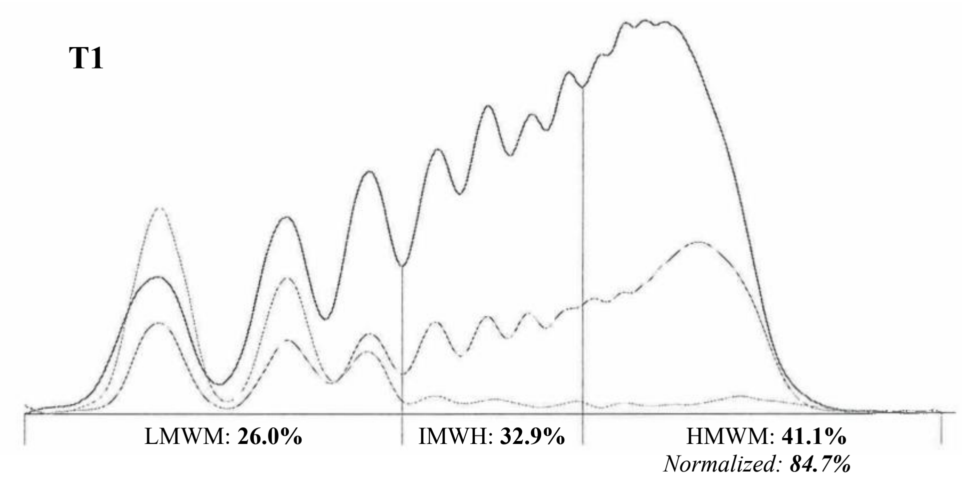

Patient 17[c]

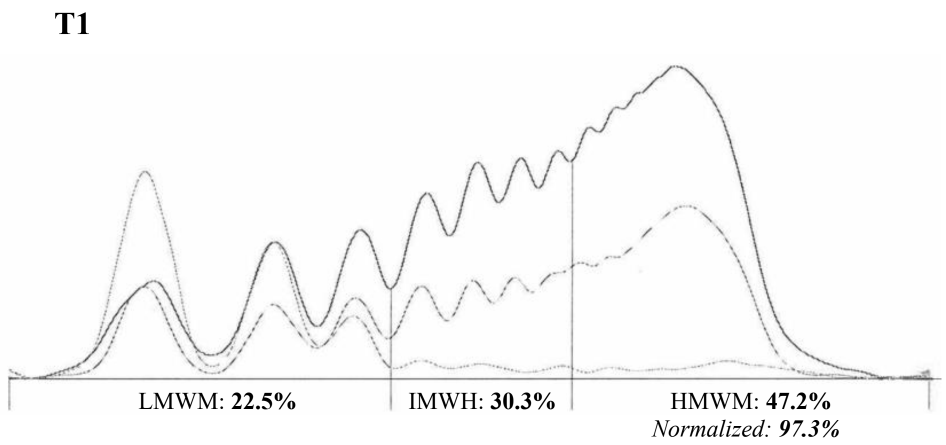

[c]Patients without Heyde syndrome

© 2024 Goltstein LMJ et al. *JAMA Network Open.*

Patient 22[c]

T1

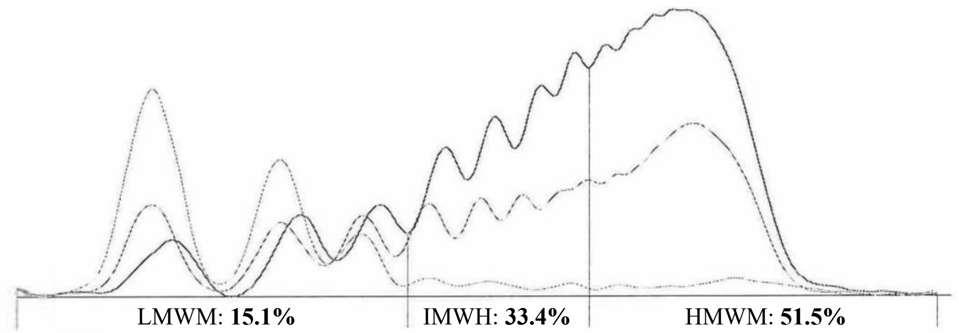

Patient 23[c]

T1

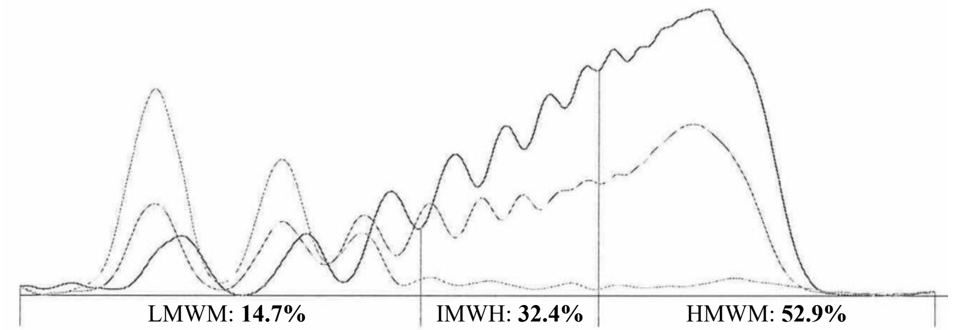

[c]Patients without Heyde syndrome

© 2024 Goltstein LMJ et al. *JAMA Network Open.*

eTable 4. Factors associated with angiodysplasia resolution after TAVI

**Characteristic**	**No angiodysplasias (N=6)**	**Angiodysplasias (N=5)[a]**
Multiple valvular heart disease, No. (%)		
• Isolated aortic stenosis	6 (100.0%)	2 (40%)
• Multiple valves	0 (0.0%)	3 (60.0%)
Paravalvular leakage, No. (%)		
• None to trace	4 (66.7%)	2 (40.0%)
• Mild to moderate	2 (33.3%)	3 (60.0%)
Acquired von Willebrand disease, No. (%)		
• Normalized vWF-HMWM <80%	1 (16.7%)	0 (0.0%)
• vWF:RCo/Ag ratio <0.7	0 (0.0%)	0 (0.0%)

Abbreviations: vWF, von Willebrand factor; vWF-HMWM, vWF high-molecular-weight multimers. vWF:RCo/Ag = vWF Ristocetin cofactor (vWF:RCo) to antigen (vWF:Ag) ratio.
[a]The patient who retained the same number of vascular lesions (one active bleeding in the colon) after TAVI was also included in the subgroup of patients with angiodysplasias at follow-up.

© 2024 Goltstein LMJ et al. *JAMA Network Open.*

References

1. Houdeville C, Souchaud M, Leenhardt R, Beaumont H, Benamouzig R, McAlindon M, et al. A multisystem-compatible deep learning-based algorithm for detection and characterization of angiectasias in small-bowel capsule endoscopy. A proof-of-concept study. *Dig Liver Dis*. 2021;53(12):1627-31.
2. Rosa B, Margalit-Yehuda R, Gatt K, Sciberras M, Girelli C, Saurin JC, et al. Scoring systems in clinical small-bowel capsule endoscopy: all you need to know! *Endosc Int Open*. 2021;9(6):E802-e23.
3. Leenhardt R, Li C, Koulaouzidis A, Cavallaro F, Cholet F, Eliakim R, et al. Nomenclature and semantic description of vascular lesions in small bowel capsule endoscopy: an international Delphi consensus statement. *Endosc Int Open*. 2019;7(3):E372-e9.
4. Leenhardt R, Koulaouzidis A, McNamara D, Keuchel M, Sidhu R, McAlindon ME, et al. A guide for assessing the clinical relevance of findings in small bowel capsule endoscopy: analysis of 8064 answers of international experts to an illustrated script questionnaire. *Clin Res Hepatol Gastroenterol.* 2021;45(6):101637.
5. Cortegoso Valdivia P, Deding U, Bjørsum-Meyer T, Baatrup G, Fernández-Urién I, Dray X, et al. Inter/Intra-Observer Agreement in Video-Capsule Endoscopy: Are We Getting It All Wrong? A Systematic Review and Meta-Analysis *Diagnostics (Basel)*. 2022;12(10):2400.
6. Oliver S, Vanniasinkam T, Mohammed S, Vong R, Favaloro EJ. Semi-automated von Willebrand factor multimer assay for von Willebrand disease: Further validation, benefits and limitations. *Int J Lab Hematol*. 2019;41(6):762-71.
7. Tamura T, Horiuchi H, Imai M, Tada T, Shiomi H, Kuroda M, et al. Unexpectedly High Prevalence of Acquired von Willebrand Syndrome in Patients with Severe Aortic Stenosis as Evaluated with a Novel Large Multimer Index. *J Atheroscler Thromb*. 2015;22(11):1115-23.
8. Yashige M, Inoue K, Zen K, Kobayashi R, Nakamura S, Fujimoto T, et al. Gastrointestinal Angiodysplasia before and after Treatment of Severe Aortic Stenosis. *N Engl J Med*. 2023;389(16):1530-2.

© 2024 Goltstein LMJ et al. *JAMA Network Open.*
