# Supplementary material for: Gastrointestinal Angiodysplasia Resolution After Transcatheter Aortic Valve Implantation
Source: JAMA Netw Open. 2024 Oct 30;7(10):e2442324. doi: 10.1001/jamanetworkopen.2024.42324 (PMC11525602; doi:10.1001/jamanetworkopen.2024.42324)
Supplement: Supplement 2. — Data Sharing Statement [file jamanetwopen-e2442324-s002.pdf]

Data Sharing Statement

Goltstein. Gastrointestinal Angiodysplasia Resolution After Transcatheter Aortic Valve Implantation. *JAMA Netw Open*. Published October 30, 2024. doi:10.1001/jamanetworkopen.2024.42324

Data
Data available: Yes
Data types: Deidentified participant data, Data (not involving human participants), Data dictionary
How to access data: Deidentified participant data will be available with publication (with no end date) upon reasonable request to Lia Goltstein (lia.goltstein@radboudumc.nl), subject to an appropriate data sharing agreement.
When available: With publication

Supporting Documents
Document types: Statistical/analytic code
How to access documents: Supporting documents will be available with publication (with no end date).
When available: With publication

Additional Information
Who can access the data: Researchers whose proposed use of the data has been approved.
Types of analyses: For research purposes (e.g., for an individual patient data meta-analysis).
Mechanisms of data availability: After approval of a proposal and with a signed data access agreement.
